# Auxiliary strategy for the general and practical synthesis of diaryliodonium(III) salts with diverse organocarboxylate counterions

**DOI:** 10.3762/bjoc.20.90

**Published:** 2024-05-03

**Authors:** Naoki Miyamoto, Daichi Koseki, Kohei Sumida, Elghareeb E Elboray, Naoko Takenaga, Ravi Kumar, Toshifumi Dohi

**Affiliations:** 1 College of Pharmaceutical Sciences, Ritsumeikan University, 1-1-1, Nojihigashi, Kusatsu Shiga, 525-8577, Japan; 2 Department of Chemistry, Faculty of Science, South Valley University, Qena 83523, Egypt; 3 Faculty of Pharmacy, Meijo University, 150 Yagotoyama, Tempaku-ku, Nagoya 468-8503, Japan; 4 Department of Chemistry, J. C. Bose University of Science & Technology, YMCA Faridabad, NH-2, Sector-6, Mathura Road, Faridabad, Haryana, 121006, India

**Keywords:** auxiliary ligand, diaryliodonium(III) salts, hybridization, hypervalent iodine, organocarboxylates

## Abstract

Diaryliodonium(III) salts are versatile reagents that exhibit a range of reactions, both in the presence and absence of metal catalysts. In this study, we developed efficient synthetic methods for the preparation of aryl(TMP)iodonium(III) carboxylates, by reaction of (diacetoxyiodo)arenes or iodosoarenes with 1,3,5-trimethoxybenzene in the presence of a diverse range of organocarboxylic acids. These reactions were conducted under mild conditions using the trimethoxyphenyl (TMP) group as an auxiliary, without the need for additives, excess reagents, or counterion exchange in further steps. These protocols are compatible with a wide range of substituents on (hetero)aryl iodine(III) compounds, including electron-rich, electron-poor, sterically congested, and acid-labile groups, as well as a broad range of aliphatic and aromatic carboxylic acids for the synthesis of diverse aryl(TMP)iodonium(III) carboxylates in high yields. This method allows for the hybridization of complex bioactive and fluorescent-labeled carboxylic acids with diaryliodonium(III) salts.

## Introduction

Hypervalent iodine compounds are an attractive class of reagents due to their stability, accessibility, and diverse chemical reactivity [1]. Diaryliodonium(III) salts, in particular, have been widely recognized as efficient arylating reagents for a range of carbon, nitrogen, oxygen, sulfur, and other nucleophiles, and can be employed in the presence or absence of transition metal catalysts under thermal or photochemical conditions [2–5]. Furthermore, these compounds have practical applications in the synthesis of radiochemicals utilized in positron emission tomography (PET) imaging [6], as well as serving as photoacid generators for photoinitiated radical polymerizations [7–8]. Consequently, there exists a growing interest in the development of more convenient synthetic routes for these compounds, facilitating the creation of structurally novel diaryliodonium(III) salts.

The counterions of diaryliodonium(III) salts play a crucial role in modifying their physical properties, and stability and controlling the reactivity of arylation processes, as demonstrated in various studies [9–10]. For instance, the Gaunt group reported that the use of a fluoride counterion in diaryliodonium(III) salt can trigger phenol *O*-arylation by activating the phenolic O–H group with a fluoride anion [11]. Additionally, Muñiz et al. found that the acetate counterion was more effective than chloride, hexafluorophosphate, and trifluoromethane sulfonate for the borylation of diaryliodonium(III) salts [12]. Recently, our group has developed a new method for phenol *O*-arylation using aryl(2,4,6-trimethoxyphenyl)iodonium(III) acetates [13]. In this process, the acetate ligand acted as a base to activate the phenol group and positioned it in proximity to accomplish the smooth S_N_Ar reaction.

**Scheme 1:**
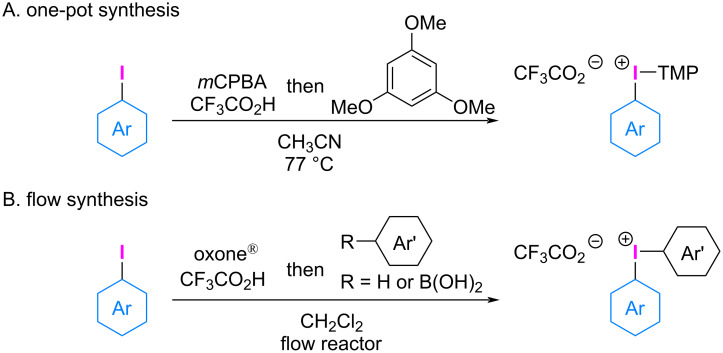
Synthetic approaches of diaryliodonium(III) trifluoroacetates.

The synthesis of diaryliodonium(III) salts with various counterions, such as triflate (TfO^−^) [14], tetrafluoroborate (BF_4_^−^) [15], tosylate (TsO^−^) [16], and others [17], has been extensively studied, as they play a key factor in the participation of iodonium salts in diverse arylation reactions. Recently, efficient syntheses of diaryliodonium(III) trifluoroacetates have been reported [18–19] (Scheme 1). The importance of the trimethoxyphenyl (TMP) group as an auxiliary (dummy) ligand on the iodonium salt has prompted researchers to synthesize aryl(TMP)iodonium(III) trifluoroacetates via oxidation of iodoarene with *m*-chloroperbenzoic acid (*m*CPBA) in the presence of trifluoroacetic acid, followed by coupling with 1,3,5-trimethoxybenzene [18] (Scheme 1A). This process demonstrated tolerance for a wide range of electron-rich and electron-deficient (hetero)aryl iodine(III) compounds. Wirth and colleagues reported the flow synthesis of diaryliodonium(III) trifluoroacetates using a cartridge filled with powdered oxone^®^ for in situ generation of bis(trifluoroacetoxyiodo)arenes and their reaction with electron-rich arene or arylboronic acid [19] (Scheme 1B).

**Scheme 2:**
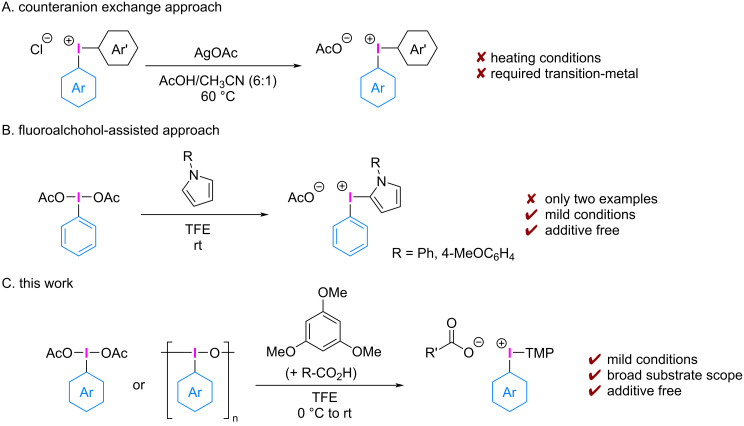
Synthesis of diaryliodonium(III) carboxylates.

Carboxylic acids, such as acetic acid and benzoic acid, characterized by substantial difference in p*K*_a_ values when compared to trifluoroacetic acid, TfOH, HBF_4_, and *p*-TsOH, present a wider substrate scope, including acid-sensitive groups, in the preparation of diaryliodonium(III) salts. While the counterion exchange of diaryliodonium(III) chloride with silver acetate was reported [20], this method required heating conditions and the use of an equimolar amount of the metal salt (Scheme 2A). Despite the expected advantage, direct synthesis of these diaryliodonium(III) carboxylates are scarce, and these compounds were synthesized by reacting (diacetoxyiodo)benzene and *N*-functionalized pyrrole in 2,2,2-trifluoroethanol (TFE, Scheme 2B) [21].

Our group previously reported the synthesis of diaryliodonium(III) salts by combining hypervalent iodine(III) reagents with electron-rich arenes in fluoroalcohol solvents, such as TFE or 1,1,1,3,3,3-hexafluoro-2-propanol [21–22]. These solvents stabilize the cationic intermediates in the synthesis of diaryliodonium(III) salts from Koser's reagents or (diacetoxyiodo)arenes. While iodonium salts with TfO^−^, TsO^−^, and other counterions are common, the related diaryliodonium carboxylates are more attractive from a cost and safety standpoint. In this work, we present a more practical, direct approach for the synthesis of aryl(TMP)iodonium(III) carboxylates, utilizing readily accessible iodosoarenes or (diacetoxyiodo)arenes as starting materials in a fluoroalcohol solvent (Scheme 2C). This protocol allows for the synthesis of electron-rich, electron-poor, heterocyclic, and sterically hindered aryl(TMP)iodonium(III) carboxylates by combining the broad substrate scope of (hetero)aryl iodine(III) and carboxylic acids under mild conditions.

## Results and Discussion

In the synthesis of diaryliodonium(III) salts and their application in arylation reactions, it is highly desirable to design diaryliodonium(III) salts including a commercially available and inexpensive auxiliary group to achieve efficient preparation of the salts and a high degree of chemoselectivity for transferring the required aryl group. Electron-rich aryl ligands derived from anisole, mesitylene, and particularly 1,3,5-trimethoxybenzene are highly recommended for chemoselective arylation processes. Aryl(TMP)iodonium(III) salts have been successfully used as transition metal-free arylating reagents for various nucleophiles such as nitrogen- [23–26], oxygen- [13,27–29], sulfur- [30], and carbon- [31] nucleophiles due to their excellent reactivity and aryl group selectivity over aryl(anisyl)iodonium(III) salts [32] and aryl(mesityl)iodonium(III) salts [33].

**Scheme 3:**
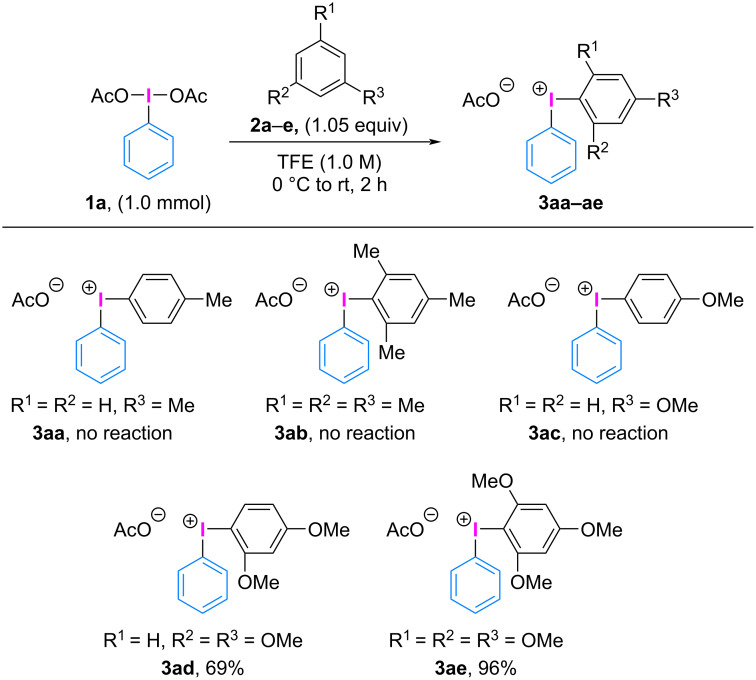
Scope of dummy ligands.

Based on our previously reported conditions for the synthesis of diaryliodonium(III) salts [21], we designed a more practical synthetic protocol for the extended numbers of diaryliodonium(III) carboxylates. Various electron-rich arenes were screened as auxiliary aryl groups in the reaction with PhI(OAc)_2_ (**1a**) (Scheme 3). However, common partners such as toluene (**2a**), mesitylene (**2b**), and anisole (**2c**) failed to react with PhI(OAc)_2_ (**1a**). Therefore, 1,3-dimethoxybenzene (**2d**) was used as a more electron-rich aryl group in the reaction with PhI(OAc)_2_ (**1a**), resulting in the formation of the desired phenyl(2,4-dimethoxyphenyl)iodonium(III) acetate (**3ad**) in 69% yield. Notably, utilizing 1,3,5-trimethoxybenzene (**2e**) as an auxiliary aryl group under identical conditions yielded the corresponding phenyl(TMP)iodonium(III) acetate (**3ae**) in 96% yield.

**Scheme 4:**
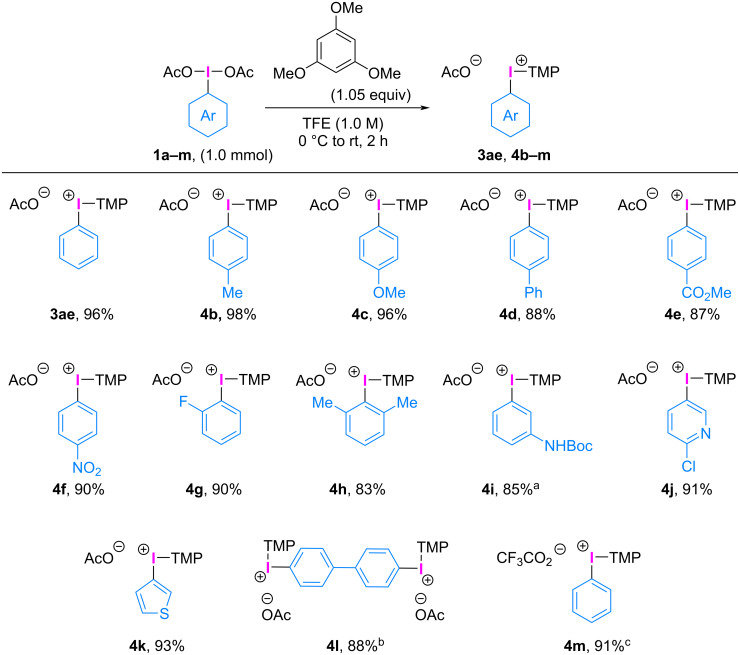
Substrate scope of aryl(TMP)iodonium(III) acetates. a) 0.50 mmol scale of **1i**. b) 1,3,5-Trimethoxybenzene (2.1 equiv) was used. c) Bis(trifluoroacetoxyiodo)benzene was used instead of **1a**.

Utilizing TMP as an auxiliary aryl group, we investigated the substrate scope of (diacetoxyiodo)arenes **1** for the synthesis of aryl(TMP)iodonium(III) acetates **4** (Scheme 4). The starting materials, (diacetoxyiodo)arenes **1**, can be prepared through the oxidation of iodoarenes with NaBO_3_·4H_2_O [34], AcOOH [35], *m*CPBA [36], and NaClO·5H_2_O [37] in the presence of acetic acid. The reaction of (diacetoxyiodo)arenes bearing electron-donating (methyl (**1b**), methoxy (**1c**), and phenyl (**1d**)) and electron-withdrawing (methyl ester (**1e**), nitro (**1f**), and fluoro (**1g**)) groups proceeded efficiently to produce the corresponding aryl(TMP)iodonium(III) acetates **4b**–**g** in high yields. A sterically hindered *ortho*-disubstituted aryl group was also well-tolerated, and the related *ortho*-disubstituted aryl(TMP)iodonium(III) acetate (**4h**) was obtained in 83% yield. Notably, this strategy allowed the synthesis of (diacetoxyiodo)arenes bearing acid-sensitive Boc protecting groups (**1i**) and heteroaromatic moieties such as pyridyl (**1j**) and thienyl (**1k**) groups. The reaction of bis(diacetoxyiodo)arene (**1l**) with 1,3,5-trimethoxybenzene (2.1 equiv) under the same conditions afforded the ditrigger iodonium salt **4l** in 88% yield, demonstrating the versatility of the process for the synthesis of multivalent precursors. Furthermore, phenyliodine(III) bis(trifluoroacetate) was used as a starting material under the optimized reaction conditions and the corresponding phenyl(TMP)iodonium(III) trifluoroacetate (**4m**) was obtained in 91% yield. These aryl(TMP)iodonium(III) acetates were recently utilized by our group for the arylation of phenols [13] and *N*-alkoxyamides [26,29], exhibiting excellent reactivity and aryl group selectivity.

**Scheme 5:**
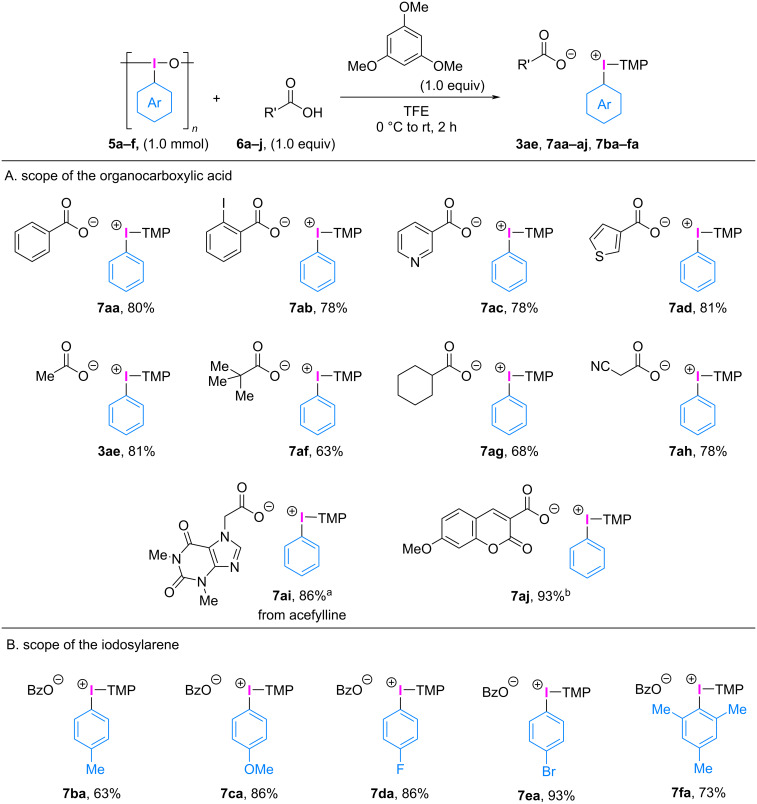
Substrate scope of the carboxylic acids and iodosylarenes. a) The reaction was conducted for 4 h. b) 2.0 mL TFE was used.

In subsequent experiments, we sought to develop an alternative approach by reacting iodosobenzene (**5a**) with a range of aromatic and aliphatic carboxylic acids **6a**–**i** to form phenyl(TMP)iodonium(III) carboxylates **3ae**, **7aa**–**ai** (Scheme 5A). The reaction between benzoic acids (**6a**, **6b**) and heteroaromatic carboxylic acids (**6c**, **6d**) proceeded smoothly under the set conditions to form the corresponding phenyl(TMP)iodonium(III) carboxylates **7aa**–**ad** in high yield. Additionally, a range of aliphatic carboxylic acids such as acetic acid (**6e**), pivalic acid (**6f**), cyclohexanecarboxylic acid (**6g**), and aliphatic carboxylic acid with acidic α-proton (**6h**) was also tolerated under these conditions to produce the corresponding phenyl(TMP)iodonium(III) carboxylates (**3ae, 7af**–**ah**) in 63–81% yield without any signs of side reactions. The adenosine receptor antagonist acefylline (**6i**) was also used as a carboxylic acid to give the corresponding phenyl(TMP)iodonium(III) carboxylate **7ai** in 86% yield, opening up new avenues for structural modifications of drug candidates to improve their properties and consequently, bioactivities [38–40]. The umbelliferone-3-carboxylic acid derivative **6j** was also employed to produce the phenyl(TMP)iodonium(III) carboxylate **7aj** carrying a fluorescent-labeling group in 93% yield.

Iodosoarenes **5b**–**f** can be easily obtained by treating (dichloroiodo)arenes [41] or (diacetoxyiodo)arenes [42] with sodium hydroxide, by oxidation of iodoarenes with NaClO·5H_2_O [43], or by electrolysis [44]. The reaction scope of iodosoarenes **5b**–**f** was explored with benzoic acid (**6a**) and 1,3,5-trimethoxybenzene (Scheme 5B). Iodosoarenes with electron-rich (**5b**, **5c**, **5f**), electron-deficient (**5d**), bromo (**5e**), and sterically hindered substituents (**5f**) were applicable to give the corresponding aryl(TMP)iodonium(III) benzoates **7ba**–**fa** in 63–93% yield. It is worth noting that the products yielded by these protocols were easily separated as white amorphous solids by concentration and trituration of the obtained residue with diethyl ether. The color of the products indicates that the reactions proceeded without any signs of decomposition, consequently yielding the desired products in high yields. However, the common synthetic methods of diaryliodonium(III) triflates involving a strong oxidizing agent with a strong acid and an electron-rich arene often resulted in black/discolored products, indicating decomposition, poor yields, and lower productivity in arylation processes [45].

**Scheme 6:**
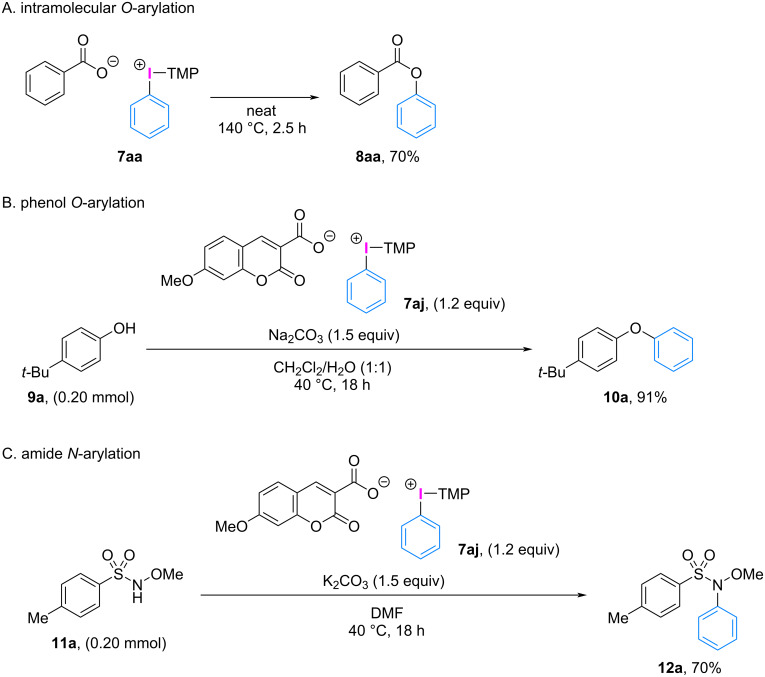
Representative applications of aryl(TMP)iodonium(III) carboxylates.

These aryl(TMP)iodonium(III) carboxylates are stable at room temperature and are available as amorphous solids that dissolve in specific solvents, such as chloroform, methanol, and dimethyl sulfoxide. The iodonium salt **7aa** does not decompose even at 70 °C, and further increase in temperature facilitates the ligand coupling between the phenyl group and the carboxylate counterion. When heated at 140 °C for 2.5 h under solvent-free conditions, iodonium salt **7aa** underwent carboxylate *O*-phenylation with complete phenyl group transfer, resulting in the formation of phenyl benzoate in 70% yield (Scheme 6A).

Furthermore, iodonium salt **7aj** with an umbelliferone-3-carboxylate counterion displayed extremely weak blue fluorescence emission under 365 nm UV light compared to free carboxylic acid **6j**. This unique property was utilized for tracing the counterion exchange process of the diaryliodonium(III) salt by irradiating with 365 nm UV light. The counterion exchange in umbelliferone carboxylate salt **7aj** with trifluoroacetic acid was rapid, and after 30 s, the completion of the reaction was confirmed by the emergence of strong blue fluorescence emission due to the liberation of the fluorescent-labeling carboxylic acid **6j** (see Supporting Information File 1, Figure S1). Thus, this post-fluorescence iodonium salt can be used for visual indication of the ligand exchange process, elucidating the arylation mechanism of diaryliodonium(III) salts for their further applications in organic chemistry and other scientific fields. The potential of diaryliodonium(III) carboxylates obtained in this study (i.e., Scheme 6B,C) and the related amino acid derivatives [46–49] as new arylating reagents will be further explored by conducting reactions with various nucleophiles involving the counterion exchange process.

## Conclusion

The absence of a widely applicable method for the synthesis of diaryliodonium(III) carboxylates has prompted our research group to devise a practical strategy for the synthesis of aryl(TMP)iodonium(III) carboxylates with minimal reagents without a counterion exchange step. By employing TMP as an auxiliary aryl group, we have successfully achieved the reaction between the hypervalent iodine compounds (ArI(OAc)_2_ or ArIO) and 1,3,5-trimethoxybenzene in the presence of organocarboxylic acid under mild conditions. This process was completed in comparatively shorter time at room temperature, yielding high yields of the corresponding aryl(TMP)iodonium(III) carboxylates. Our method is compatible with a wide range of electronically and sterically diverse (hetero)aryl iodine(III) compounds, as well as aliphatic and aromatic carboxylic acids with a diverse series of functional groups. As a result, this process can be applied for the unique hybridization of biologically active and fluorescently-labeled carboxylic acids with diaryliodonium(III) salts. We anticipate that this study will encourage the incorporation of diaryliodonium(III) carboxylates in various new applications.

## Supporting Information

File 1 Further experimental details and copies of ^1^H, ^13^C, and ^19^F NMR spectra.

## Data Availability

All data that supports the findings of this study is available in the published article and/or the supporting information to this article.

## References

[1] Stang P J, Zhdankin V V (1996). Chem Rev.

[2] Merritt E A, Olofsson B (2009). Angew Chem, Int Ed.

[3] Kikushima K, Elboray E E, Jiménez-Halla J O C, Solorio-Alvarado C R, Dohi T (2022). Org Biomol Chem.

[4] Senapati S, Parida S K, Karandikar S S, Murarka S (2023). Org Lett.

[5] Meher P, Panda S P, Mahapatra S K, Thombare K R, Roy L, Murarka S (2023). Org Lett.

[6] Rong J, Haider A, Jeppesen T E, Josephson L, Liang S H (2023). Nat Commun.

[7] Crivello J V, Lam J H W (1977). Macromolecules.

[8] Honma H, Yasue R, Ichikawa K (2023). Aromatic heterocyclic compound acid generator, photoresist compositions, resist patterns formed thereby. Jap. Patent.

[9] Seidl T L, Sundalam S K, McCullough B, Stuart D R (2016). J Org Chem.

[10] Seidl T L, Stuart D R (2017). J Org Chem.

[11] Chan L, McNally A, Toh Q Y, Mendoza A, Gaunt M J (2015). Chem Sci.

[12] Miralles N, Romero R M, Fernández E, Muñiz K (2015). Chem Commun.

[13] Kikushima K, Miyamoto N, Watanabe K, Koseki D, Kita Y, Dohi T (2022). Org Lett.

[14] Bielawski M, Zhu M, Olofsson B (2007). Adv Synth Catal.

[15] Bielawski M, Aili D, Olofsson B (2008). J Org Chem.

[16] Zhu M, Jalalian N, Olofsson B (2008). Synlett.

[17] Beringer F M, Drexler M, Gindler E M, Lumpkin C C (1953). J Am Chem Soc.

[18] Carreras V, Sandtorv A H, Stuart D R (2017). J Org Chem.

[19] Soldatova N S, Postnikov P S, Yusubov M S, Wirth T (2019). Eur J Org Chem.

[20] Beringer F M, Galton S A, Huang S J (1962). J Am Chem Soc.

[21] Dohi T, Yamaoka N, Kita Y (2010). Tetrahedron.

[22] Dohi T, Hayashi T, Ueda S, Shoji T, Komiyama K, Takeuchi H, Kita Y (2019). Tetrahedron.

[23] Sandtorv A H, Stuart D R (2016). Angew Chem, Int Ed.

[24] Basu S, Sandtorv A H, Stuart D R (2018). Beilstein J Org Chem.

[25] Roshandel S, Lunn M J, Rasul G, Muthiah Ravinson D S, Suri S C, Prakash G K S (2019). Org Lett.

[26] Kikushima K, Morita A, Elboray E E, Bae T, Miyamoto N, Kita Y, Dohi T (2022). Synthesis.

[27] Dohi T, Koseki D, Sumida K, Okada K, Mizuno S, Kato A, Morimoto K, Kita Y (2017). Adv Synth Catal.

[28] Gallagher R T, Basu S, Stuart D R (2020). Adv Synth Catal.

[29] Elboray E E, Bae T, Kikushima K, Kita Y, Dohi T (2023). Adv Synth Catal.

[30] Saikia R A, Hazarika N, Biswakarma N, Chandra Deka R, Thakur A J (2022). Org Biomol Chem.

[31] Kikushima K, Yamada K, Umekawa N, Yoshio N, Kita Y, Dohi T (2023). Green Chem.

[32] Jalalian N, Petersen T B, Olofsson B (2012). Chem – Eur J.

[33] Qian X, Han J, Wang L (2016). Adv Synth Catal.

[34] McKillop A, Kemp D (1989). Tetrahedron.

[35] Dohi T, Yamaoka N, Itani I, Kita Y (2011). Aust J Chem.

[36] Iinuma M, Moriyama K, Togo H (2012). Synlett.

[37] Watanabe A, Miyamoto K, Okada T, Asawa T, Uchiyama M (2018). J Org Chem.

[38] Nassar A F, Nassar A F, Hollenberg P F, Scatina J (2023). Role of Structural Modifications of Drug Candidates to Enhance Metabolic Stability. Drug Metabolism Handbook: Concepts and Applications in Cancer Research.

[39] Nassar A F, Nassar A F, Hollenberg P F, Scatina J (2023). Drug Design Strategies: Role of Structural Modifications of Drug Candidates to Improve PK Parameters of New Drugs. Drug Metabolism Handbook: Concepts and Applications in Cancer Research.

[40] Nassar A F, Nassar A F, Hollenberg P F, Scatina J (2023). Chemical Structural Alert and Reactive Metabolite Concept as Applied in Medicinal Chemistry to Minimize the Toxicity of Drug Candidates. Drug Metabolism Handbook: Concepts and Applications in Cancer Research.

[41] Lucas H J, Kennedy E R, Formo M W (1942). Org Synth.

[42] Saltzman H, Sharefkin J G (1963). Org Synth.

[43] Miyamoto K, Watanabe Y, Takagi T, Okada T, Toyama T, Imamura S, Uchiyama M (2021). ARKIVOC.

[44] Zu B, Ke J, Guo Y, He C (2021). Chin J Chem.

[45] Linde E, Mondal S, Olofsson B (2023). Adv Synth Catal.

[46] Koposov A Y, Boyarskikh V V, Zhdankin V V (2004). Org Lett.

[47] Li H, Gori D, Kouklovsky C, Vincent G (2010). Tetrahedron: Asymmetry.

[48] Islam M, Tirukoti N D, Nandi S, Hotha S (2014). J Org Chem.

[49] Kishore Vandavasi J, Hu W-P, Chandru Senadi G, Chen H-T, Chen H-Y, Hsieh K-C, Wang J-J (2015). Adv Synth Catal.

