# Supplementary material for: Auxiliary strategy for the general and practical synthesis of diaryliodonium(III) salts with diverse organocarboxylate counterions
Source: Beilstein J Org Chem. 2024 May 3;20:1020–8. doi: 10.3762/bjoc.20.90 (PMC11070968; doi:10.3762/bjoc.20.90)

BEILSTEIN JOURNAL OF ORGANIC CHEMISTRY

Supporting Information

for

Auxiliary strategy for the general and practical synthesis of diaryliodonium(III) salts with diverse organocarboxylate counterions

Naoki Miyamoto, Daichi Koseki, Kohei Sumida, Elghareeb E. Elboray, Naoko Takenaga, Ravi Kumar and Toshifumi Dohi

Beilstein J. Org. Chem. **2024,** *20,* 1020–1028. doi:10.3762/bjoc.20.90

Further experimental details and copies of ^{1}H, ^{13}C, and ^{19}F NMR spectra

License and Terms: This is a supporting information file under the terms of the Creative Commons Attribution License (https://creativecommons.org/licenses/by/4.0). Please note that the reuse, redistribution and reproduction in particular requires that the author(s) and source are credited and that individual graphics may be subject to special legal provisions.
The license is subject to the *Beilstein Journal of Organic Chemistry* terms and conditions: (https://www.beilstein-journals.org/bjoc/terms)

Table of Contents

1. General Information ······························ S2

2. General Procedure for the synthesis of diaryliodonium(III) salts ·············· S3–S20

3. Arylation of various nucleophiles ······························ S21–S23

4. Counterion exchange reaction ······························ S24

5. References ······························ S25

6. NMR Charts ······························ S26–S90

1. General Information

Experiment and analysis: Flash column chromatography and analytical TLC were carried out on Merck Silica gel 60 (230–400 mesh) and Merck Silica gel F_{254} plates (0.25 mm), respectively. The spots and bands were detected by UV irradiation (254 or 365 nm) or by staining with 3% *p*-anisaldehyde followed by heating. Melting points were measured using a Büchi B 545 apparatus and are uncorrected. ^{1}H, ^{13}C and ^{19}F nuclear magnetic resonance (NMR) spectra were recorded on a JEOL JMN-400 spectrometer operating at 400 MHz (^{1}H NMR), 100 MHz (^{13}C NMR) and 376 MHz (^{19}F NMR in $CDCl_3$ or DMSO-d_6 at 25 °C. The chemical shifts in ^{1}H NMR spectra were recorded relative to residual solvent peaks ($CDCl_3$: δ 7.26 or tetramethylsilane: δ 0.00 or DMSO-d_6: δ 2.50). The chemical shifts in ^{13}C NMR spectrum were recorded relative to residual solvent peaks ($CDCl_3$: δ 77.0 or DMSO-d_6: δ 39.5). The chemical shifts in ^{19}F NMR spectrum were recorded relative to residual solvent peaks (4-trifluoromethylbenzene: δ −63.7). The data are reported as follows: chemical shift in ppm (δ), multiplicity (s = singlet, d = doublet, t = triplet, q = quartet and m = multiplet), coupling constant (Hz), and integration. Infrared spectra (IR) were obtained using a Hitachi 270-50 spectrometer; absorptions are reported in reciprocal centimeters (cm^{-1}) for strong and structurally important peaks. High-resolution mass spectra (HRMS) were obtained using a Thermo Scientific Exactive Plus Orbitrap (Thermo Fisher Scientific, Inc., Waltham, MA, USA).

Materials: All commercially available reagents were used as received unless otherwise noted. (Diacetoxyiodo)arenes were synthesized from iodoarenes, 9% AcOOH in TFE or HFIP according to our procedure[S1] or $NaBO_3 \cdot 4H_2O$ in CH_3CN according to the reported procedure[S2]. Iodosylarenes were synthesized from (diacetoxyiodo)arenes or (dichloroiodo)arenes by treating with 3 N aqueous sodium hydroxide solution according to our reported procedure[S3].

2. General Procedure

A; The synthesis of phenyl(aryl)iodonium(III) acetates (3aa–ae) (Scheme 3)

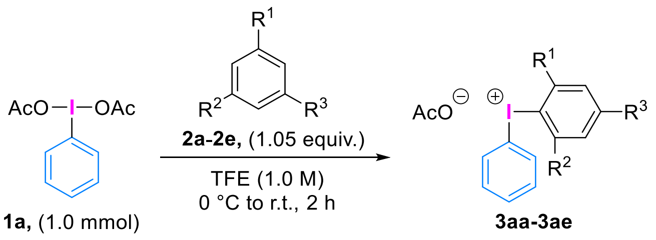

A solution of (diacetoxyiodo)benzene (**1a**, 1.0 mmol, 322.1 mg) in 2,2,2-trifluoroethanol (TFE, 1.0 mL, 1.0 M) was cooled to 0 °C and then the arene (**2**, 1.05 mmol, 1.05 equiv) was added to the reaction mixture. After stirring at room temperature for 2 h, the reaction mixture was concentrated under reduced pressure. ^{1}H NMR yield was calculated by using 1,1,2,2-tetrachloroethane as an internal standard. Addition of Et_2O and/or *n*-hexane to the residue generated a precipitant, which was collected by filtration to afford the corresponding phenyl(aryl)iodonium(III) acetate **3**.

B; The synthesis of aryl(TMP)iodonium(III) acetates (3ae, 4b–m) (Scheme 4)

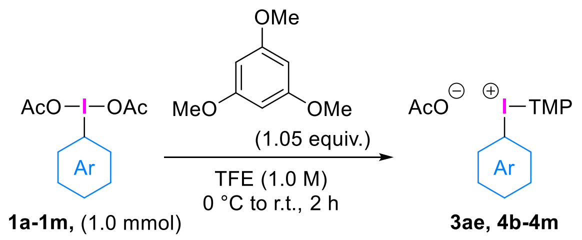

In a manner similar to our previous conditions[S1], a solution of (diacetoxyiodo)arene (**1**, 1.0 mmol) 2,2,2-trifluoroethanol (TFE, 1.0 mL, 1.0 M) was cooled at 0 °C and 1,3,5-trimethoxybenzene (TMP-H, 1.05 mmol, 1.05 equiv, 176.6 mg) was added to the reaction mixture. After stirring at room temperature for 2 h, the reaction mixture was concentrated under reduced pressure. Addition of Et_2O and/or *n*-hexane to the residue generated a precipitant, which was collected by filtration to afford the corresponding aryl(TMP)iodonium(III) acetate **4**.

C; The synthesis of aryl(TMP)iodonium(III) carboxylates (3ae, 7aa-7aj, 7ba–fa) (Scheme 5)

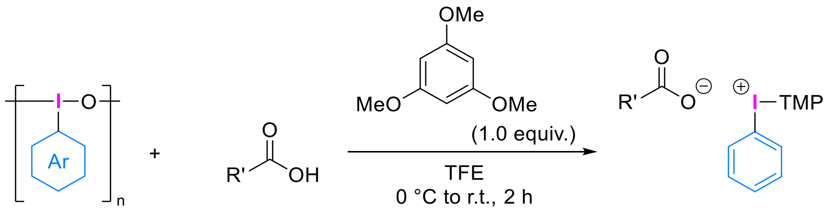

5a-5f, (1.0 mmol) **6a-6j,** (1.0 equiv.) **3ae, 7aa-7aj, 7ba-7fa**

In a manner similar to our previous conditions[S3], a solution of carboxylic acid (**6**, 1.0 mmol, 1.0 equiv) and 1,3,5-trimethoxybenzene (TMP-H, 1.0 mmol, 1.0 equiv, 168.2 mg) in 2,2,2-trifluoroethanol (TFE, 1.0 mL, 1.0 M) was cooled at 0 °C and iodosylarene (**5**, 1.0 mmol) was added to the reaction mixture in one portion. After stirring at room temperature for 2–4 h, the reaction mixture was concentrated under reduced pressure. Addition of Et_2O and/or *n*-hexane to the residue generated a precipitant, which was collected by filtration to afford the corresponding aryl(TMP)iodonium(III) carboxylate **7**.

Phenyl(2,4-dimethoxyphenyl)iodonium acetate (3ad)

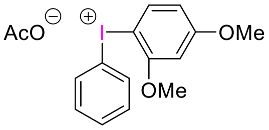

The title compound (**3ad**) was synthesized according to the general procedure A using (diacetoxyiodo)benzene (**1a**, 322.1 mg, 1.0 mmol) and 1,3-dimethoxybenzene (135.6 μL, 1.05 mmol) followed by trituration with Et_2O 15 mL. The product was obtained in 69% yield (276.5 mg, 0.69 mmol) as a white amorphous solid.

Melting Point: 143.7–144.3 °C.

^{1}H NMR (400 MHz, $CDCl_3$): δ 7.89 (d, J = 7.8 Hz, 2H), 7.63 (d, J = 7.8 Hz, 1H), 7.42 (t, J = 7.3 Hz, 1H), 7.30 (t, J = 7.8 Hz, 2H),6.44–6.42 (m, 2H), 3.79 (s, 3H), 3.78 (s, 3H), 1.86 (s, 3H) ppm.

^{13}C{^{1}H} NMR (100 MHz, $CDCl_3$): δ 178.6, 164.1, 158.8, 137.0, 133.9, 130.9, 130.5, 118.9, 107.6, 100.2, 99.4, 56.3, 55.6, 24.4 ppm.

IR (KBr): 3079, 3006, 2939, 2839, 1565, 1390, 1256, 1158, 1054 cm^{-1}.

HRMS-DART (m/z): ([M − OAc]$^+$) calcd for $C_{14}H_{14}IO_2^+$, 341.0033; found, 341.0030.

Phenyl(2,4,6-trimethoxyphenyl)iodonium acetate (3ae)

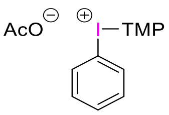

The title compound **3ae** was synthesized according to the general procedure B using (diacetoxyiodo)benzene (**1a**, 322.1 mg, 1.0 mmol) and 1,3,5-trimethoxybenzene (176.5 mg, 1.05 mmol) followed by trituration with Et_2O and n-hexane (1:1) 30 mL. The product was obtained in 96% yield (396.0 mg, 0.96 mmol) as a white amorphous solid. This compound (**3ae**) was also synthesized according to the general procedure C using iodosylbenzene (**5a**, 220.0 mg, 1.0 mmol), 1,3,5-trimethoxybenzene (168.6 mg, 1.0 mmol) and acetic acid (**6e**, 60.3 mg, 1.0 mmol) followed by trituration with Et_2O and n-hexane (1:1) 12 mL. The product was obtained in 81% yield (350.2 mg, 0.81 mmol) as a pale white amorphous solid.

Melting Point: 121.5–122.1 °C.

^{1}H NMR (400 MHz, $CDCl_3$): δ 7.92 (dd, J = 8.3, 1.0 Hz, 2H), 7.41 (t, J = 7.6 Hz, 1H), 7.29 (t, J = 7.8 Hz, 2H), 6.12 (s, 2H), 3.84 (s, 3H), 3.83 (s, 6H), 1.95 (s, 3H) ppm.

^{13}C{^{1}H} NMR (100 MHz, $CDCl_3$): δ 178.6, 165.6, 160.5, 133.6, 130.7, 130.2, 119.3, 91.1, 90.8, 56.3, 55.6, 24.5 ppm.

IR (KBr): 3051, 2996, 2839, 1583, 1410, 1338, 1229, 1208, 1122, 1069, 995, 744 cm^{-1}.

Spectral data of **3ae** are identical to the previously reported.[S1]

4-Methylphenyl(2,4,6-trimethoxyphenyl)iodonium acetate (4b)

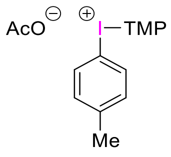

The title compound **4b** was synthesized according to the general procedure B using 4-methyl(diacetoxyiodo)benzene (**1b**, 336.1 mg, 1.0 mmol) and 1,3,5-trimethoxybenzene (176.7 mg, 1.05 mmol) followed by trituration with Et_2O and *n*-hexane (1:1) 30 mL. The product was obtained in 98% yield (435.7 mg, 0.98 mmol) as a pale pink amorphous solid.

Melting Point: 131.1–131.9 °C.

^{1}H NMR (400 MHz, $CDCl_3$): δ 7.75 (d, J = 8.3 Hz, 2H), 7.06 (d, J = 8.3 Hz, 2H), 6.09 (s, 2H), 3.82 (s, 6H), 3.80 (s, 3H), 2.29 (s, 3H), 1.91 (s, 3H) ppm.

^{13}C{^{1}H} NMR (100 MHz, $CDCl_3$): δ 177.3, 165.7, 160.4, 141.0, 133.8, 131.6, 115.0, 91.0, 89.7, 56.4, 55.6, 23.5, 21.1 ppm.

IR (KBr): 2976, 2939, 1582, 1411, 1341, 1228, 1208, 1123, 1066, 1002, 750 cm^{-1}.

Spectral data of **4b** are identical to the previously reported.[S1]

4-Methoxyphenyl(2,4,6-trimethoxyphenyl)iodonium acetate (4c)

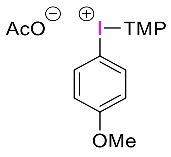

The title compound **4c** was synthesized according to the general procedure B using 4-methoxy(diacetoxyiodo)benzene (**1c**, 351.8 mg, 1.0 mmol) and 1,3,5-trimethoxybenzene (176.5 mg, 1.05 mmol) followed by trituration with Et_2O and *n*-hexane (1:1) 30 mL. The product was obtained in 96% yield (321.6 mg, 0.96 mmol) as a white amorphous solid.

Melting Point: 152.2–152.8 °C

^{1}H NMR (400 MHz, $CDCl_3$): δ 7.82 (d, J = 8.8 Hz, 2H), 6.79 (d, J = 8.8 Hz, 2H), 6.11 (s, 2H), 3.86 (s, 6H), 3.82 (s, 3H), 3.76 (s, 3H), 1.93 (s, 3H) ppm.

^{13}C{^{1}H} NMR (100 MHz, $CDCl_3$): δ 176.8, 166.0, 161.5, 160.4, 136.0, 116.7, 107.0, 91.1, 88.8,

56.6, 55.7, 55.4, 23.1 ppm.

IR (KBr): 3006, 2974, 1581, 1409, 1341, 1253, 1230, 1121, 1021, 822, 749 cm^{-1}.

Spectral data of **4c** are identical to the previously reported.[S1]

4,4'-Biphenyl(2,4,6-trimethoxyphenyl)iodonium acetate (4d)

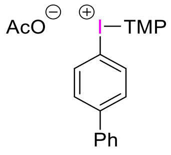

The title compound **4d** was synthesized according to the general procedure B using 4-phenyl(diacetoxyiodo)benzene (**1d**, 398.2 mg, 1.0 mmol) and 1,3,5-trimethoxybenzene (176.7 mg, 1.05 mmol) followed by trituration with Et_2O 15 mL. The product was obtained in 88% yield (447.6 mg, 0.88 mmol) as a pale pink amorphous solid.

Melting Point: 150.9–152.2 °C.

^{1}H NMR (400 MHz, $CDCl_3$): δ 7.98 (d, J = 8.8 Hz, 2H), 7.50–7.35 (m, 7H), 6.13 (s, 2H), 3.86 (s, 6H), 3.84 (s, 3H), 1.98 (s, 3H) ppm.

^{13}C{^{1}H} NMR (100 MHz, $CDCl_3$): δ 178.7, 165.6, 160.6, 143.4, 139.4, 134.2, 129.5, 128.9, 128.1, 127.1, 147.8, 91.1, 90.9, 56.4, 55.6, 24.5 ppm.

IR (KBr): 3061, 2952, 2843, 1696, 1583, 1341, 1233, 1128, 999, 864 cm^{-1}.

Spectral data of **4d** are identical to the previously reported.[S1]

4-Methoxycarbonylphenyl(2,4,6-trimethoxyphenyl)iodonium acetate (4e)

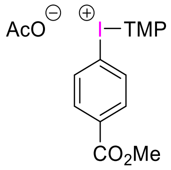

The title compound **4e** was synthesized according to the general procedure B using 4-methoxycarbonyl(diacetoxyiodo)benzene (**1e**, 380.1 mg, 1.0 mmol) and 1,3,5-trimethoxybenzene (176.8 mg, 1.05 mmol) followed by trituration with Et_2O 15 mL. The product was obtained in 87% yield (426.5 mg, 0.87 mmol) as a white amorphous solid.

Melting Point: 104.1–104.5 °C.

^{1}H NMR (400 MHz, $CDCl_3$): δ 7.98 (d, J = 8.8 Hz, 2H), 7.91 (d, J = 8.8 Hz, 2H), 6.12 (s, 2H), 3.90 (s, 3H), 3.84 (s, 3H), 3.83 (s, 6H), 1.95 (s, 3H) ppm.

^{13}C{^{1}H} NMR (100 MHz, $CDCl_3$): δ 178.5, 165.8, 165.7, 160.4, 133.6, 131.7, 131.5, 123.9, 91.0, 90.7, 56.4, 55.6, 52.4, 24.2 ppm.

IR (KBr): 3604, 2945, 2844, 1716, 1583, 1287, 1120, 1006, 810 cm^{-1}.

Spectral data of **4e** are identical to the previously reported.[S1]

4-Nitorophenyl(2,4,6-trimethoxyphenyl)iodonium acetate (4f)

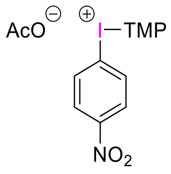

The title compound **4f** was synthesized according to the general procedure B using 4-nitro(diacetoxyiodo)benzene (**1f**, 367.4 mg, 1.0 mmol) and 1,3,5-trimethoxybenzene (176.8 mg, 1.05 mmol) followed by trituration with Et_2O 15 mL. The product was obtained in 90% yield (428.5 mg, 0.90 mmol) as a yellow amorphous solid.

Melting Point: 94.8–95.6 °C.

^{1}H NMR (400 MHz, $CDCl_3$): δ 8.09 (d, J = 9.3 Hz, 2H), 8.06 (d, J = 9.3 Hz, 2H), 6.14 (s, 2H), 3.85 (s, 6H), 3.83 (s, 3H), 1.91 (s, 3H) ppm.

^{13}C{^{1}H} NMR (100 MHz, $CDCl_3$): δ 176.9, 166.4, 160.4, 148.9, 134.8, 125.3, 124.8, 91.3, 89.7, 56.7, 55.8, 22.8 ppm.

IR (KBr): 3098, 2960, 1701, 1523, 1414, 1343, 1228, 1129, 1005, 849 cm^{-1}.

Spectral data of **4f** are identical to the previously reported.[S1]

2-Fluorophenyl(2,4,6-trimethoxyphenyl)iodonium acetate (4g)

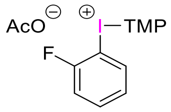

The title compound **4g** was synthesized according to the general procedure B using 2-fluoro(diacetoxyiodo)benzene (**1g**, 340.3 mg, 1.0 mmol) and 1,3,5-trimethoxybenzene (176.9 mg, 1.05 mmol) followed by trituration with Et_2O 15 mL. The product was obtained in 90% yield (401.6 mg, 0.90 mmol) as a white amorphous solid.

Melting Point: 163.9–164.5 °C.

^{1}H NMR (400 MHz, $CDCl_3$): δ 7.82 (ddd, J = 7.6, 5.8, 1.4 Hz, 1H), 7.37 (m, 1H), 7.10 (ddd, J = 8.3, 6.8, 1.4 Hz, 1H), 7.03 (ddd, J = 7.8, 6.4, 1.4 Hz, 1H), 6.09 (s, 2H), 3.84 (s, 6H), 3.79 (s, 3H), 1.86 (s, 3H) ppm.

^{13}C{^{1}H} NMR (100 MHz, $CDCl_3$): δ 178.6, 165.5, 160.2 (d, J = 249.5 Hz), 159.8, 136.2, 132.8 (d, J = 7.4 Hz), 125.9 (d, J = 2.5 Hz), 116.1 (d, J = 22.2 Hz), 107.1 (d, J = 24.7 Hz), 91.3, 91.1, 56.5,

55.6, 24.1 ppm.

^{19}F NMR (376 MHz, CDCl$_3$): δ −98.7 ppm.

IR (KBr): 3086, 2974, 2948, 1558, 1409, 1227, 1119, 1067, 817, 771 cm^{-1}.

Spectral data of **4g** are identical to the previously reported.[S1]

2,6-Dimethylphenyl(2,4,6-trimethoxyphenyl)iodonium acetate (4h)

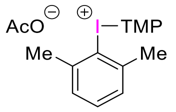

The title compound **4h** was synthesized according to the general procedure B using 2,6-dimethyl(diacetoxyiodo)benzene (**1h**, 350.1 mg, 1.0 mmol) and 1,3,5-trimethoxybenzene (176.4 mg, 1.05 mmol) followed by trituration with Et_2O 25 mL. The product was obtained in 83% yield (316.5 mg, 0.83 mmol) as a white amorphous solid.

Melting Point: 91.7–93.3 °C.

^{1}H NMR (400 MHz, CDCl$_3$): δ 7.20 (dd, J = 7.8 Hz, 6.8 Hz, 1H), 7.10 (d, J = 7.3 Hz, 2H), 6.07 (s, 2H), 3.80 (s, 3H), 3.76 (s, 6H), 2.67 (s, 6H), 1.88 (s, 3H) ppm.

^{13}C{^{1}H} NMR (100 MHz, CDCl$_3$): δ 176.8, 165.4, 160.5, 142.1, 131.2, 128.2, 127.6, 91.2, 86.8, 56.2, 55.6, 26.6, 23.0 ppm.

IR (KBr): 2952, 2844, 1718, 1583, 1340, 1123, 1016, 818 cm^{-1}.

Spectral data of **4h** are identical to the previously reported.[S1]

3-[[*N*-(1,1-dimethylethoxy)carbonyl]amino]phenyl(2,4,6-trimethoxyphenyl)iodonium acetate (4i)

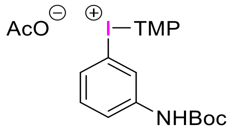

The title compound **4i** was synthesized according to the general procedure B using 3-[*N*-(1,1-dimethylethoxy)carbonyl]amino(diacetoxyiodo)benzene (**1i**, 218.5 mg, 0.50 mmol) and 1,3,5-trimethoxybenzene (88.3 mg, 0.53 mmol) followed by trituration with Et_2O 10 mL. The product was obtained in 85% yield (230.3 mg, 0.42 mmol) as an off-white amorphous solid.

Melting Point: 146.2–146.9 °C.

^{1}H NMR (400 MHz, CDCl$_3$): δ 9.68 (br s, 1H), 7.98 (s, 1H), 7.45 (d, J = 8.3 Hz, 1H), 7.40 (d, J =

8.3 Hz, 1H), 6.98 (t, J = 8.3 Hz, 1H), 6.09 (s, 2H), 3.87 (s, 6H), 3.81 (s, 3H), 1.92 (s, 3H), 1.50 (s, 9H) ppm.

$^{13}C\{^1H\}$ NMR (100 MHz, $CDCl_3$): δ 178.3, 165.6, 160.5, 152.9, 141.9, 130.0, 127.2, 124.1, 120.3, 118.1, 90.8, 89.9, 79.3, 56.5, 55.6, 28.4, 24.3 ppm.

IR (KBr): 3208, 3143, 2976, 1713, 1583, 1418, 1248, 1161, 1124, 1065, 1029, 990, 765 cm^{-1}.

HRMS-DART (m/z): ($[M - OAc]^+$) calcd for $C_{20}H_{25}INO_5^+$, 486.0772; found, 486.0768.

2-Chloropyridine-5-yl(2,4,6-trimethoxyphenyl)iodonium acetate (4j)

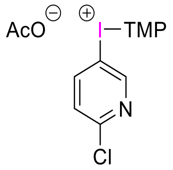

The title compound **4j** was synthesized according to the general procedure B using 2-chloro-5-(diacetoxyiodo)pyridine (**1j**, 357.4 mg, 1.0 mmol) and 1,3,5-trimethoxybenzene (176.9 mg, 1.05 mmol) followed by trituration with Et_2O 15 mL. The product was obtained in 91% yield (424.2 mg, 0.91 mmol) as a white amorphous solid.

Melting Point: 149.6–150.9 °C.

^1H NMR (400 MHz, $CDCl_3$): δ 8.65 (d, J = 2.4 Hz, 1H), 8.25 (dd, J = 8.6, 2.2 Hz, 1H), 7.24 (d, J = 8.8 Hz, 1H), 6.10 (s, 2H), 3.84 (s, 6H), 3.81 (s, 3H), 1.80 (s, 3H) ppm.

$^{13}C\{^1H\}$ NMR (100 MHz, $CDCl_3$): δ 178.6, 165.8, 160.1, 152.8, 152.7, 143.8, 126.4, 116.7, 92.0, 91.0, 56.5, 55.7, 24.1 ppm.

IR (KBr): 2939, 2837, 1550, 1339, 1224, 1101, 999, 807 cm^{-1}.

Spectral data of **4j** are identical to the previously reported.[S1]

3-Thienyl(2,4,6-trimethoxyphenyl)iodonium acetate (4k)

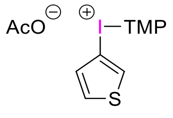

The title compound **4k** was synthesized according to the general procedure B using 3-(diacetoxyiodo)thiophene (**1k**, 327.8 mg, 1.0 mmol) and 1,3,5-trimethoxybenzene (176.4 mg, 1.05 mmol) followed by trituration with Et_2O 15 mL. The product was obtained in 93% yield (405.9 mg, 0.93 mmol) as a white amorphous solid.

Melting Point: 95.1–96.0 °C.

^1H NMR (400 MHz, $CDCl_3$): δ 7.90 (d, J = 2.2 Hz, 1H), 7.28 (d, J = 2.0 Hz, 2H), 6.15 (s, 2H), 3.88

(s, 6H), 3.85 (s, 3H), 1.95 (s, 3H) ppm.

$^{13}C\{^1H\}$ NMR (100 MHz, $CDCl_3$): δ 177.2, 166.0, 160.3, 131.6, 130.7, 127.7, 102.0, 91.1, 89.6, 56.6, 55.7, 23.2 ppm.

IR (KBr): 3119, 2962, 1581, 1410, 1339, 1229, 1162, 1120, 1065, 1021, 838, 774 cm^{-1}.

HRMS-DART (m/z): ($[M - OAc]^+$) calcd for $C_{13}H_{14}IO_3S^+$, 376.9703; found, 376.9700.

1,1'-Biphenyl-4,4'-diyl(2,4,6-trimethoxyphenyl)iodonium acetate (4l)

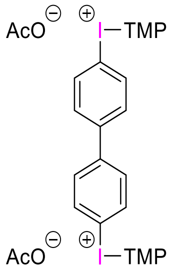

The title compound **4l** was synthesized according to the general procedure B using 4,4'-bis(diacetoxyiodo)biphenyl (**1l**, 641.9 mg, 1.0 mmol) and 1,3,5-trimethoxybenzene (352.9 mg, 2.1 mmol) followed by trituration with Et_2O 15 mL. The product was obtained in 88% yield (755.6 mg, 0.88 mmol) as a white amorphous solid.

Melting Point: 183.3–184.6 °C.

^1H NMR (400 MHz, $CDCl_3$): δ 7.94 (d, J = 8.8 Hz, 4H), 7.37 (d, J = 8.8 Hz, 4H), 6.13 (s, 4H), 3.85 (s, 12H), 3.84 (s, 6H), 1.94 (s, 6H) ppm.

$^{13}C\{^1H\}$ NMR (100 MHz, $CDCl_3$): δ 177.1, 166.0, 160.4, 141.7, 134.4, 129.4, 117.7, 91.1, 88.9, 56.5, 55.7, 23.2 ppm.

IR (KBr): 2950, 2845, 1583, 1387, 1161, 1125, 1066, 993, 806 cm^{-1}.

Spectral data of **4l** are identical to the previously reported.[S1]

Phenyl(2,4,6-trimethoxyphenyl)iodonium trifluoroacetate (4m)

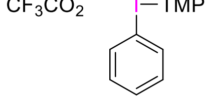

The title compound **4m** was synthesized according to the general procedure B using bis(trifluoroacetoxyiodo)benzene (**1m**, 430.9 mg, 1.0 mmol) and 1,3,5-trimethoxybenzene (176.6 mg, 1.05 mmol) followed by trituration with Et_2O 10 mL. The product was obtained in 91% yield (442.7 mg, 0.91 mmol) as a pale yellow amorphous solid.

Melting Point: 164.4–165.4 °C.

^1H NMR (400 MHz, DMSO-d_6): δ 7.94 (d, J = 7.8 Hz, 2H), 7.56 (t, J = 7.3 Hz, 1H), 7.45 (t, J = 7.8

Hz, 2H), 6.47 (s, 2H), 3.95 (s, 6H), 3.87 (s, 3H) ppm.

^{13}C{^{1}H} NMR (100 MHz, DMSO-d_6): δ 166.2, 159.6, 158.2 (q, J = 31.2 Hz), 134.2, 131.4, 131.3, 117.3 (q, J = 300.1 Hz), 116.7, 92.1, 87.7, 57.3, 56.1 ppm.

^{19}F NMR (376 MHz, DMSO-d_6): δ −75.7 ppm.

IR (KBr): 3078, 2995, 2953, 1680, 1586, 1467, 1345, 1230, 990, 752 cm^{-1}.

Spectral data of **4m** are identical to the previously reported.[S4]

Phenyl(2,4,6-trimethoxyphenyl)iodonium benzoate (7aa)

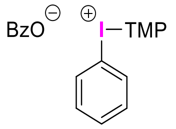

The title compound **7aa** was synthesized according to the general procedure C using iodosylbenzene (**5a**, 219.8 mg, 1.0 mmol), 1,3,5-trimethoxybenzene (168.2 mg, 1.0 mmol) and benzoic acid (**6a**, 122.5 mg, 1.0 mmol) followed by trituration with Et_2O 15 mL. The product was obtained in 80% yield (392.1 mg, 0.80 mmol) as a white amorphous solid.

Melting Point: 144.2–144.9 °C.

^{1}H NMR (400 MHz, CDCl$_3$): δ 8.03–8.00 (m, 4H), 7.41 (t, J = 7.3 Hz, 1H), 7.34–7.28 (m, 5H), 6.13 (s, 2H), 3.84 (s, 3H), 3.83 (s, 6H) ppm.

^{13}C{^{1}H} NMR (100 MHz, CDCl$_3$): δ 173.0, 165.8, 160.7, 137.8, 133.6, 130.7, 130.2, 129.6, 129.4, 127.4, 119.4, 90.9, 90.5, 56.4, 55.7 ppm.

IR (KBr): 3062, 3017, 2936, 2839, 1579, 1461, 1336, 1226, 1124, 990, 715 cm^{-1}.

Spectral data of **7aa** are identical to the previously reported.[S3]

Phenyl(2,4,6-trimethoxyphenyl)iodonium 2-iodobenzoate (7ab)

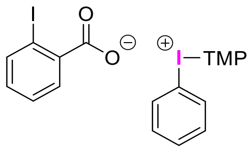

The title compound **7ab** was synthesized according to the general procedure C using iodosylbenzene (**5a**, 220.0 mg, 1.0 mmol), 1,3,5-trimethoxybenzene (168.8 mg, 1.0 mmol) and 2-iodobenzoic acid (**6b**, 248.5 mg, 1.0 mmol) followed by trituration with Et_2O 15 mL. The product was obtained in 78% yield (480.7 mg, 0.78 mmol) as a white amorphous solid.

Melting Point: 132.3–133.1 °C.

^{1}H NMR (400 MHz, CDCl$_3$): δ 8.01 (d, J = 8.3 Hz, 2H), 7.77 (d, J = 7.8 Hz, 1H), 7.53 (d, J = 7.8 Hz, 1H), 7.42 (t, J = 7.3 Hz, 1H), 7.30 (t, J = 7.8 Hz, 2H), 7.23 (t, J = 7.6 Hz, 1H), 6.88 (td, J = 7.7, 1.5 Hz, 1H), 6.14 (s, 2H), 3.85 (s, 9H) ppm.

^{13}C{^{1}H} NMR (100 MHz, CDCl$_3$): δ 174.2, 165.9, 160.7, 145.3, 139.3, 133.8, 130.8, 130.4, 128.8, 127.4, 119.0, 93.2, 91.0, 89.6, 56.5, 55.7 ppm.

IR (KBr): 3056, 2938, 2836, 1584, 1463, 1407, 1224, 1204, 1123, 1069, 1011, 994 cm^{-1}.

HRMS-DART (m/z): ([M – 2-iodobenzoate]$^{+}$) calcd for $C_{15}H_{16}IO_3^{+}$, 371.0139; found, 371.0137.

Phenyl(2,4,6-trimethoxyphenyl)iodonium pyridine-3-carboxylate (7ac)

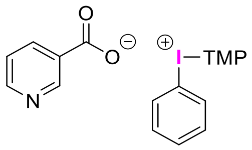

The title compound **7ac** was synthesized according to the general procedure C using iodosylbenzene (**5a**, 220.1 mg, 1.0 mmol), 1,3,5-trimethoxybenzene (168.5 mg, 1.0 mmol) and pyridine-3-carboxylic acid (**6c**, 123.7 mg, 1.0 mmol) followed by trituration with Et_2O 10 mL. The product was obtained in 78% yield (385.6 mg, 0.78 mmol) as a white amorphous solid.

Melting Point: 135.5–136.3 °C.

^{1}H NMR (400 MHz, CDCl$_3$): δ 9.14 (d, J = 2.0 Hz, 1H), 8.54 (dd, J = 4.9, 1.5 Hz, 1H), 8.24 (dt, J = 7.8, 1.7 Hz, 1H), 7.98 (d, J = 7.8 Hz, 2H), 7.43 (t, J = 7.3 Hz, 1H), 7.30 (t, J = 7.8 Hz, 2H), 7.23 (dd, J = 7.8, 4.9 Hz, 1H), 6.13 (s, 2H), 3.84 (s, 9H) ppm.

^{13}C{^{1}H} NMR (100 MHz, CDCl$_3$): δ 170.7, 165.9, 160.5, 151.1, 150.3, 136.9, 133.7, 132.6, 130.8, 130.5, 122.5, 118.8, 91.0, 89.3, 56.5, 55.7 ppm.

IR (KBr): 3066, 3020, 2970, 2944, 2844, 1605, 1581, 1469, 1407, 1231, 1211, 1124, 1066, 1022, 753 cm^{-1}.

HRMS-DART (m/z): ([M – pyridine-3-carboxylate]$^{+}$) calcd for $C_{15}H_{16}IO_3^{+}$, 371.0139; found, 371.0137.

Phenyl(2,4,6-trimethoxyphenyl)iodonium thiophene-3-carboxylate (7ad)

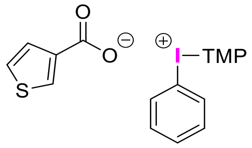

The title compound **7ad** was synthesized according to the general procedure C using iodosylbenzene (**5a**, 220.0 mg, 1.0 mmol), 1,3,5-trimethoxybenzene (168.7 mg, 1.0 mmol) and thiophene-3-carboxylic acid (**6d**, 128.8 mg, 1.0 mmol) followed by trituration with Et_2O 10 mL. The product was obtained in 81% yield (404.1 mg, 0.81 mmol) as a white amorphous solid.

Melting Point: 133.1–133.8 °C.

^{1}H NMR (400 MHz, $CDCl_3$): δ 7.98 (d, J = 7.8 Hz, 2H), 7.81–7.80 (m, 1H), 7.45 (d, J = 4.9 Hz, 1H), 7.41 (t, J = 7.3 Hz, 1H), 7.28 (t, J = 7.8 Hz, 2H), 7.12–7.10 (m, 1H), 6.12 (s, 2H), 3.83 (s, 3H), 3.82 (s, 6H) ppm.

^{13}C{^{1}H} NMR (100 MHz, $CDCl_3$): δ 169.4, 165.8, 160.6, 142.0, 133.7, 130.7, 130.3, 129.1, 128.6, 123.7, 119.1, 90.9, 89.9, 56.4, 55.6 ppm.

IR (KBr): 3075, 3011, 2938, 2839, 1580, 1407, 1229, 1125, 1065, 990, 761 cm^{-1}.

HRMS-DART (m/z): ([M – thiophene-3-carbocylate]$^+$) calcd for $C_{15}H_{16}IO_3^+$, 371.0139; found, 371.0136.

Phenyl(2,4,6-trimethoxyphenyl)iodonium 2,2-dimethylpropanoate (7af)

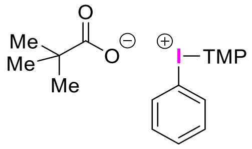

The title compound **7af** was synthesized according to the general procedure C using iodosylbenzene (**5a**, 219.9 mg, 1.0 mmol), 1,3,5-trimethoxybenzene (168.3 mg, 1.0 mmol) and 2,2-dimethylpropanoic acid (**6f**, 102.4 mg, 1.0 mmol) followed by trituration at 0 °C with Et_2O 2 mL. The product was obtained in 63% yield (298.3 mg, 0.63 mmol) as a white amorphous solid.

Melting Point: 142.0–142.9 °C.

^{1}H NMR (400 MHz, $CDCl_3$): δ 7.94 (d, J = 7.3 Hz, 2H), 7.38 (t, J = 7.3 Hz, 1H), 7.25 (t, J = 7.8 Hz, 2H), 6.11 (s, 2H), 3.83 (s, 3H), 3.81 (s, 6H), 1.11 (s, 9H) ppm.

^{13}C{^{1}H} NMR (100 MHz, $CDCl_3$): δ 185.2, 165.5, 160.7, 133.4, 130.4, 130.0, 120.2, 91.8, 90.8, 56.3, 55.6, 39.3, 28.4 ppm.

IR (KBr): 2978, 2943, 2862, 1555, 1413, 1343, 1229, 1126, 1067, 1027, 997, 823, 729 cm^{-1}.

HRMS-DART (m/z): ([M – 2,2-dimethylpropanoate]$^+$) calcd for $C_{15}H_{16}IO_3^+$, 371.0139; found, 371.0135.

Phenyl(2,4,6-trimethoxyphenyl)iodonium cyclohexanecarboxylate (7ag)

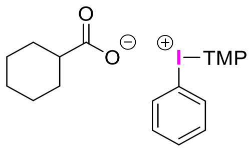

The title compound **7ag** was synthesized according to the general procedure C using iodosylbenzene (**5a**, 219.5 mg, 1.0 mmol), 1,3,5-trimethoxybenzene (168.8 mg, 1.0 mmol) and cyclohexanecarboxylic acid (**6g**, 130.1 mg, 1.0 mmol) followed by trituration with Et_2O and *n*-hexane (1:5) 12 mL. The product was obtained in 68% yield (337.0 mg, 0.68 mmol) as a white amorphous solid.

Melting Point: 135.7–136.4 °C.

^{1}H NMR (400 MHz, CDCl$_3$): δ 7.93 (d, J = 8.3 Hz, 2H), 7.39 (t, J = 7.3 Hz, 1H), 7.26 (t, J = 7.6 Hz, 2H), 6.11 (s, 2H), 3.83 (s, 3H), 3.81 (s, 6H) 2.08 (tt, J = 11.5, 3.5 Hz, 1H), 1.90–1.87 (m, 2H), 1.70–1.67 (m, 2H), 1.59–1.57 (m, 1H), 1.43–1.34 (m, 2H), 1.27–1.16 (m, 3H) ppm.

^{13}C{^{1}H} NMR (100 MHz, CDCl$_3$): δ 183.4, 165.5, 160.7, 133.6, 130.5, 130.1, 120.0, 91.8, 90.8, 56.3, 55.6, 46.6, 30.4, 26.3, 26.2 ppm.

IR (KBr): 3051, 2929, 2846, 1580, 1540, 1412, 1389, 1341, 1231, 1123, 1068, 1027, 948 cm^{-1}.

HRMS-DART (m/z): ([M – cyclohexanecarboxylate]$^+$) calcd for $C_{15}H_{16}IO_3^+$, 371.0139; found, 371.0136.

Phenyl(2,4,6-trimethoxyphenyl)iodonium 2-cyanoacetate (7ah)

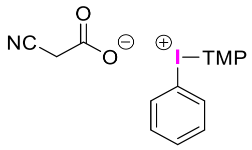

The title compound **7ah** was synthesized according to the general procedure C using iodosylbenzene (**5a**, 219.5 mg, 1.0 mmol), 1,3,5-trimethoxybenzene (168.5 mg, 1.0 mmol) and 2-cyanoacetic acid (**6h**, 85.6 mg, 1.0 mmol) followed by trituration with Et_2O 6 mL. The product was obtained in 78% yield (354.0 mg, 0.78 mmol) as a pale white amorphous solid.

Melting Point: 135.4–135.9 °C.

^{1}H NMR (400 MHz, CDCl$_3$): δ 7.91 (d, J = 7.3 Hz, 2H), 7.46 (t, J = 7.3 Hz, 1H), 7.33 (t, J = 7.8 Hz, 2H), 6.16 (s, 2H), 3.87 (s, 6H), 3.86 (s, 3H), 3.20 (s, 2H) ppm.

^{13}C{^{1}H} NMR (100 MHz, CDCl$_3$): δ 167.1, 166.3, 160.5, 133.6, 131.0, 130.8, 117.8, 117.4, 91.2, 87.4, 56.6, 55.8, 27.0 ppm.

IR (KBr): 2972, 2942, 2246, 1614, 1589, 1414, 1335, 1220, 1126, 1070, 993, 886, 806, 742 cm^{-1}.

HRMS-DART (m/z): ([M – 2-cyanoacetate]$^+$) calcd for $C_{15}H_{16}IO_3^+$, 371.0139; found, 371.0135.

Phenyl(2,4,6-trimethoxyphenyl)iodonium 2-(1,3-dimethyl-2,6-dioxo-1,2,3,6-tetrahydro-*7H*-purin-7-yl)acetate (7ai)

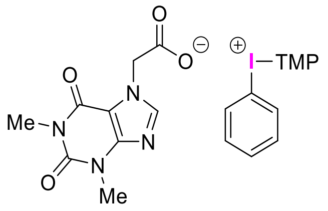

The title compound **7ai** was synthesized according to the general procedure C using iodosylbenzene (**5a**, 219.6 mg, 1.0 mmol), 1,3,5-trimethoxybenzene (168.4 mg, 1.0 mmol) and 2-(1,3-dimethyl-2,6-dioxo-1,2,3,6-tetrahydro-*7H*-purin-7-yl)acetic acid (**6i**, 238.6 mg, 1.0 mmol) followed by trituration with Et_2O 6 mL. The product was obtained in 86% yield (521.6 mg, 0.86 mmol) as a white amorphous solid.

Melting Point: 141.1–141.7 °C.

^{1}H NMR (400 MHz, CDCl$_3$): δ 7.86 (d, J = 7.3 Hz, 2H), 7.58 (s, 1H),7.44 (t, J = 7.6 Hz, 1H), 7.29 (t, J = 7.8 Hz, 2H), 6.13 (s, 2H), 4.91 (s, 2H), 3.85 (s, 9H), 3.58 (s, 3H), 3.34 (s, 3H) ppm.

^{13}C{^{1}H} NMR (100 MHz, CDCl$_3$): δ 171.3, 166.2, 160.5, 155.2, 151.8, 148.1, 142.4, 133.7, 131.0, 130.7, 117.7, 107.3, 91.1, 87.5, 55.6, 55.7, 49.3, 29.6, 27.8 ppm.

IR (KBr): 2952, 1701, 1668, 1603, 1549, 1470, 1339, 1231, 1162, 1124, 1064, 1028, 739 cm^{-1}.

HRMS-DART (m/z): ([M – 2-(1,3-dimethyl-2,6-dioxo-1,2,3,6-tetrahydro-*7H*-purin-7-yl)acetate]$^+$) calcd for $C_{15}H_{16}IO_3^+$, 371.0139; found, 371.0135.

Phenyl(2,4,6-trimethoxyphenyl)iodonium 7-methoxycoumarin-3-carboxylate (7aj)

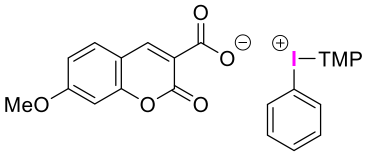

The title compound **7aj** was synthesized according to the general procedure C using iodosylbenzene (**5a**, 219.9 mg, 1.0 mmol), 1,3,5-trimethoxybenzene (168.6 mg, 1.0 mmol) and 7-methoxycoumarin-3-carboxylic acid (**6j**, 220.2 mg, 1.0 mmol) followed by trituration with Et_2O 15 mL. The product was obtained in 93% yield (549.8 mg, 0.93 mmol) as a white amorphous solid.

Melting Point: 178.9–179.5 °C.

^{1}H NMR (400 MHz, $CDCl_3$): δ 8.21 (s, 1H), 8.02 (d, J = 8.3 Hz, 2H), 7.41 (t, J = 7.3 Hz, 1H), 7.36 (d, J = 8.3 Hz, 1H), 7.30 (t, J = 7.8 Hz, 2H), 6.80–6.76 (m, 2H), 6.14 (s, 2H), 3.85–3.85 (m, 12H (trimethoxyphenyl 6H, 3H, coumarin 3H)) ppm.

^{13}C{^{1}H} NMR (100 MHz, $CDCl_3$): δ 169.0, 166.0, 162.7, 160.7, 159.6, 156.2, 143.6, 133.7, 130.9, 130.4, 129.4, 122.5, 118.6, 112.9, 112.2, 100.1, 91.0, 88.7, 56.5, 55.7, 55.6 ppm.

IR (KBr): 3063, 2992, 2951, 2838, 1733, 1617, 1577, 1373, 1235, 1011, 819, 738 cm^{-1}.

HRMS-DART (m/z): ([M – 7-methoxycoumarin-3-carboxylate]$^+$) calcd for $C_{15}H_{16}IO_3^+$, 371.0139; found, 371.0136.

4-Methylphenyl(2,4,6-trimethoxyphenyl)iodonium benzoate (7ba)

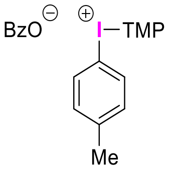

The title compound **7ba** was synthesized according to the general procedure C using 1-iodosyl-4-methylbenzene (**5b**, 233.9 mg, 1.0 mmol), 1,3,5-trimethoxybenzene (168.4 mg, 1.0 mmol) and benzoic acid (**6a**, 122.4 mg, 1.0 mmol) followed by trituration with Et_2O 10 mL. The product was obtained in 63% yield (320.6 mg, 0.63 mmol) as a white amorphous solid.

Melting Point: 161.7–162.4 °C.

^{1}H NMR (400 MHz, $CDCl_3$): δ 8.02 (dd, J = 8.0, 1.7 Hz, 2H), 7.88 (d, J = 8.5 Hz, 2H), 7.34–7.28 (m, 3H), 7.09 (d, J = 8.5 Hz, 2H), 6.12 (s, 2H), 3.83 (s, 9H), 2.32 (s, 3H) ppm.

^{13}C{^{1}H} NMR (100 MHz, $CDCl_3$): δ 173.0, 165.7, 160.7, 140.8, 137.8, 133.7, 131.5, 129.5, 129.4, 127.4, 115.9, 90.9, 90.5, 56.5, 55.7, 21.2 ppm.

IR (KBr): 2997, 2936, 1596, 1556, 1400, 1228, 1128, 1066, 718 cm^{-1}.

HRMS-DART (m/z): ([M − OBz]$^+$) calcd for $C_{16}H_{18}IO_3^+$, 385.0295; found, 385.0292.

4-Methoxylphenyl(2,4,6-trimethoxyphenyl)iodonium benzoate (7ca)

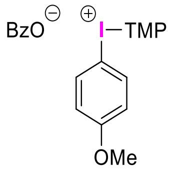

The title compound **7ca** was synthesized according to the general procedure C using 1-iodosyl-4-methoxybenzene (**5c**, 249.8 mg, 1.0 mmol), 1,3,5-trimethoxybenzene (168.4 mg, 1.0 mmol) and benzoic acid (**6a**, 122.2 mg, 1.0 mmol) followed by trituration with Et_2O 10 mL. The product was obtained in 86% yield (446.2 mg, 0.86 mmol) as a white amorphous solid.

Melting Point: 178.8–179.6 °C.

^{1}H NMR (400 MHz, CDCl$_3$): δ 8.01 (dd, J = 7.6, 1.7 Hz, 2H), 7.92 (d, J = 9.3 Hz, 2H), 7.36–7.28 (m, 3H), 6.80 (d, J = 9.3 Hz, 2H), 6.12 (s, 2H), 3.84 (s, 6H), 3.83 (s, 3H), 3.77 (s, 3H) ppm.

^{13}C{^{1}H} NMR (100 MHz, CDCl$_3$): δ 172.9, 165.6, 161.1, 160.6, 137,9, 135.5, 129.5, 129.4, 127.3, 116.4, 108.7, 90.9, 90.6, 56.5, 55.7, 55.4 ppm.

IR (KBr): 3001, 2942, 2841, 1588, 1550, 1255, 1119, 1069, 1017 cm^{-1}.

HRMS-DART (m/z): ([M − OBz]$^+$) calcd for $C_{16}H_{18}IO_4^+$, 401.0244; found, 401.0241.

4-Fluorophenyl(2,4,6-trimethoxyphenyl)iodonium benzoate (7da)

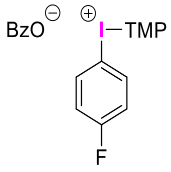

The title compound **7da** was synthesized according to the general procedure C using 1-iodosyl-4-fluorobenzene (**5d**, 237.8 mg, 1.0 mmol), 1,3,5-trimethoxybenzene (168.4 mg, 1.0 mmol) and benzoic acid (**6a**, 122.4 mg, 1.0 mmol) followed by trituration with Et_2O 6 mL. The product was obtained in 86% yield (439.7 mg, 0.86 mmol) as a white amorphous solid.

Melting Point: 149.8–150.4 °C.

^{1}H NMR (400 MHz, CDCl$_3$): δ 8.02–7.96 (m, 4H), 7.36–7.28 (m, 3H), 6.98 (t, J = 8.5 Hz, 2H), 6.11 (s, 2H), 3.83 (s, 9H) ppm.

^{13}C{^{1}H} NMR (100 MHz, CDCl$_3$): δ 172.9, 165.8, 163.6 (d, J = 251.6 Hz), 160.5, 137.6, 135.9 (d,

J = 8.3 Hz), 129.6, 129.3, 127.3, 118.0 (d, J = 22.3 Hz), 113.0 (d, J = 3.3 Hz), 90.9, 90.9, 56.4, 55.6 ppm.

^{19}F NMR (376 MHz, CDCl$_3$): δ −110.1 ppm.

IR (KBr): 3058, 3013, 2948, 2844, 1586, 1557, 1468, 1367, 1341, 1228, 1161, 1130, 1065, 1031, 809 cm^{-1}.

HRMS-DART (m/z): ([M − OBz]$^+$) calcd for $C_{15}H_{15}FIO_3^+$, 389.0044; found, 389.0041.

4-Bromophenyl(2,4,6-trimethoxyphenyl)iodonium benzoate (7ea)

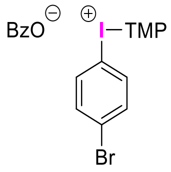

The title compound **7ea** was synthesized according to the general procedure C using 1-iodosyl-4-bromobenzene (**5e**, 298.9 mg, 1.0 mmol), 1,3,5-trimethoxybenzene (168.7 mg, 1.0 mmol) and benzoic acid (**6a**, 122.3 mg, 1.0 mmol) followed by trituration with Et_2O 10 mL. The product was obtained in 93% yield (528.7 mg, 0.93 mmol) as a white amorphous solid.

Melting Point: 158.5–159.1 °C.

^{1}H NMR (400 MHz, CDCl$_3$): δ 7.98 (dd, J = 7.8, 1.5 Hz, 2H), 7.87 (d, J = 8.8 Hz, 2H), 7.40 (d, J = 8.8 Hz, 2H), 7.37–7.28 (m, 3H), 6.12 (s, 2H), 3.84 (s, 3H), 3.83 (s, 6H) ppm.

^{13}C{^{1}H} NMR (100 MHz, CDCl$_3$): δ 173.0, 165.9, 160.6, 137.5, 135.2, 133.7, 129.7, 129.3, 127.4, 125.1, 117.7, 91.0, 56.5, 55.7 ppm.

IR (KBr): 3047, 2943, 2839, 1596, 1556, 1468, 1366, 1230, 1125, 1027, 996, 806 cm^{-1}.

HRMS-DART (m/z): ([M − OBz]$^+$) calcd for $C_{15}H_{15}BrIO_3^+$, 448.9244; found, 448.9241.

2,4,6-Trimethylphenyl(2,4,6-trimethoxyphenyl)iodonium benzoate (7fa)

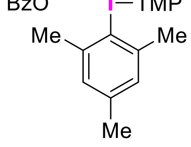

The title compound **7fa** was synthesized according to the general procedure C using 2-iodosyl-1,3,5-trimethylbenzene (**5f**, 262.2 mg, 1.0 mmol), 1,3,5-trimethoxybenzene (168.8 mg, 1.0 mmol) and benzoic acid (**6a**, 122.3 mg, 1.0 mmol) followed by trituration with Et_2O 4 mL. The product was obtained in 73% yield (389.6 mg,

0.73 mmol) as a white amorphous solid.

Melting Point: 143.5–144.3 °C.

^{1}H NMR (400 MHz, $CDCl_3$): δ 7.94 (d, J = 6.8 Hz, 2H), 7.31–7.25 (m, 3H), 6.92 (s, 2H), 6.08 (s, 2H), 3.80 (s, 3H), 3.77 (s, 6H), 2.67 (s, 6H), 2.25 (s, 3H) ppm.

^{13}C{^{1}H} NMR (100 MHz, $CDCl_3$): δ 173.0, 165.0, 160.6, 141.7, 141.2, 138.0, 129.4, 129.3, 128.9, 127.2, 125.1, 91.0, 88.4, 56.1, 55.6, 26.5, 20.8 ppm.

IR (KBr): 2976, 2943, 2842, 1583, 1556, 1457, 1362, 1337, 1234, 1127, 1068, 1032, 946 cm^{-1}.

HRMS-DART (m/z): ($[M - OBz]^{+}$) calcd for $C_{18}H_{22}IO_3^{+}$, 413.0608; found, 413.0605.

3. Arylation of various nucleophiles

A; Intramolecular arylation of phenyl(TMP)iodonium(III) benzoates (7aa) (Scheme 6A)

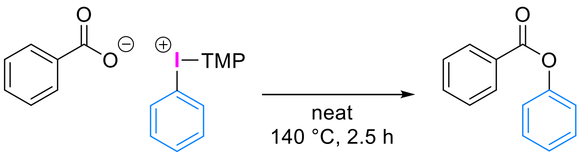

Stirring phenyl(TMP)iodonium(III) benzoate (**7aa**, 0.20 mmol, 98.3 mg) in a screw-capped test tube at 140 °C for 2.5 h. The reaction mixture was cooled to room temperature, and purified by flash column chromatography (SiO_2, dichloromethane / hexane = 33:67) to afford phenyl benzoate (**8aa**) in 70% yield (27.9 mg, 0.14 mmol) as a white amorphous solid.

Phenyl benzoate (8aa)

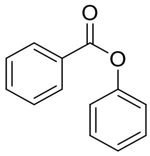

Melting Point: 64.8–65.6 °C.

^{1}H NMR (400 MHz, CDCl$_3$): δ 8.21 (d, J = 7.3 Hz, 2H), 7.64 (tt, J = 7.3, 1.5 Hz, 1H), 7.52 (t, J = 7.8 Hz, 2H), 7.44 (t, J = 7.8 Hz, 2H), 7.28 (t, J = 7.3 Hz, 1H), 7.23–7.21 (m, 2H) ppm.

^{13}C{^{1}H} NMR (100 MHz, CDCl$_3$): δ 165.2, 150.9, 133.6, 130.2, 129.5, 129.5, 128.6, 125.9, 121.7 ppm.

IR (KBr): 3066, 2926, 2855, 1730, 1596, 1489, 1451, 1262, 1199, 1001, 917, 694 cm^{-1}.

Spectral data of **8aa** are identical to the previously reported.[S5]

B; Phenol *O*-arylation with phenyl(2,4,6-trimethoxyphenyl)iodonium 7-methoxycoumarin-3-carboxylate (7aj) (Scheme 6B)

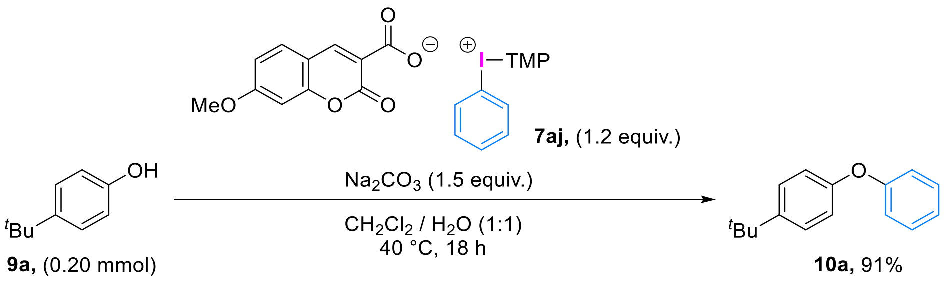

To a stirred solution of 4-*tert*-butylphenol (**9a**, 0.20 mmol, 29.7 mg) and Na_2CO_3 (0.30 mmol, 1.5 equiv, 31.8 mg) in dichloroethane (2.0 mL, 0.10 M) and water (2.0 mL, 0.10 M) in a screw-capped test tube, was added phenyl(TMP)iodonium(III) 7-methoxycoumarin-3-carboxylate (**7aj**, 0.22 mmol, 1.1 equiv, 129.9 mg), and stirring at 40 °C for 18 h. The reaction mixture was cooled to room temperature, and extracted with dichloromethane (3 × 10 mL). The combined organic fractions were dried over Na_2SO_4, and all volatiles were removed under vacuum. The residue was purified by flash column chromatography (SiO_2, AcOEt / hexane = 0:100) to afford 1-*tert*-butyl-4-phenoxybenzene (**10a**) in 91% yield (40.6 mg, 0.18 mmol) as a colorless oil.

1-*tert*-Butyl-4-phenoxybenzene (10a)

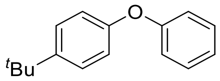

^{1}H NMR (400 MHz, $CDCl_3$): δ 7.35–7.29 (m, 4H), 7.07 (t, J = 7.6 Hz, 1H), 7.00 (d, J = 7.3 Hz, 2H), 6.94 (d, J = 8.8 Hz, 2H), 1.32 (s, 9H) ppm.

$^{13}C\{^1H\}$ NMR (100 MHz, $CDCl_3$): δ 157.6, 154.7, 146.1, 129.6, 126.5, 122.9, 118.6, 118.4, 34.3, 31.5 ppm.

IR (KBr): 296, 2902, 2867, 1590, 1508, 1489, 1239, 753, 691 cm^{-1}.

Spectral data of **10a** are identical to the previously reported.[S6]

C; *N*-Methoxysulfoneamide *N*-arylation with phenyl(2,4,6-trimethoxyphenyl)iodonium 7-methoxycoumarin-3-carboxylate (7aj) (Scheme 6C)

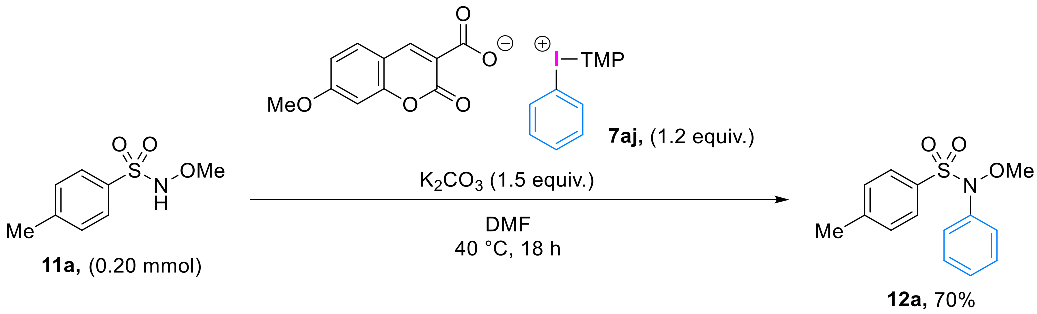

To a stirred solution of *N*-methoxy4-methylbenzenesulfonamide (**11a**, 0.20 mmol, 40.1 mg) in DMF (1.0 mL, 0.20 M) in a screw-capped test tube, were added phenyl(TMP)iodonium(III) 7-methoxycoumarin-3-carboxylate (**7aj**, 0.22 mmol, 1.1 equiv, 129.9 mg) and K_2CO_3 (0.30 mmol, 1.5 equiv, 41.3 mg). After sealing, the mixture was stirred at 40 °C for 18 h. The reaction mixture was cooled to room temperature and quenched with water. The organic residue was extracted with ethyl acetate (3 × 10 mL). The combined organic fractions were collected, dried over Na_2SO_4, and all volatiles were removed under vacuum. The residue was purified by flash column chromatography (SiO_2, AcOEt / hexane = 9:91) to afford *N*-methoxy-4-methyl-*N*-phenylbenzenesulfonamide (**12a**) in 70% yield (38.7 mg, 0.14 mmol) as a colorless oil.

***N*-Methoxy-4-methyl-*N*-phenylbenzenesulfonamide (12a)**

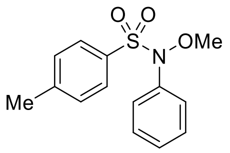

^{1}H NMR (400 MHz, $CDCl_3$): δ 7.40 (d, J = 8.3 Hz, 2 H), 7.27–7.22 (m, 3 H), 7.20 (d, J = 8.3 Hz, 2 H), 7.12–7.10 (m, 2 H), 3.88 (s, 3 H), 2.40 (s, 3 H) ppm.

^{13}C{^{1}H} NMR (100 MHz, $CDCl_3$): δ 144.7, 140.9, 130.1, 129.6, 129.0, 128.3, 127.5, 123.5, 64.2, 21.7 ppm.

IR (KBr): 2938, 1597, 1487, 1362, 1173, 1091, 1021, 696 cm^{-1}.

Spectral data of **12a** are identical to the previously reported.[S7]

4. Counterion exchange reaction

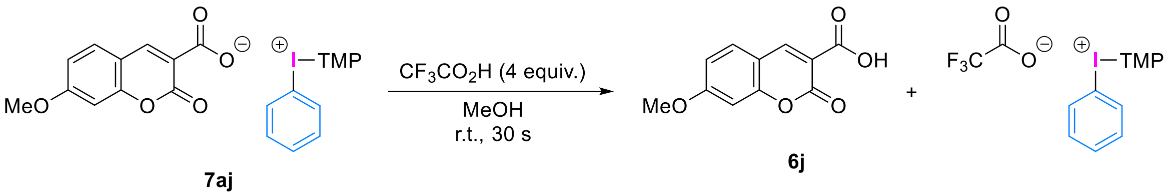

To a stirred solution of phenyl(TMP)iodonium(III) 7-methoxycoumarin-3-carboxylate (**7aj**, 0.025 mmol, 14.7 mg) in methanol (2.0 mL, 0.013 M) was added trifluoroacetic acid (0.10 mmol, 4.0 equiv, 7.7 μL) dropwise and the reaction mixture was stirred at room temperature. After 30 seconds, the resulting solution was irradiated by 365 nm UV light (Figure S1, right), and blue fluorescence emitted, derived from 7-methoxycoumarin-3-carboxylic acid (**6j**), was clearly observed (see Figure S1).

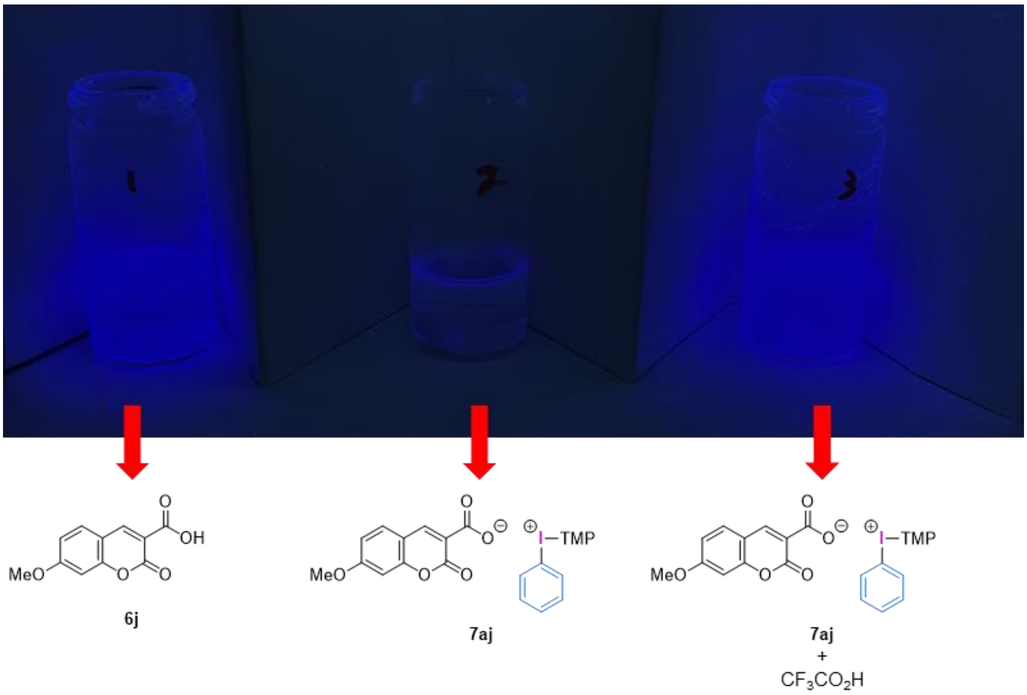

Figure S1. 365 nm UV light irradiation of 7-methoxycoumarin-3-carboxylic acid (**6j**, left), phenyl(2,4,6-trimethoxyphenyl)iodonium 7-methoxycoumarin-3-carboxylate (**7aj**, middle) and **7aj** + trifluoroacetic acid in MeOH (0.013 M) (right).

5. Reference

S1. Kikushima, K.; Miyamoto, N.; Watanabe, K.; Koseki, D.; Kita, Y.; Dohi, T. *Org. Lett.* **2022,** *24,* 1924–1928.

S2. Zhang, L.; Bao, W.; Liang, Y.; Pan, W.; Li, D.; Kong, L.; Wang, Z.-X.; Peng, B. *Angew. Chem. Int. Ed.* **2021,** *60,* 11414–11422.

S3. Dohi, T.; Koseki, D.; Sumida, K.; Okada, K.; Mizuno, S.; Kato, A.; Morimoto, K. Kita, Y. *Adv. Synth. Catal.* **2017,** *359,* 3503–3508.

S4. 18. Carreras, V.; Sandtorv, A. H.; Stuart, D. R. *J. Org. Chem.* **2017,** *82,* 1279–1284.

S5. Petersen, T. B.; Khan, R.; Olofsson, B. *Org. Lett.* **2011,** *13,* 3462–3465.

S6. Zhang, J.; Zhang, Z.; Wang, Y.; Zheng, X.; Wang, Z. *Eur. J. Org. Chem.* **2008,** 5112–5116.

S7. Lucchetti, N.; Scalone, M.; Fantasia, S.; Muñiz, K. *Adv. Synth. Catal.* **2016,** *358,* 2093–2099.

6. NMR Charts

Phenyl(2,4-dimethoxyphenyl)iodonium acetate (3ad)

^{1}H NMR (400 MHz, CDCl$_3$)

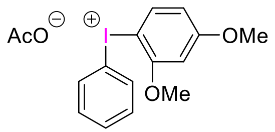

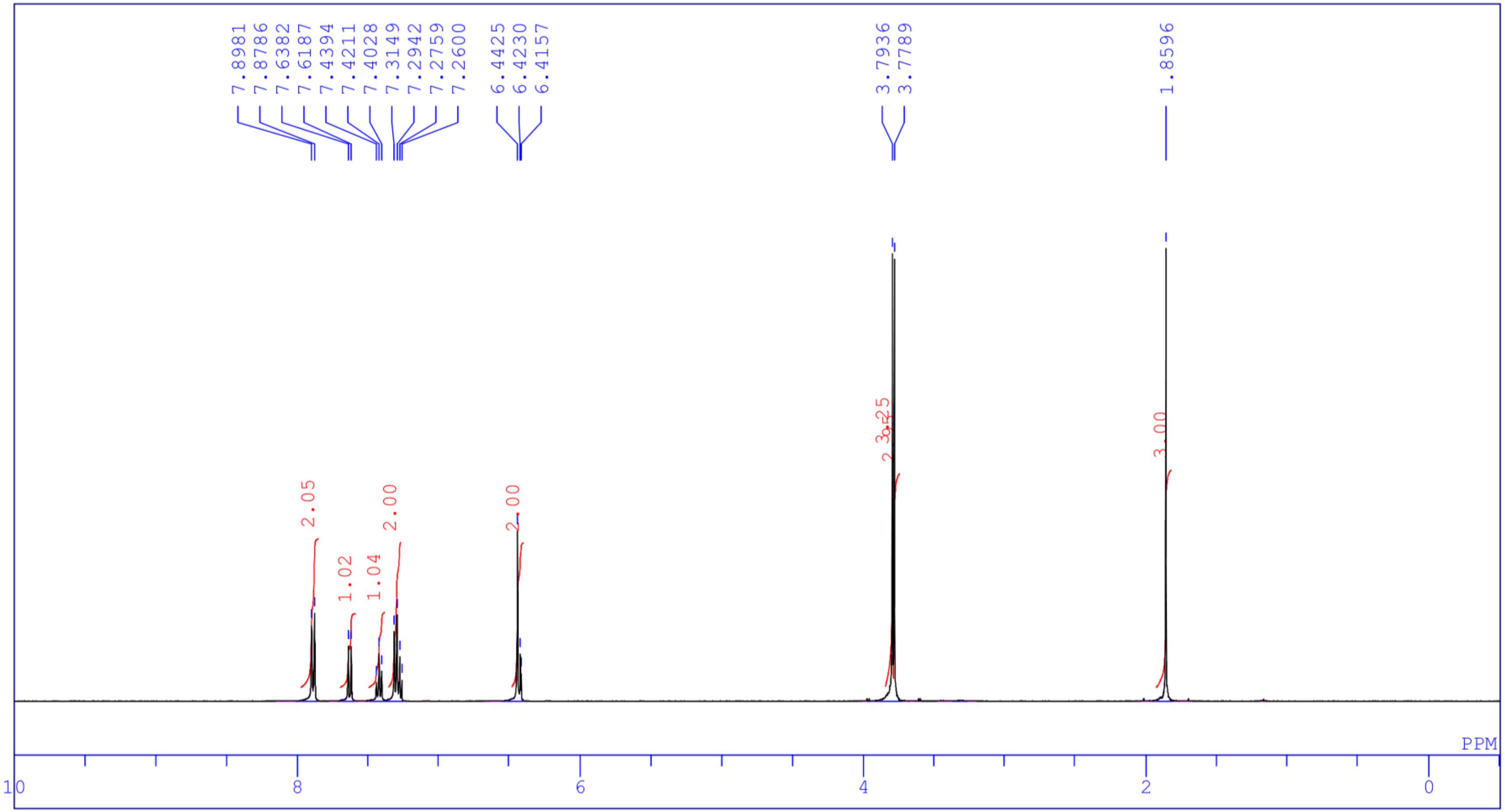

Phenyl(2,4-dimethoxyphenyl)iodonium acetate (3ad)

^{13}C NMR (100 MHz, $CDCl_3$)

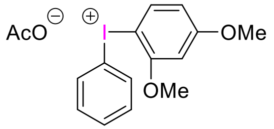

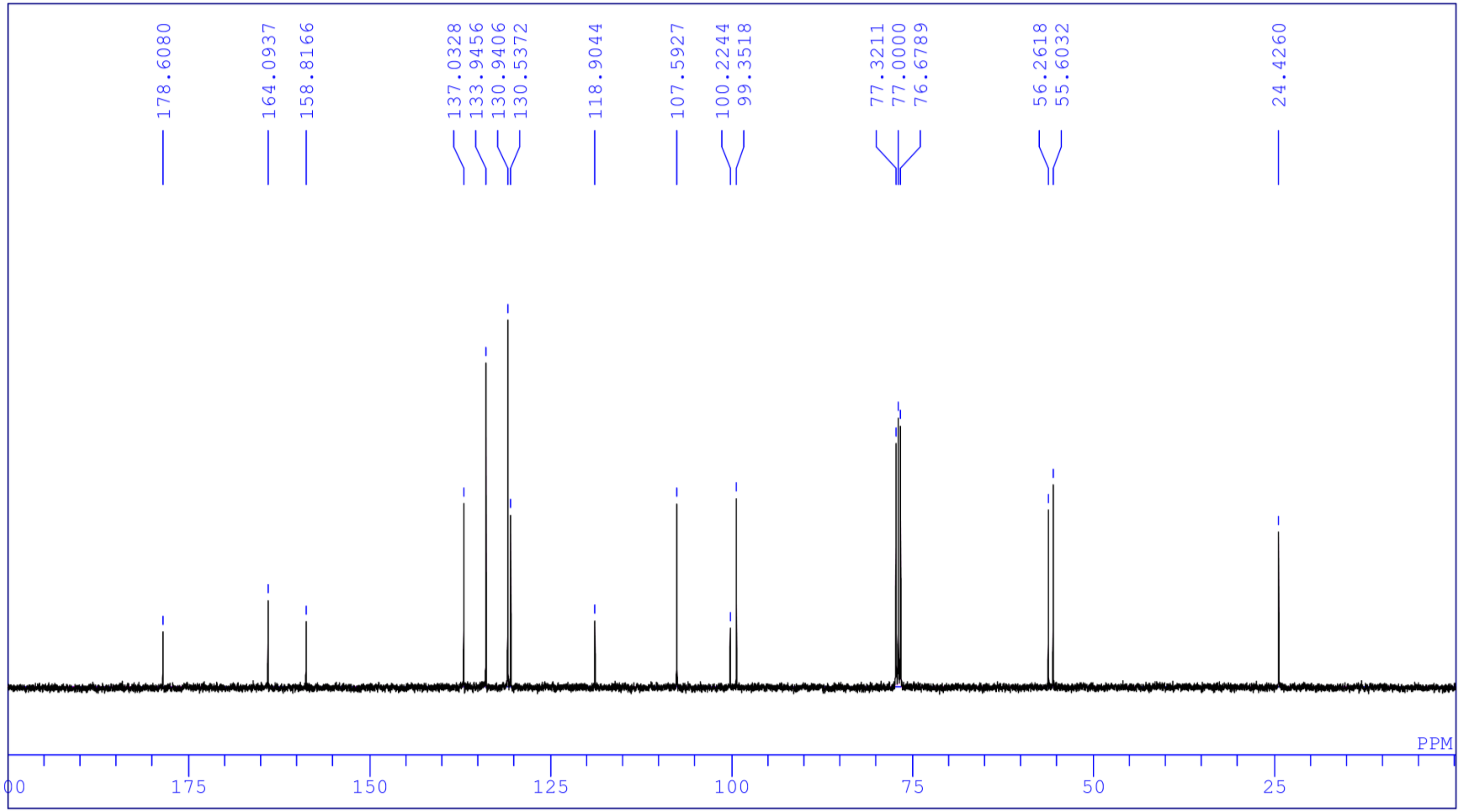

Phenyl(2,4,6-trimethoxyphenyl)iodonium acetate (3ae)

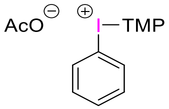

^{1}H NMR (400 MHz, $CDCl_3$)

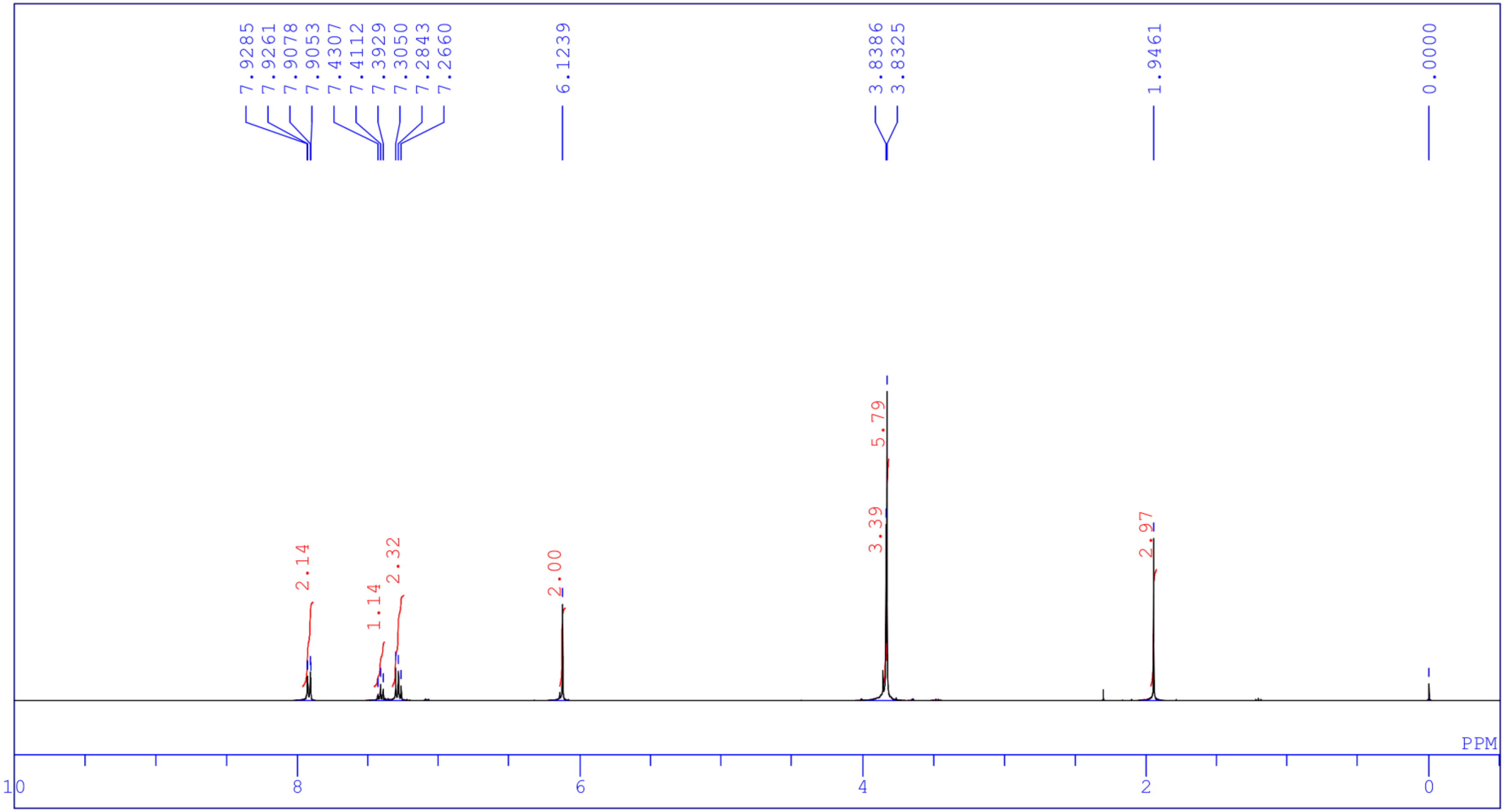

Phenyl(2,4,6-trimethoxyphenyl)iodonium acetate (3ae)

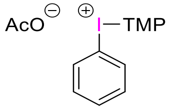

^{13}C NMR (100 MHz, CDCl3)

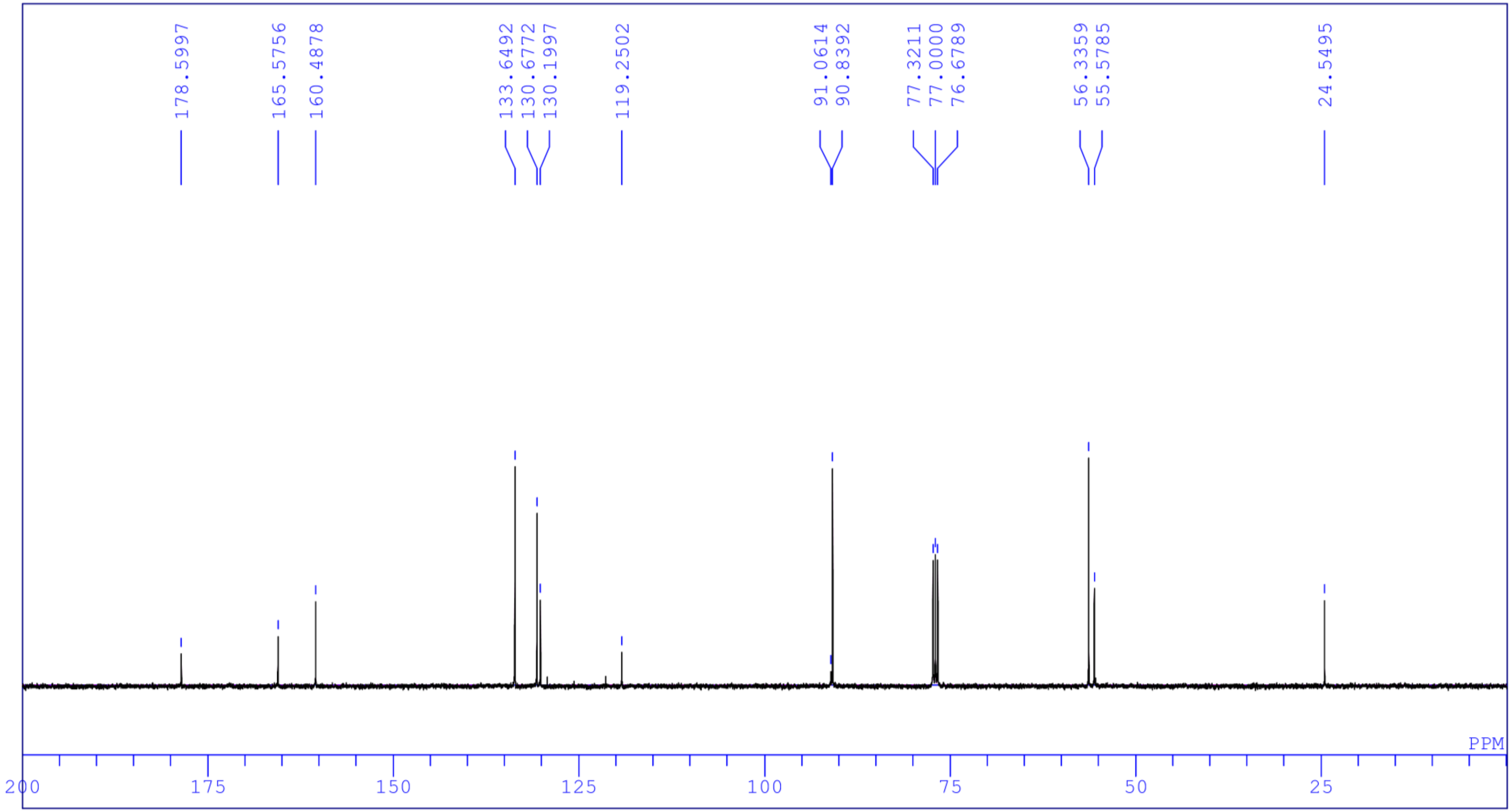

4-Methylphenyl(2,4,6-trimethoxyphenyl)iodonium acetate (4b)

^{1}H NMR (400 MHz, CDCl$_3$)

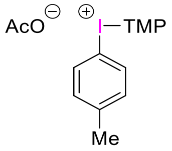

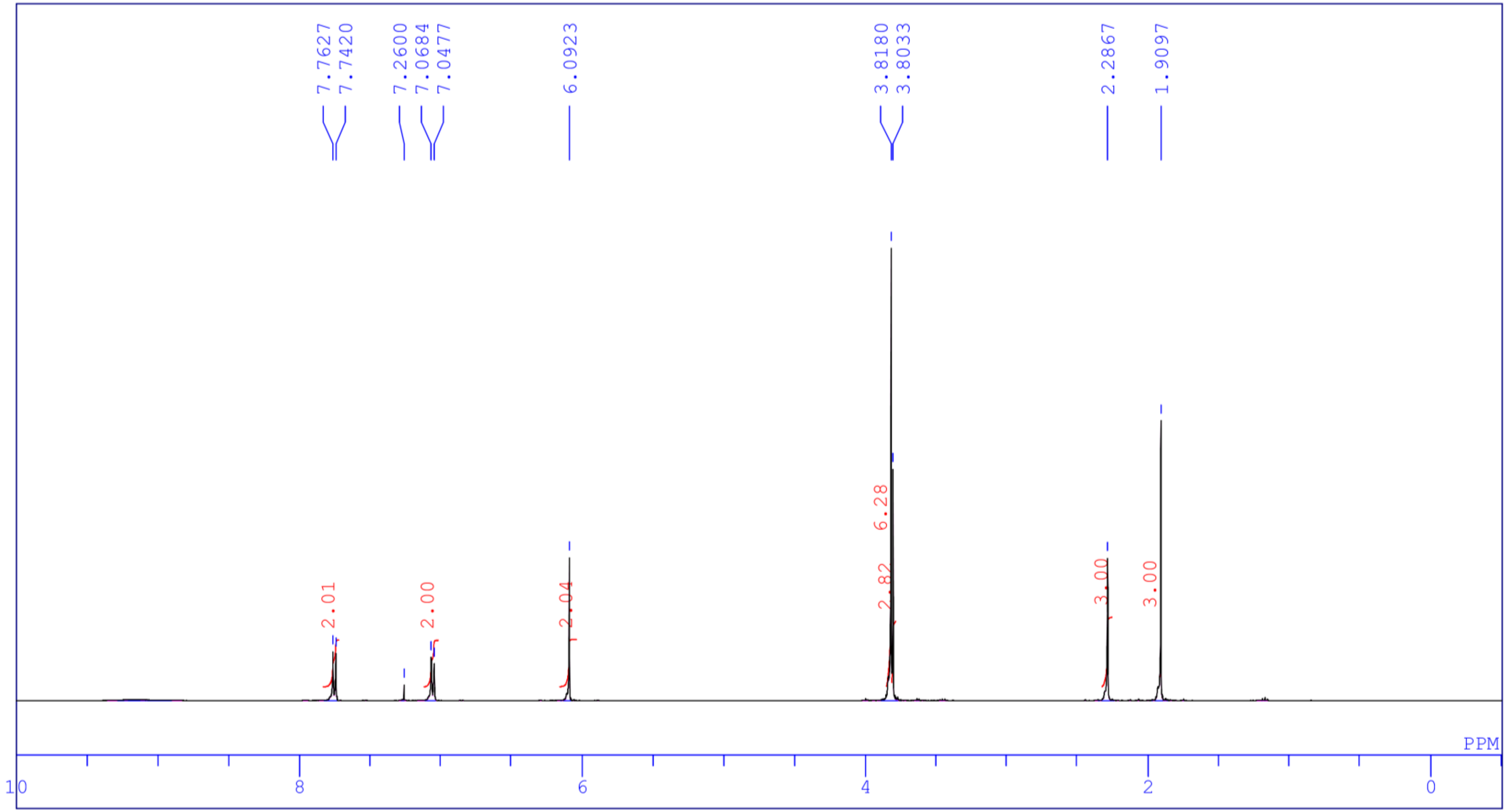

4-Methylphenyl(2,4,6-trimethoxyphenyl)iodonium acetate (4b)

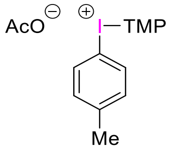

^{13}C NMR (100 MHz, CDCl3)

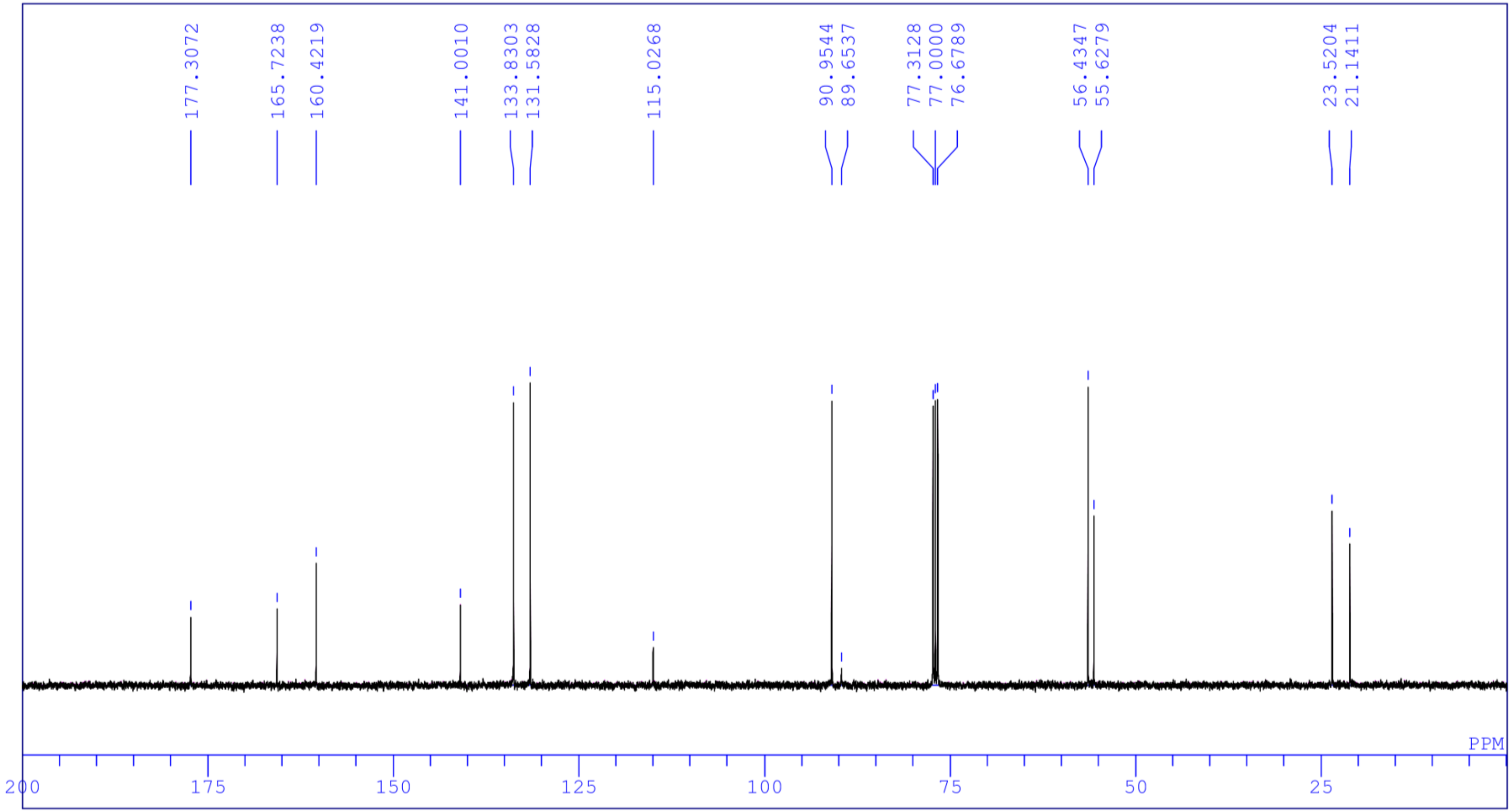

4-Methoxyphenyl(2,4,6-trimethoxyphenyl)iodonium acetate (4c)

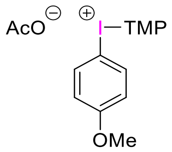

^{1}H NMR (400 MHz, CDCl3)

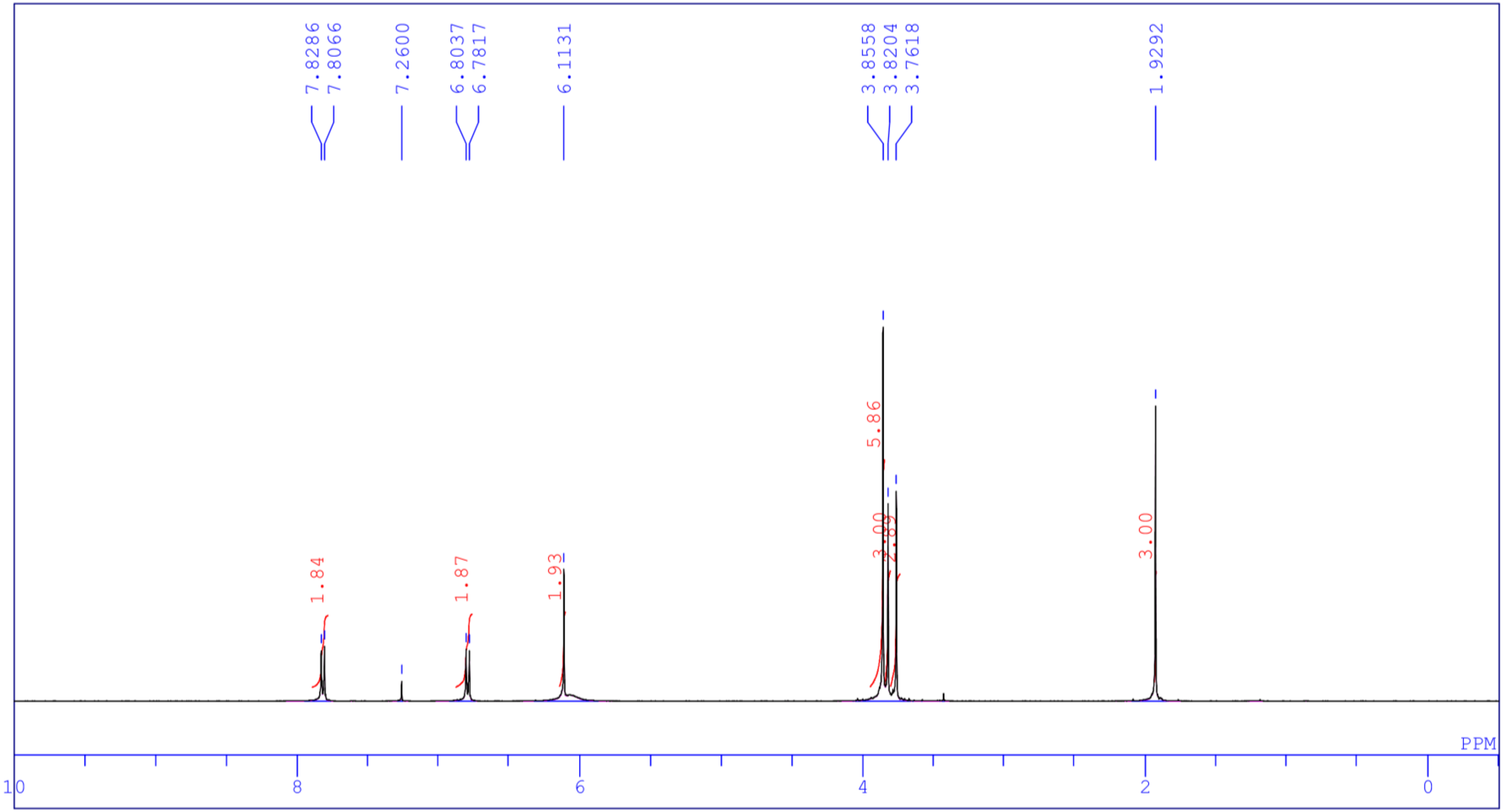

4-Methoxyphenyl(2,4,6-trimethoxyphenyl)iodonium acetate (4c)

^{13}C NMR (100 MHz, $CDCl_3$)

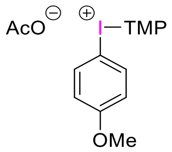

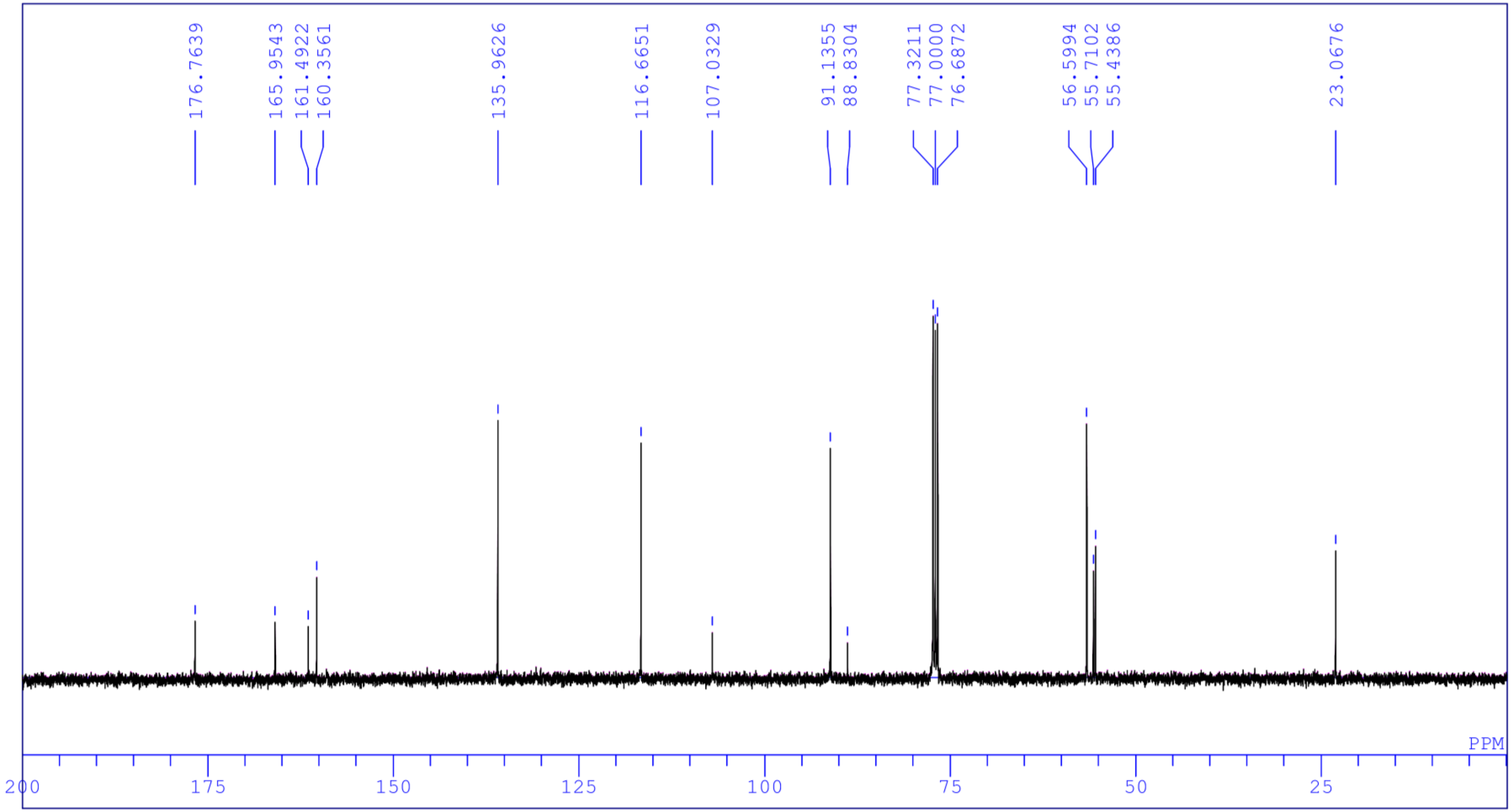

4,4'-Biphenyl(2,4,6-trimethoxyphenyl)iodonium acetate (4d)

^{1}H NMR (400 MHz, CDCl3)

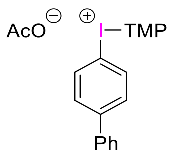

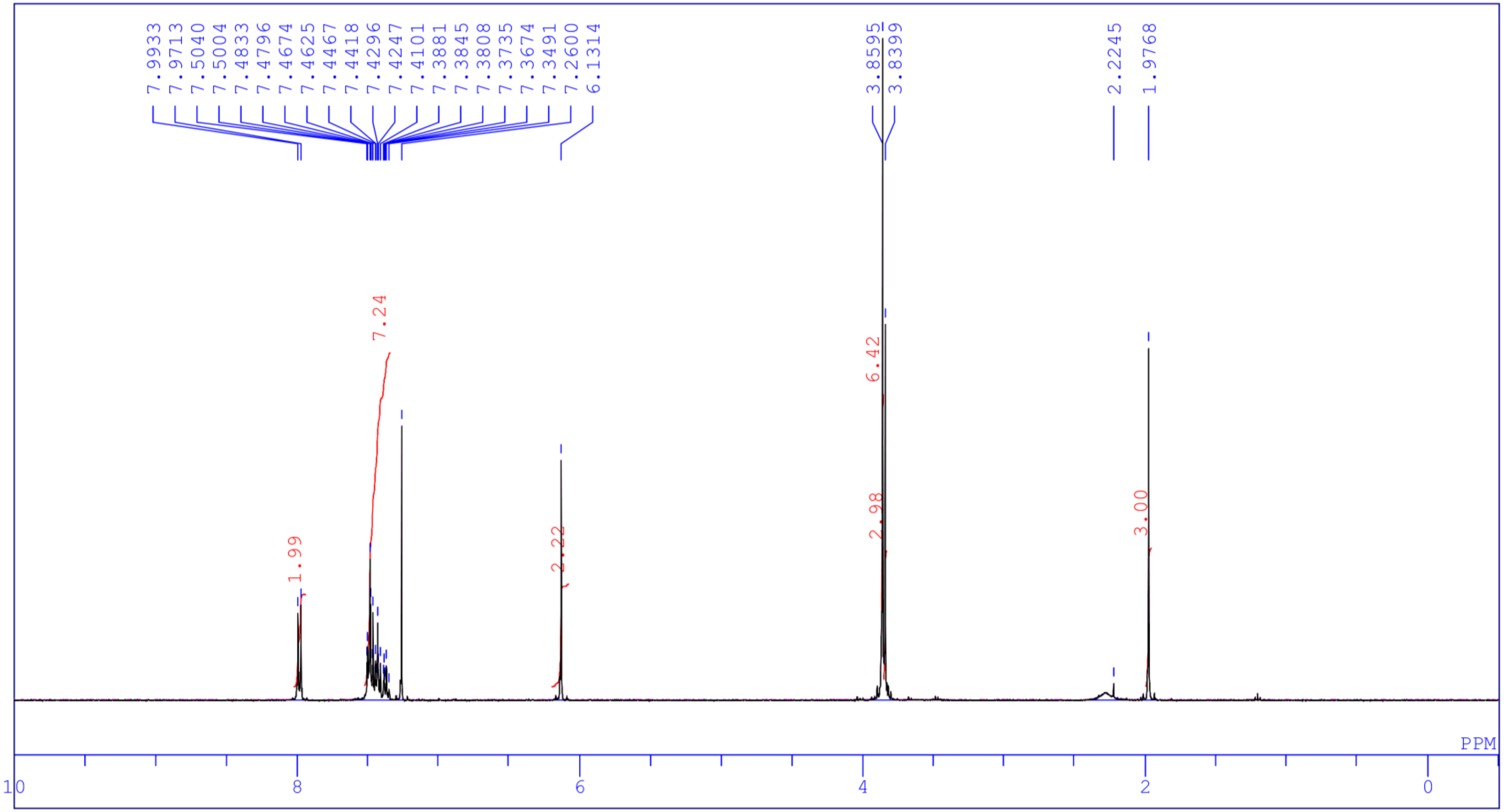

4,4'-Biphenyl(2,4,6-trimethoxyphenyl)iodonium acetate (4d)

^{13}C NMR (100 MHz, CDCl$_3$)

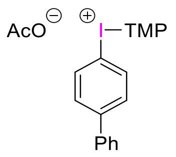

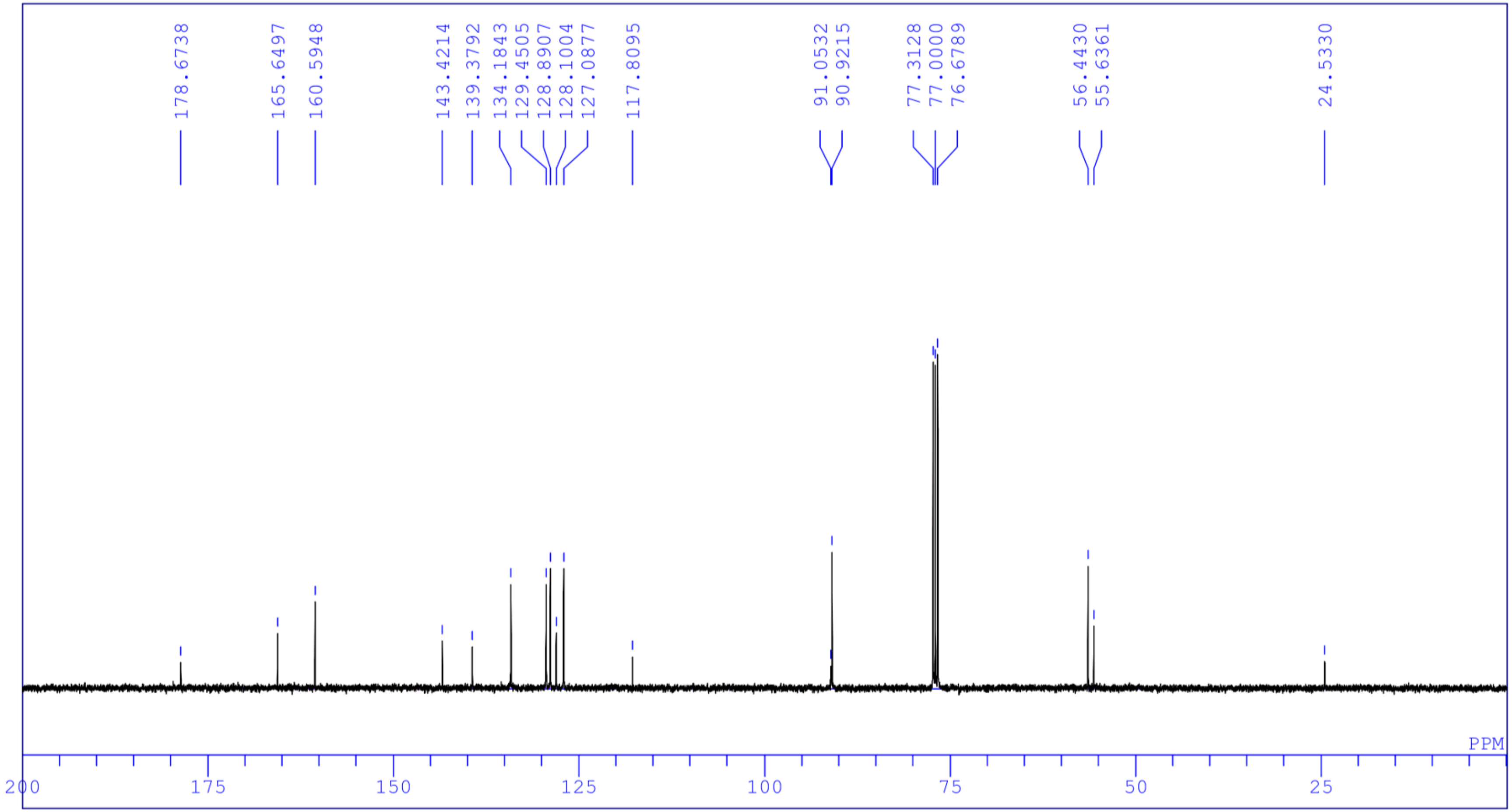

4-Methoxycarbonylphenyl(2,4,6-trimethoxyphenyl)iodonium acetate (4e)

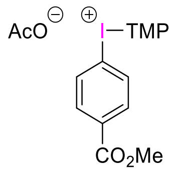

^{1}H NMR (400 MHz, $CDCl_3$)

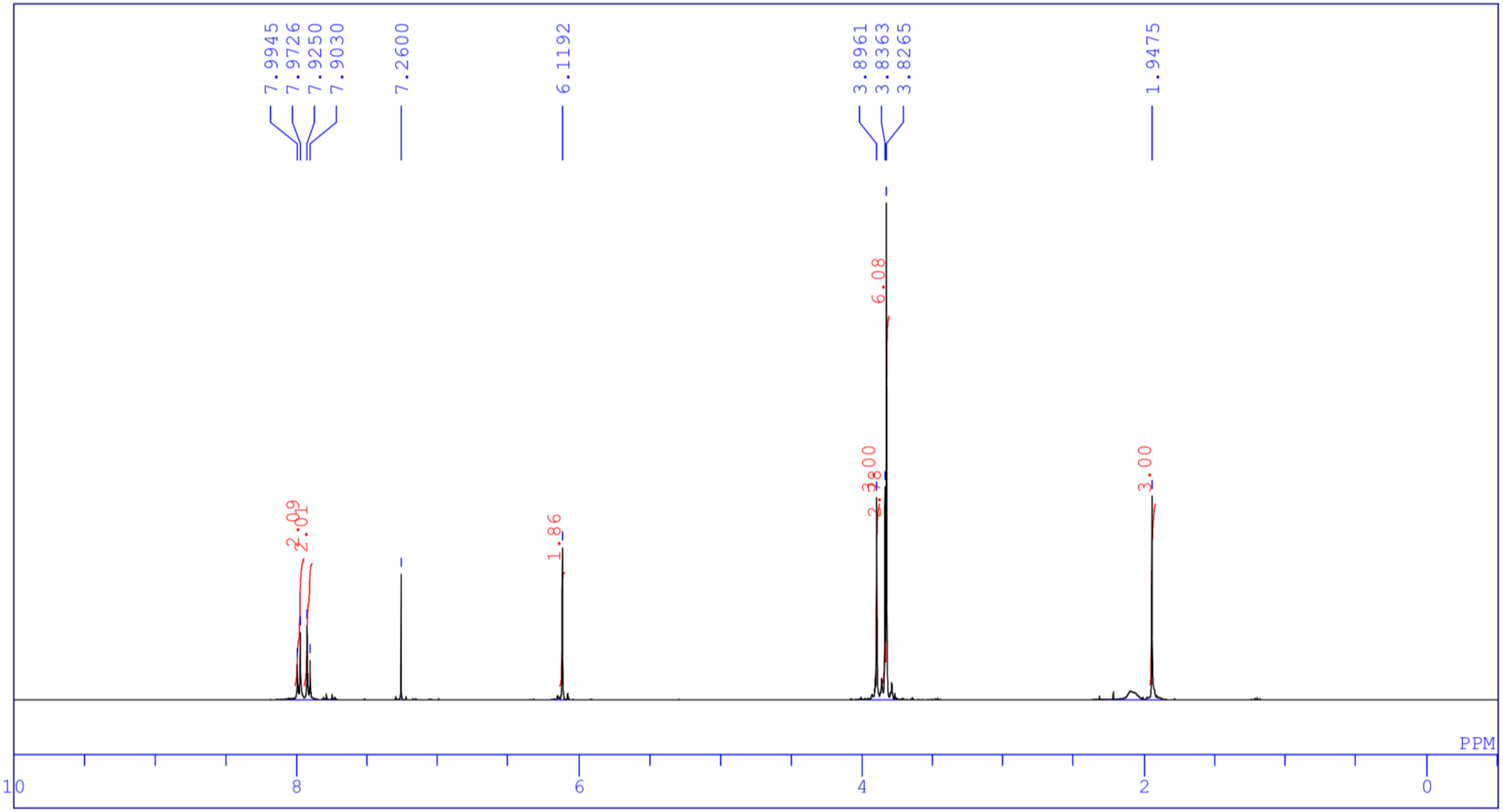

4-Methoxycarbonylphenyl(2,4,6-trimethoxyphenyl)iodonium acetate (4e)

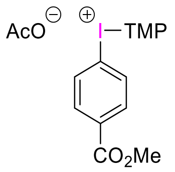

^{13}C NMR (100 MHz, $CDCl_3$)

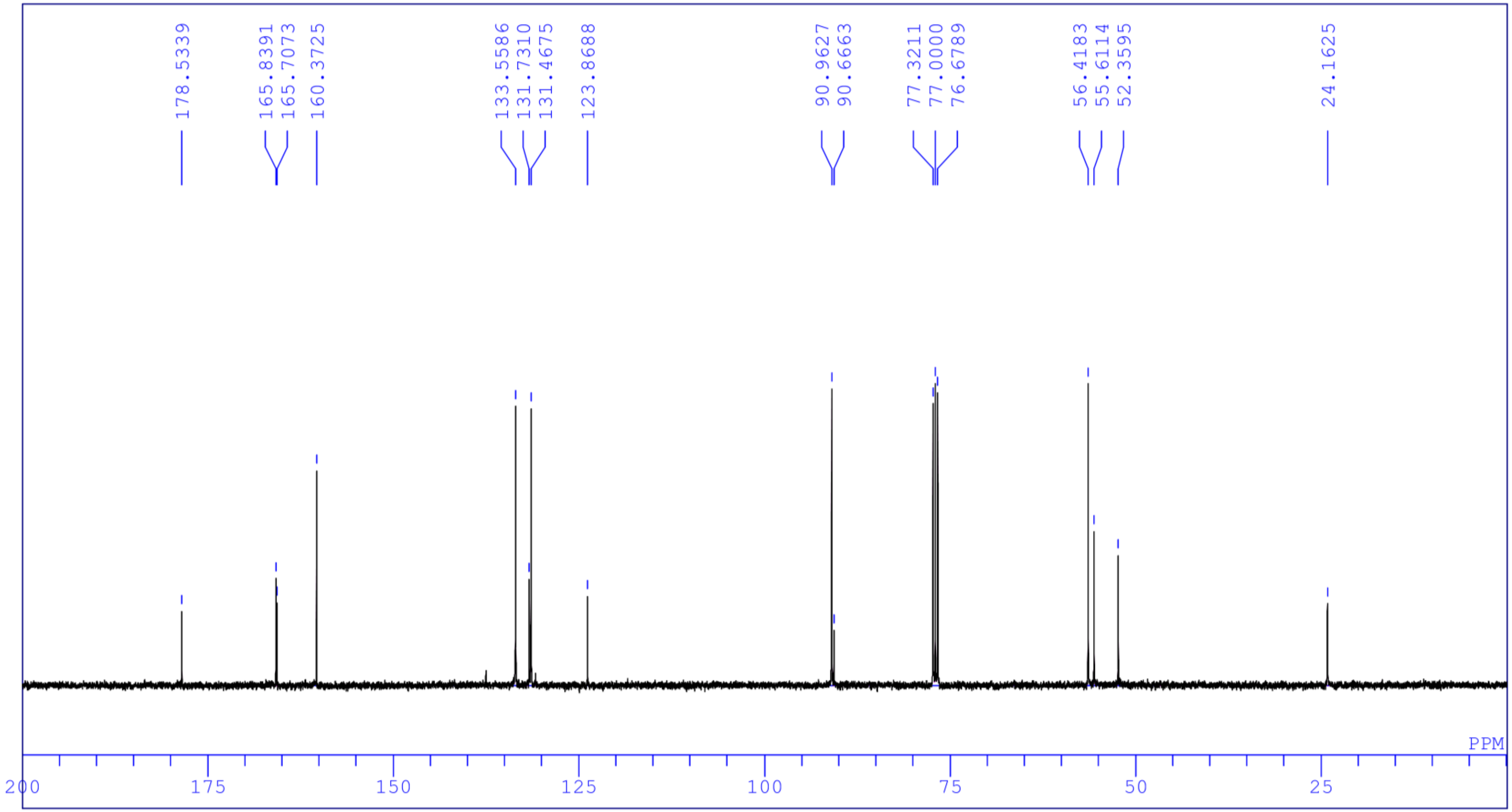

4-Nitorophenyl(2,4,6-trimethoxyphenyl)iodonium acetate (4f)

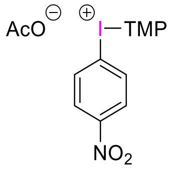

^{1}H NMR (400 MHz, $CDCl_3$)

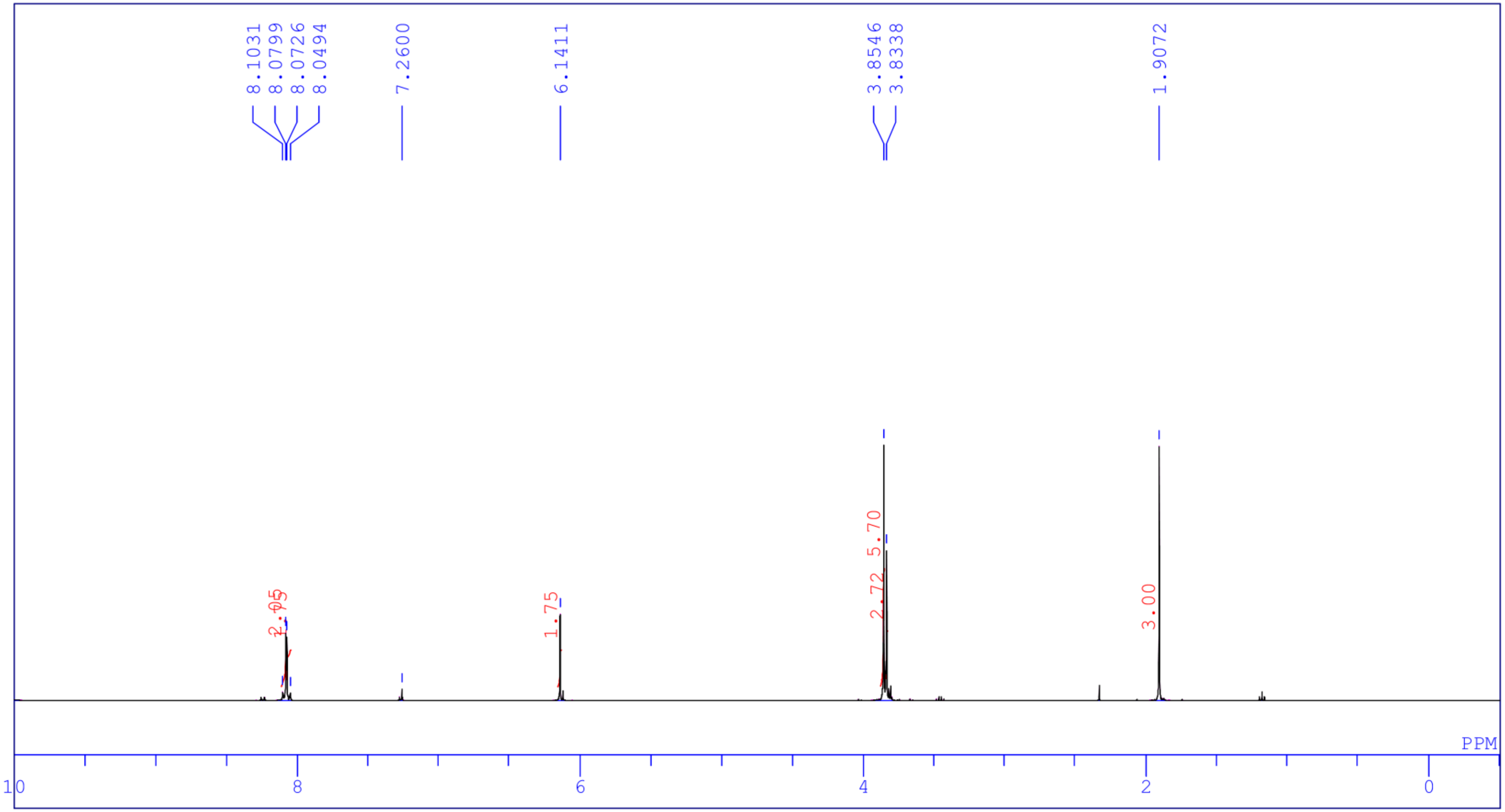

4-Nitorophenyl(2,4,6-trimethoxyphenyl)iodonium acetate (4f)

^{13}C NMR (100 MHz, $CDCl_3$)

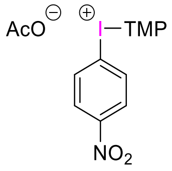

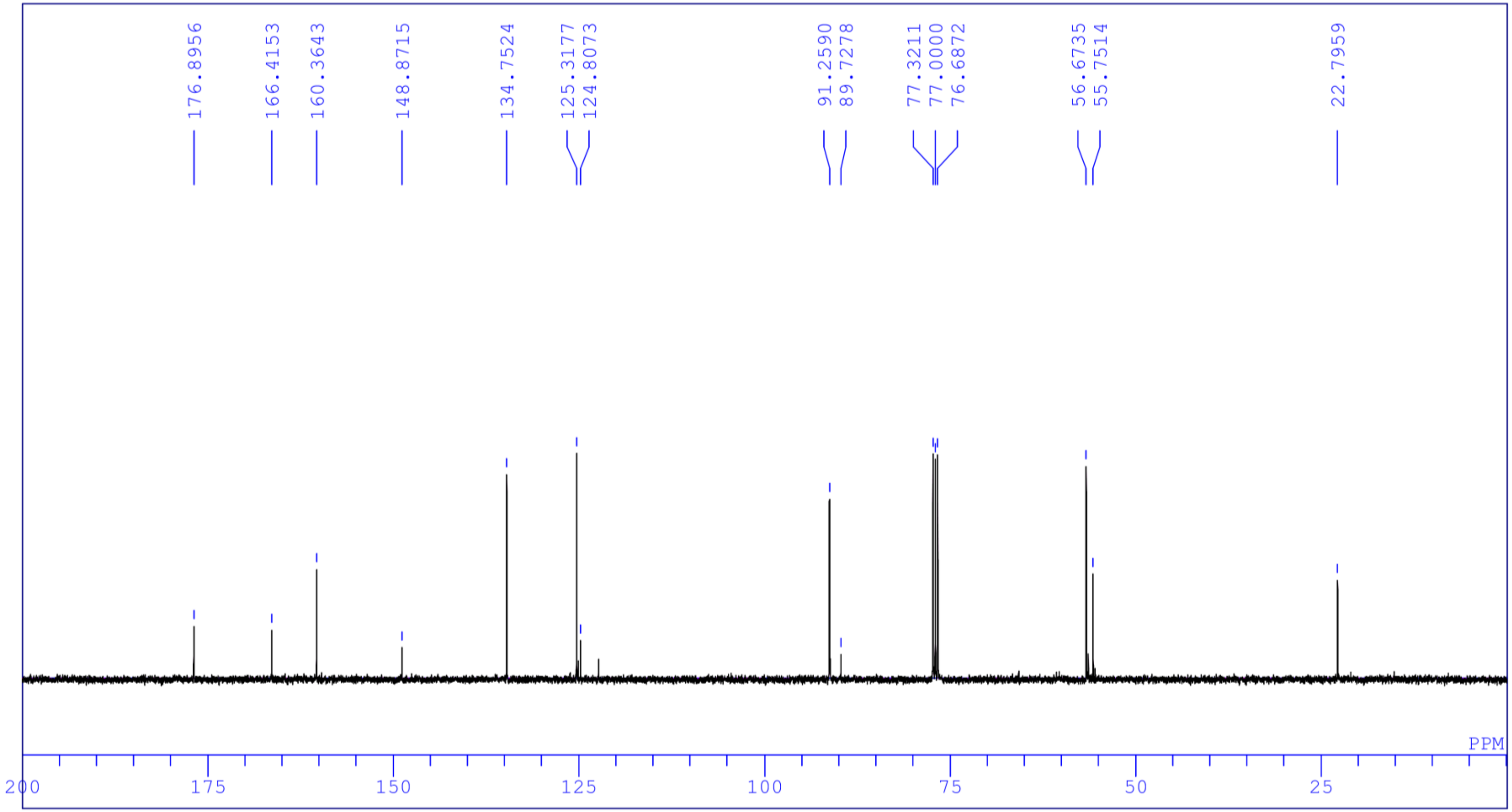

2-Fluorophenyl(2,4,6-trimethoxyphenyl)iodonium acetate (4g)

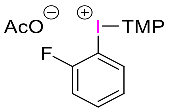

^{1}H NMR (400 MHz, $CDCl_3$)

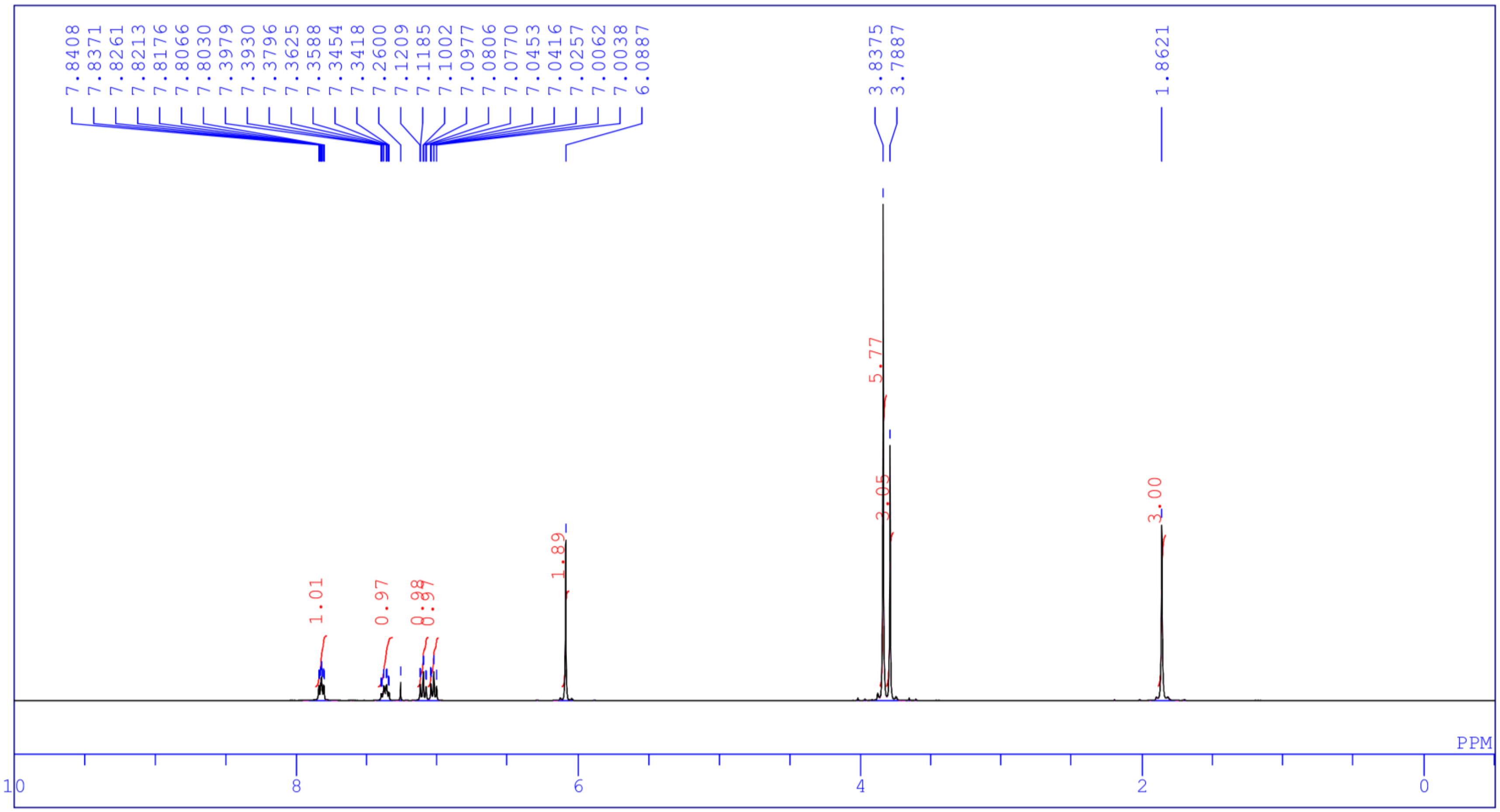

2-Fluorophenyl(2,4,6-trimethoxyphenyl)iodonium acetate (4g)

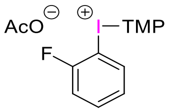

^{13}C NMR (100 MHz, $CDCl_3$)

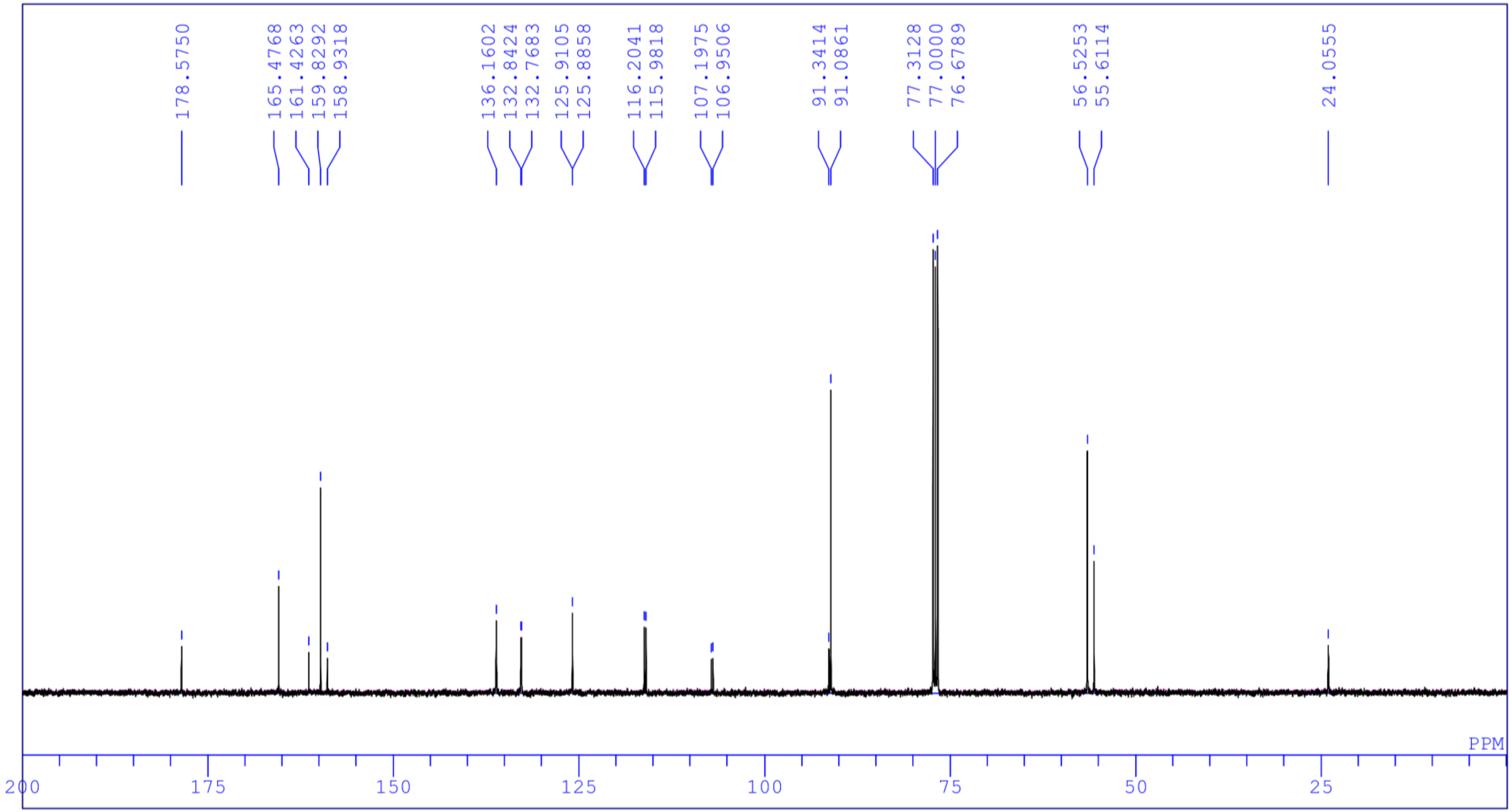

2-Fluorophenyl(2,4,6-trimethoxyphenyl)iodonium acetate (4g)

^{19}F NMR (376 MHz, $CDCl_3$)

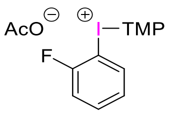

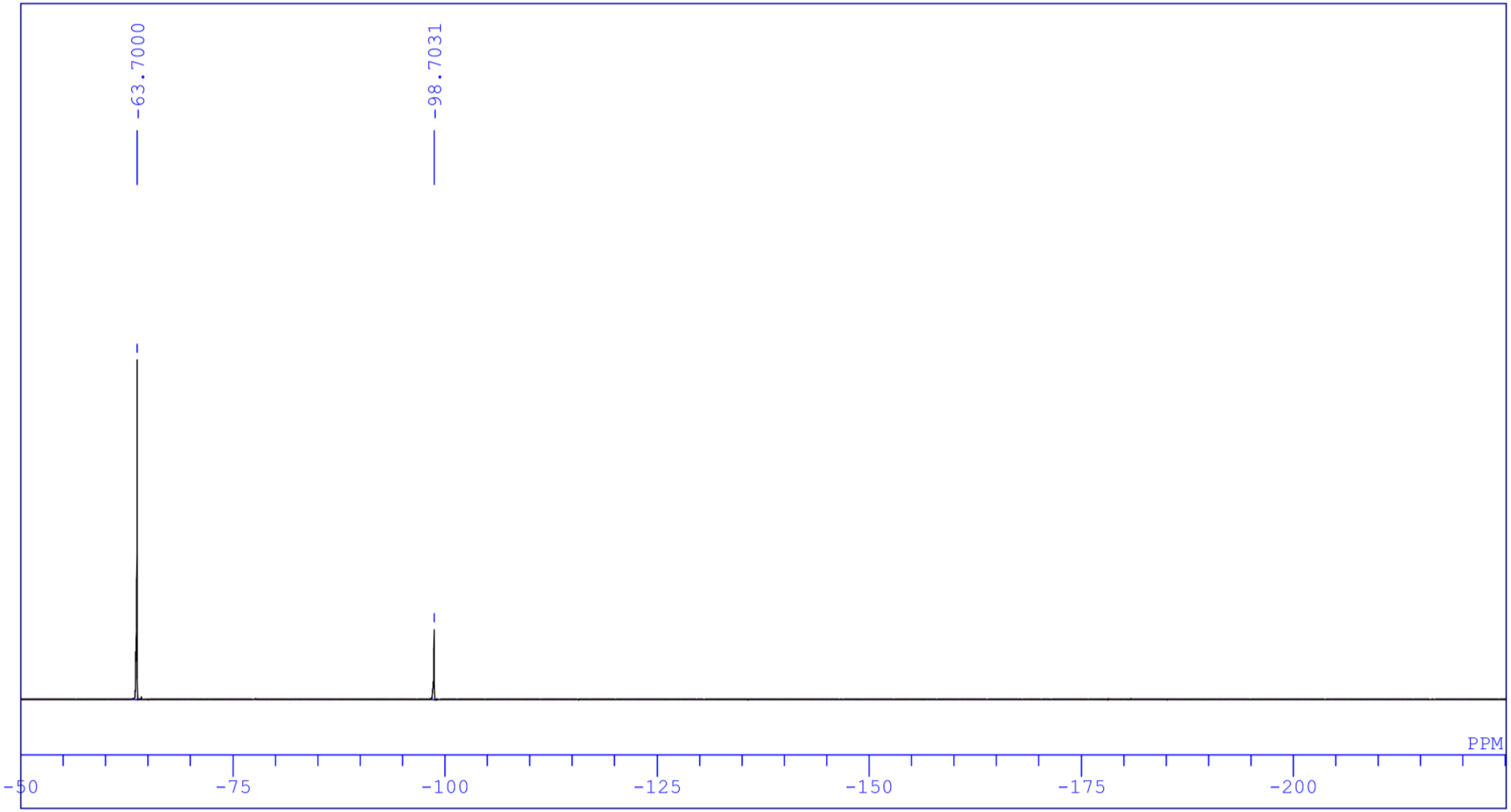

2,6-Dimethylphenyl(2,4,6-trimethoxyphenyl)iodonium acetate (4h)

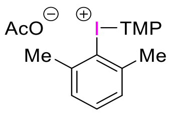

^{1}H NMR (400 MHz, $CDCl_3$)

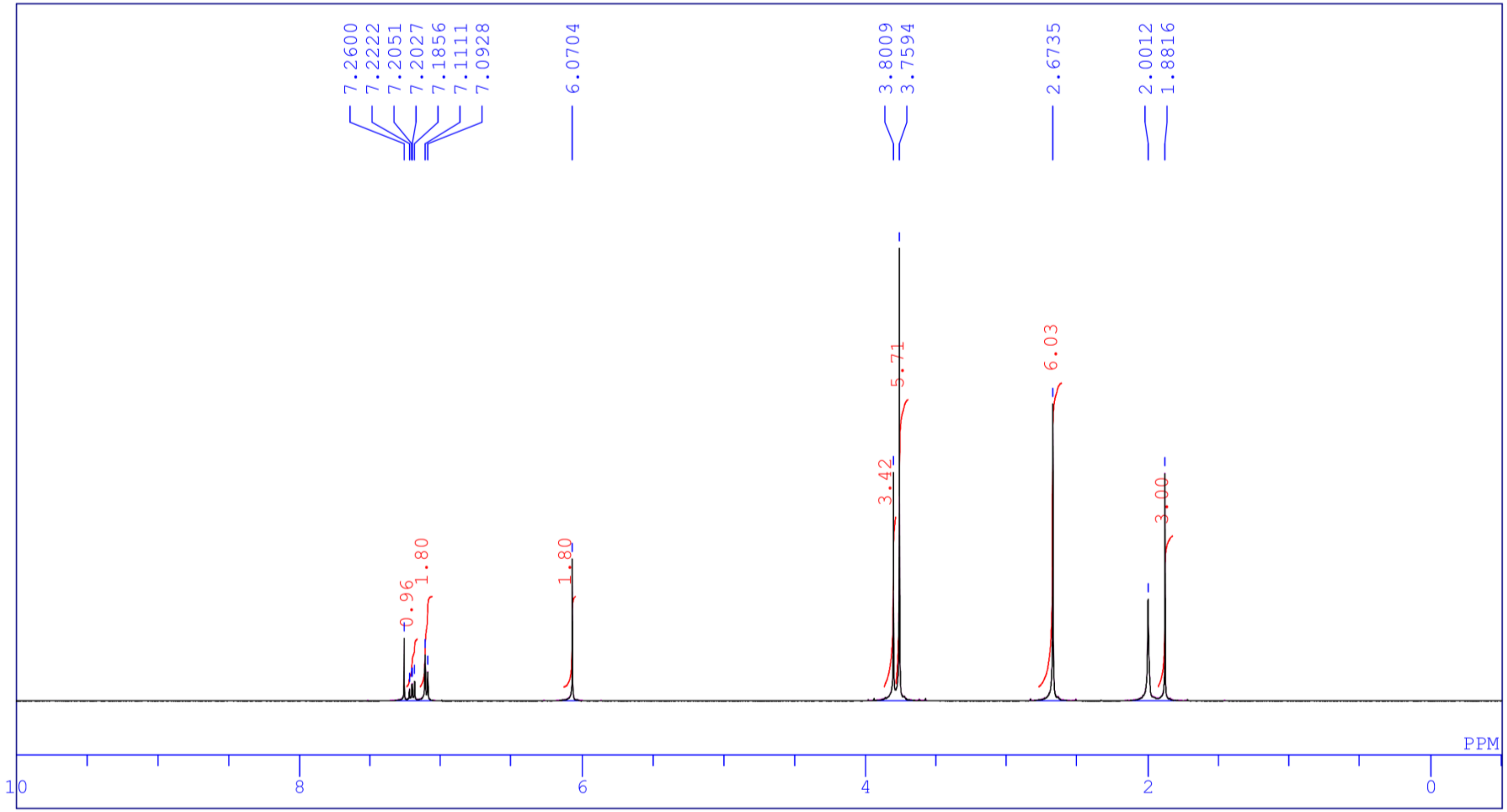

2,6-Dimethylphenyl(2,4,6-trimethoxyphenyl)iodonium acetate (4h)

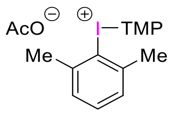

^{13}C NMR (100 MHz, CDCl$_3$)

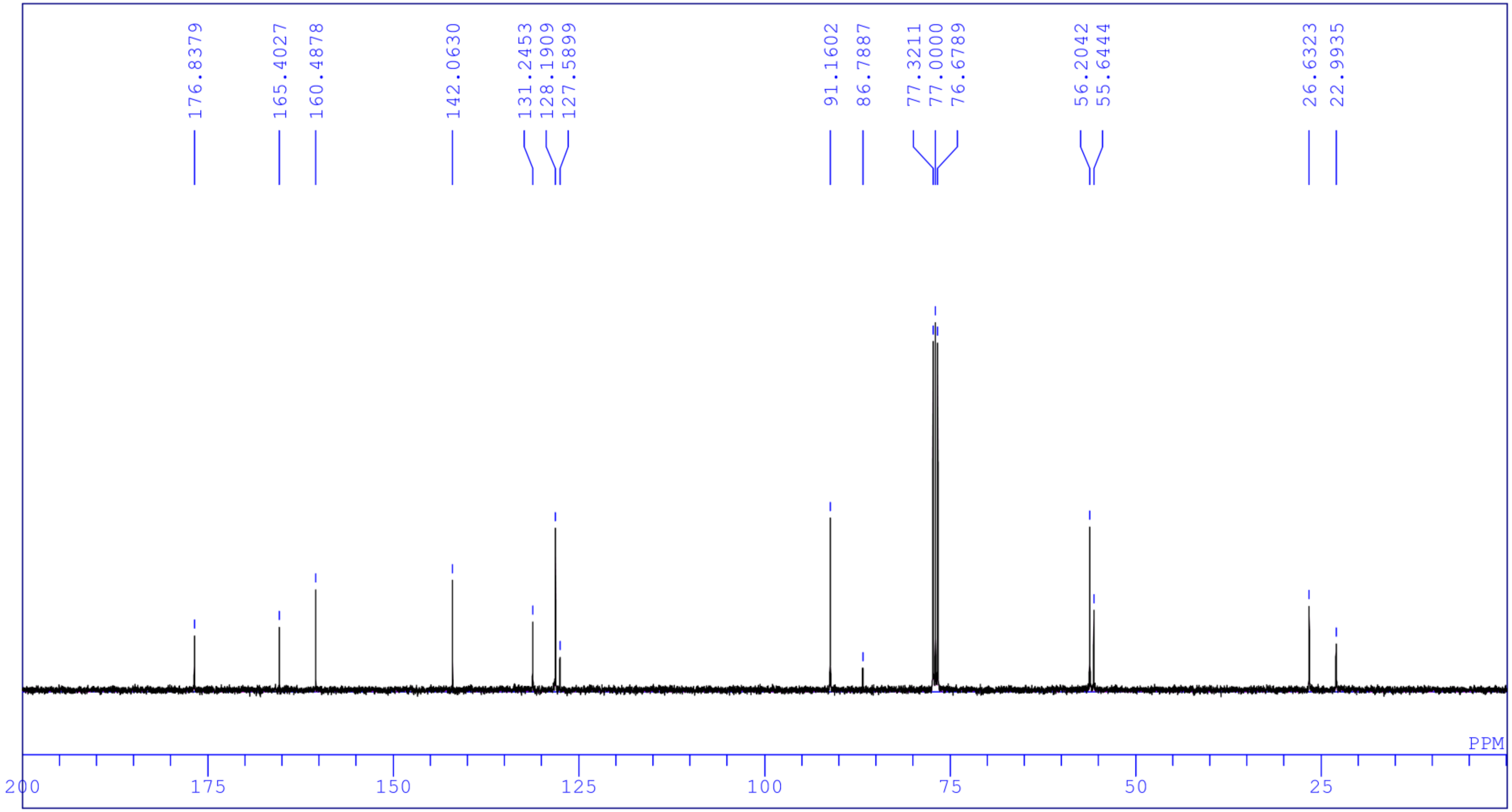

3-[[*N*-(1,1-dimethylethoxy)carbonyl]amino]phenyl(2,4,6-trimethoxyphenyl)iodonium acetate (4i)

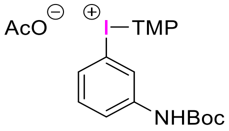

^{1}H NMR (400 MHz, $CDCl_3$)

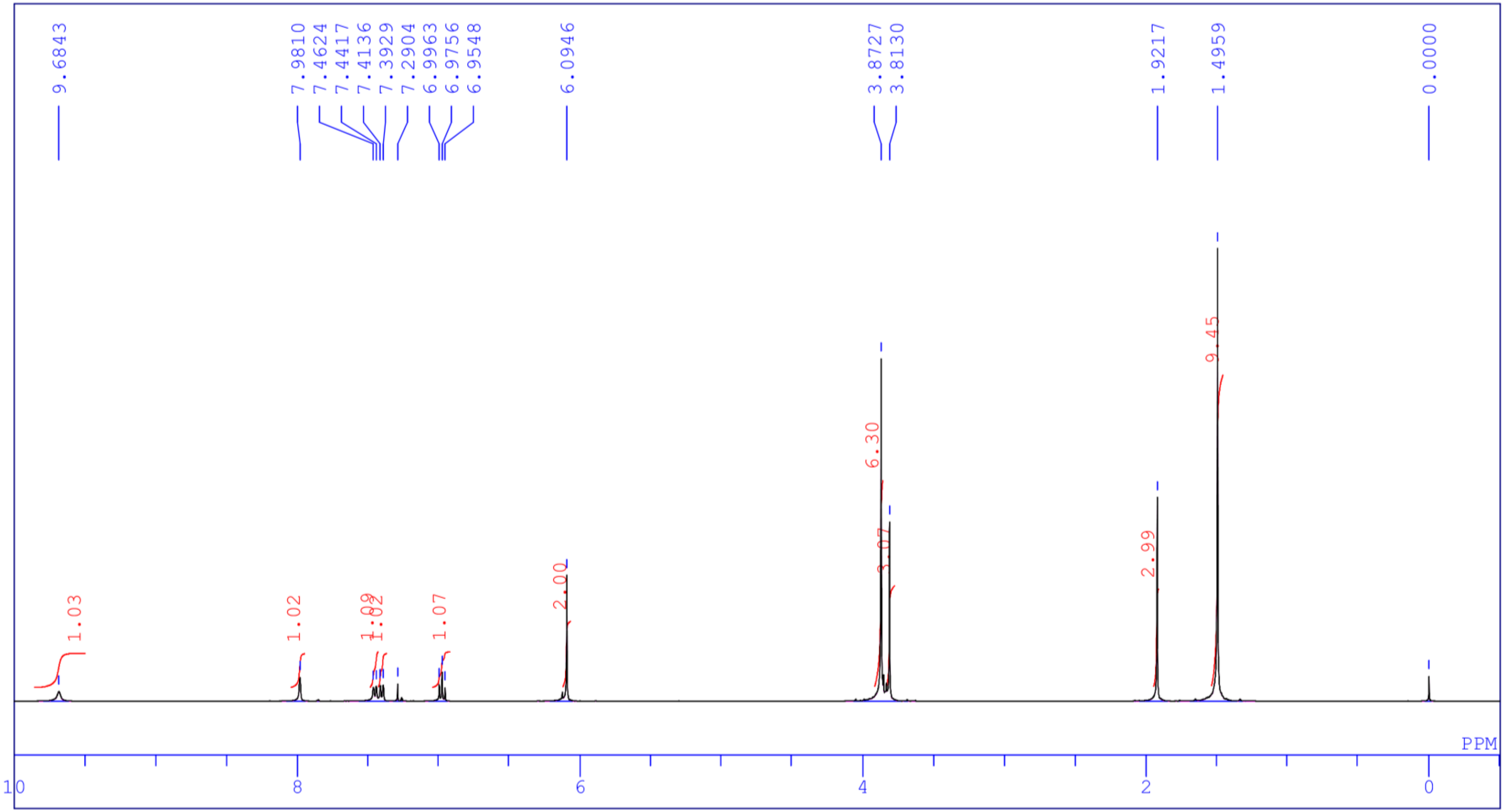

3-[[*N*-(1,1-dimethylethoxy)carbonyl]amino]phenyl(2,4,6-trimethoxyphenyl)iodonium acetate (4i)

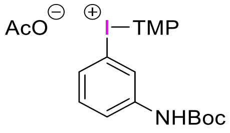

^{13}C NMR (100 MHz, $CDCl_3$)

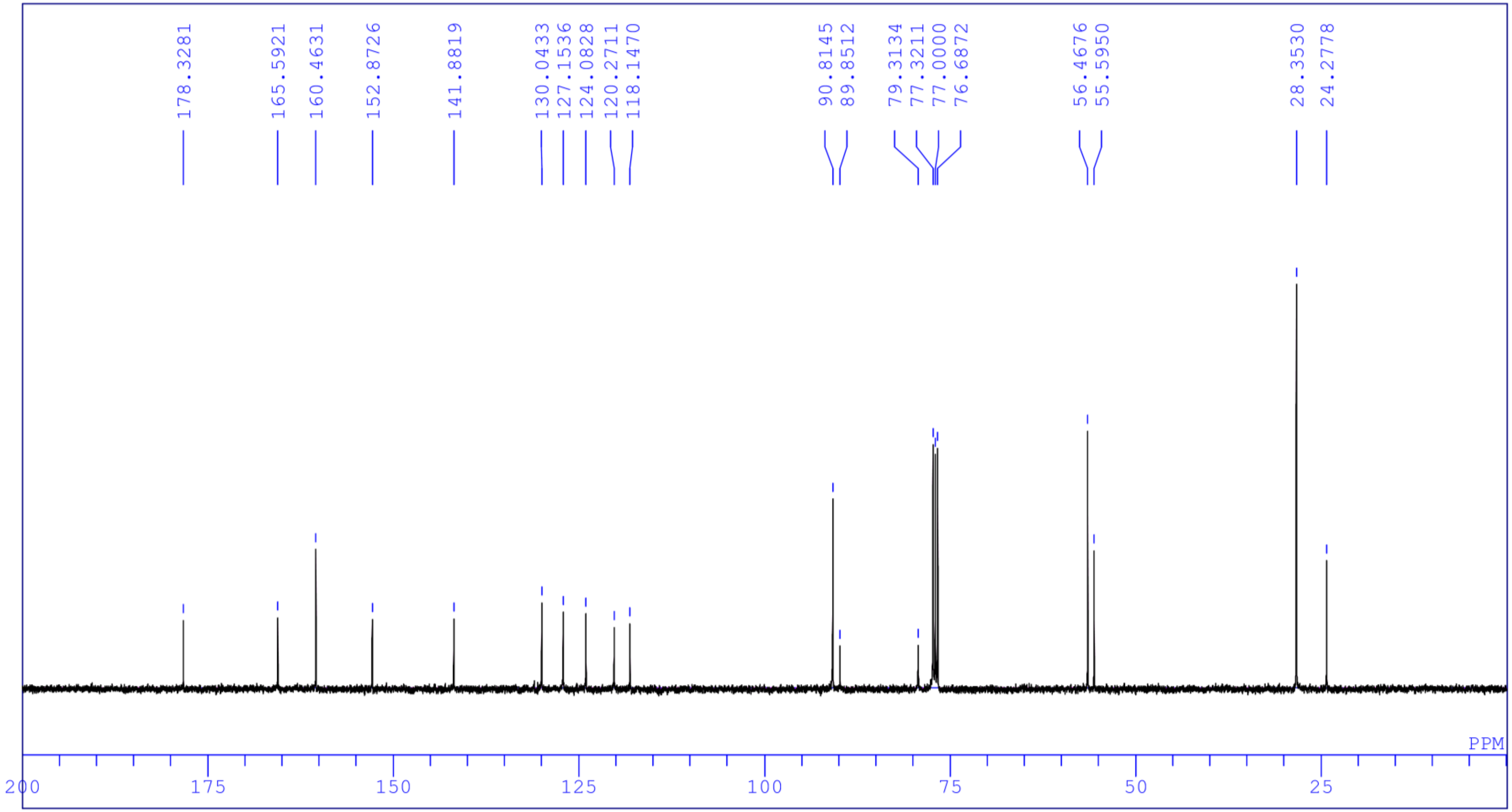

2-Chloropyridine-5-yl(2,4,6-trimethoxyphenyl)iodonium acetate (4j)

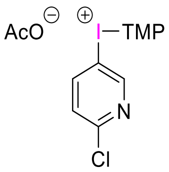

^{1}H NMR (400 MHz, $CDCl_3$)

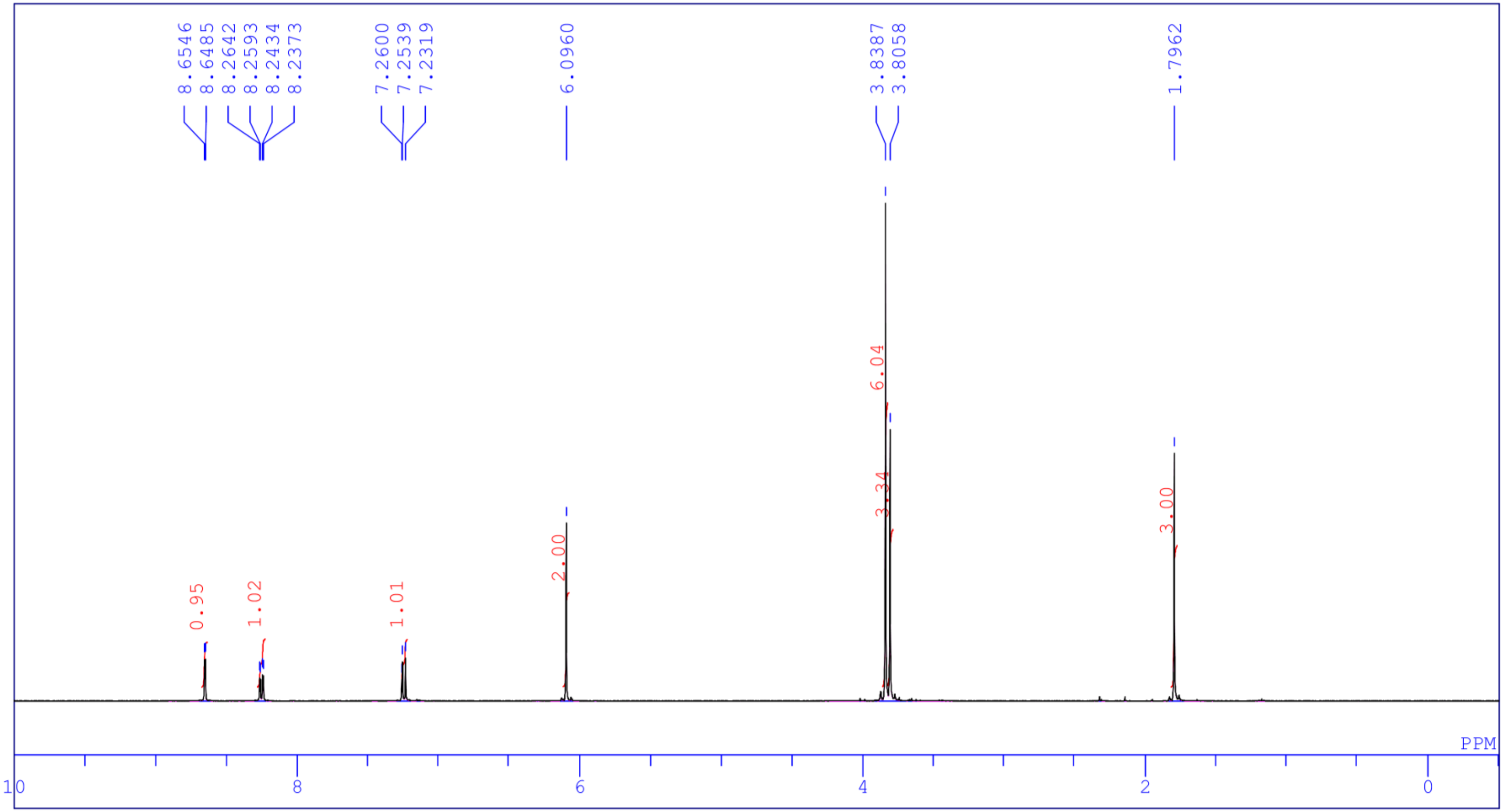

2-Chloropyridine-5-yl(2,4,6-trimethoxyphenyl)iodonium acetate (4j)

^{13}C NMR (100 MHz, CDCl$_3$)

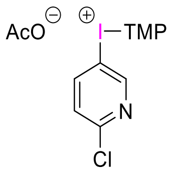

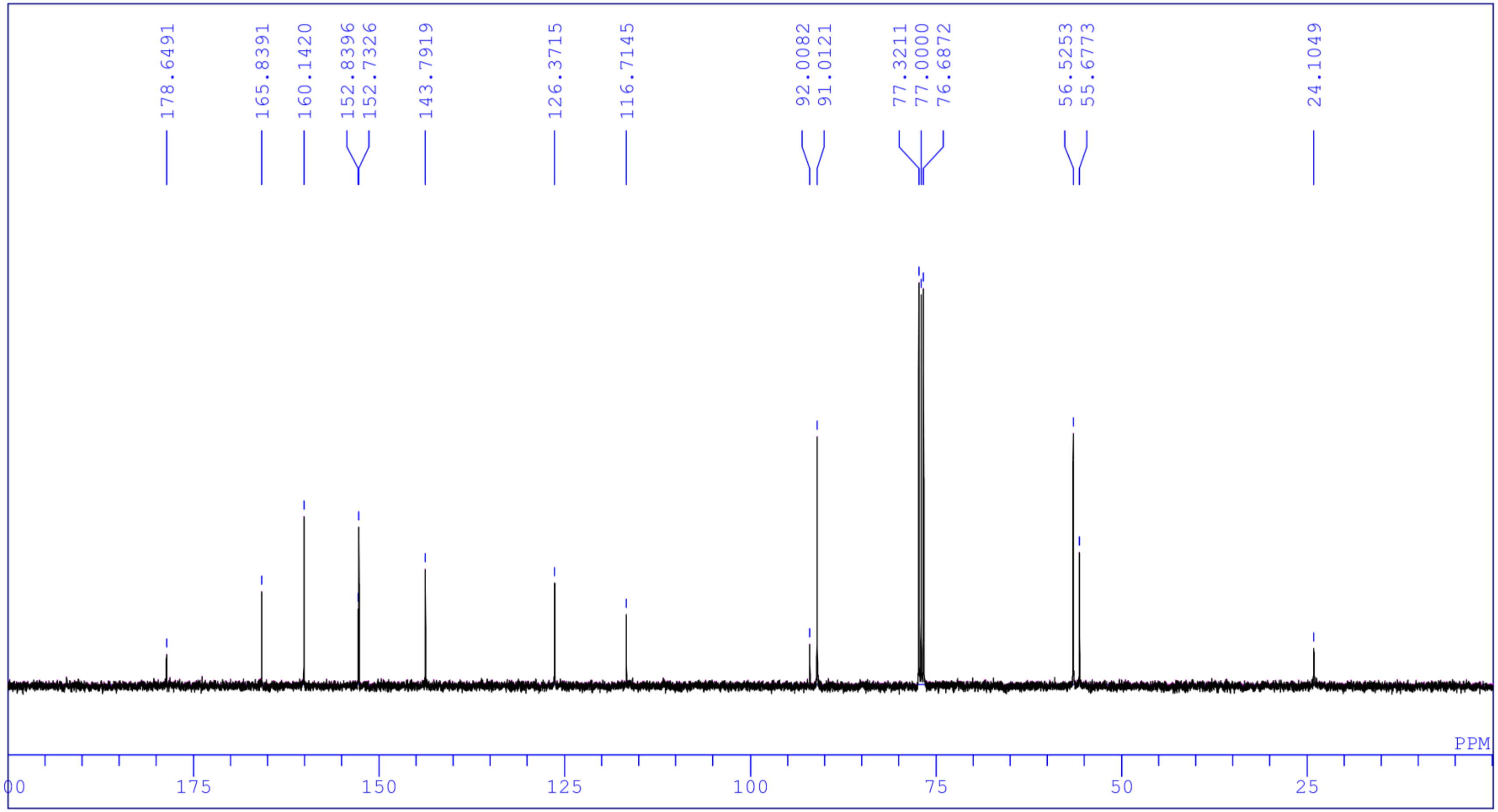

3-Thienyl(2,4,6-trimethoxyphenyl)iodonium acetate (4k)

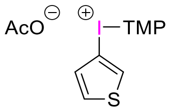

^{1}H NMR (400 MHz, $CDCl_3$)

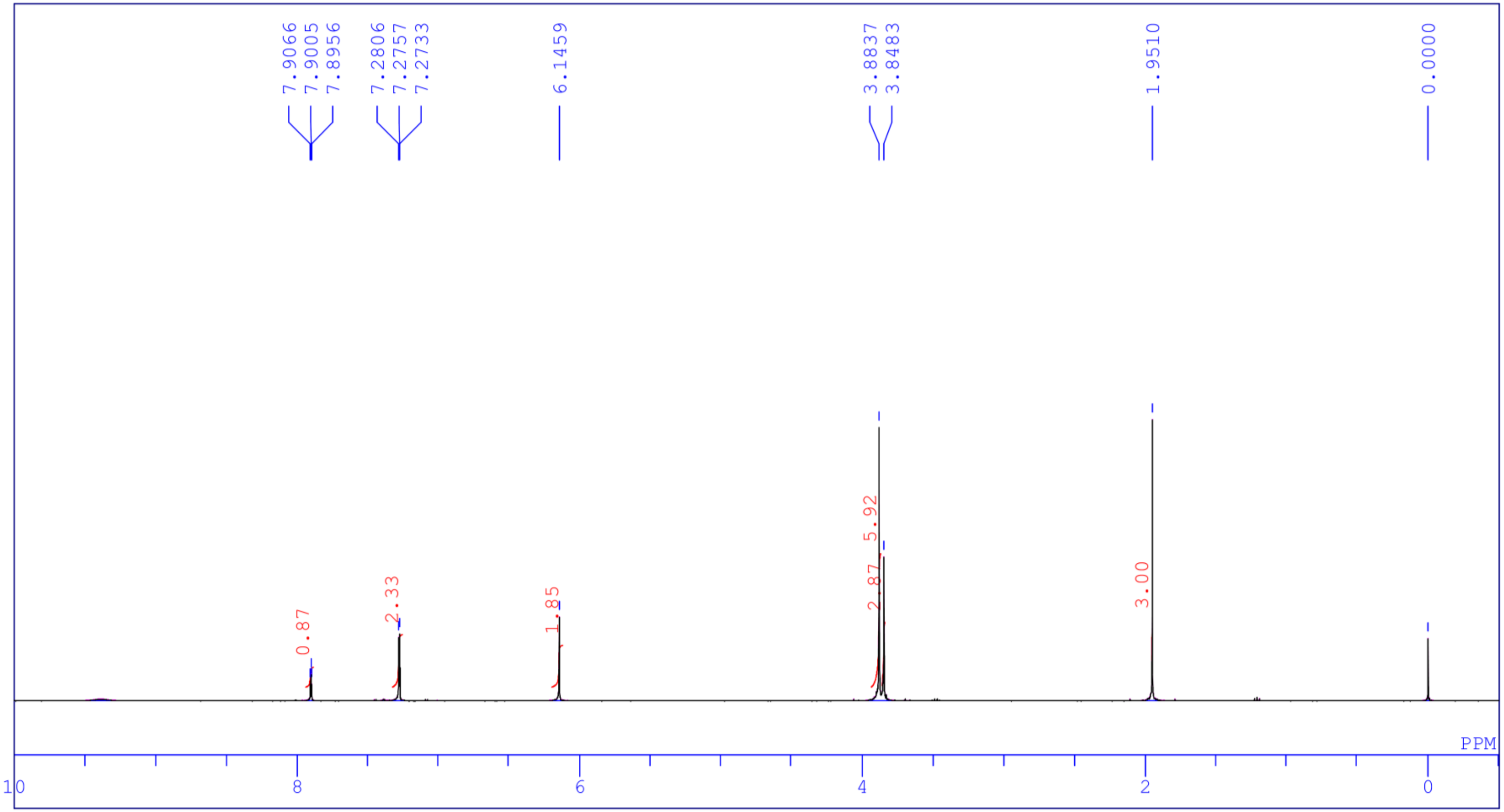

3-Thienyl(2,4,6-trimethoxyphenyl)iodonium acetate (4k)

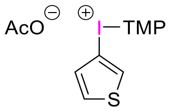

^{13}C NMR (100 MHz, $CDCl_3$)

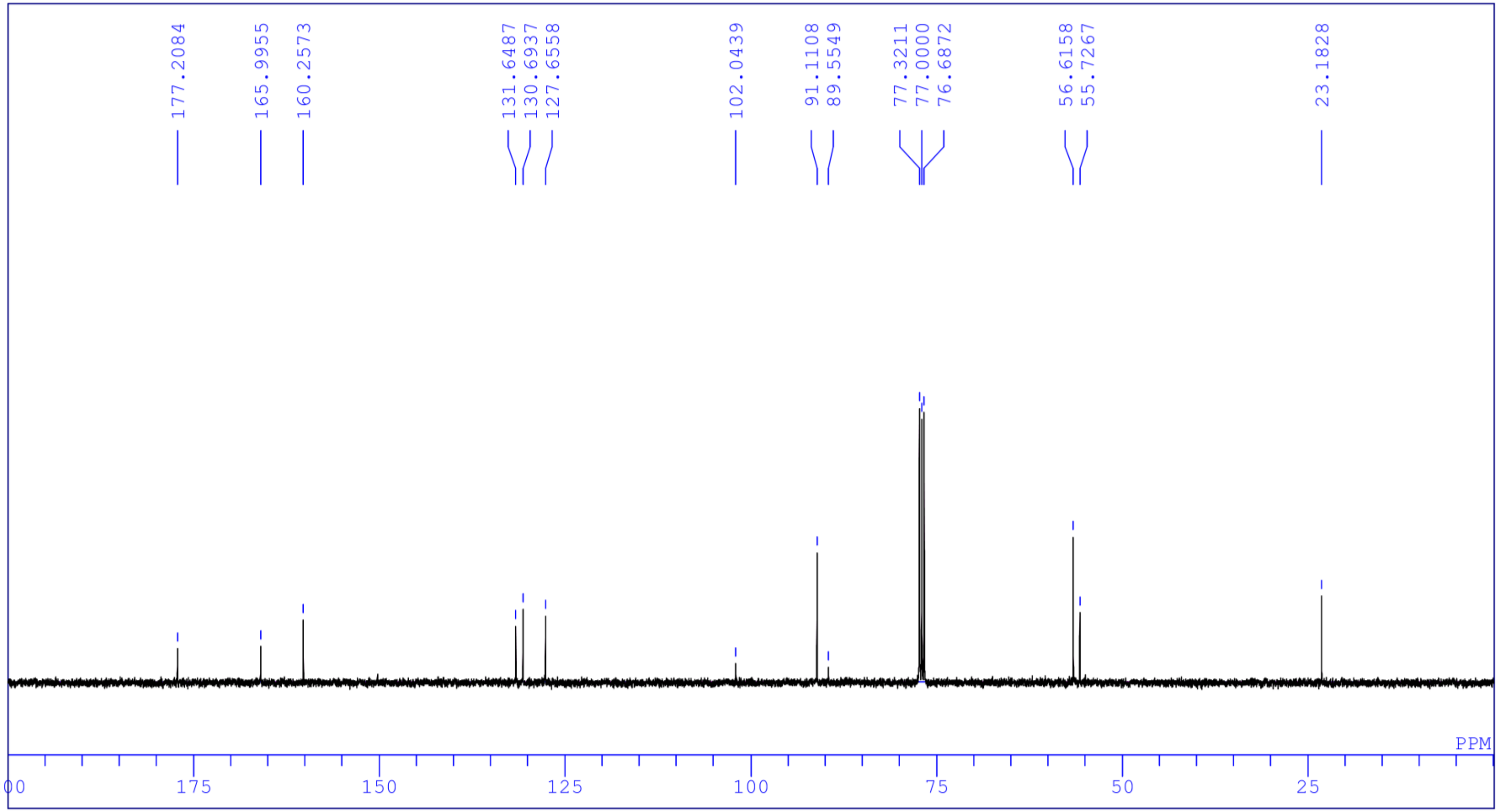

1,1'-Biphenyl-4,4'-diyl(2,4,6-trimethoxyphenyl)iodonium acetate (4l)

^{1}H NMR (400 MHz, CDCl₃)

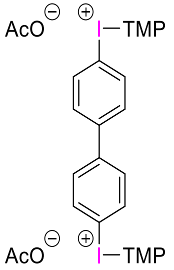

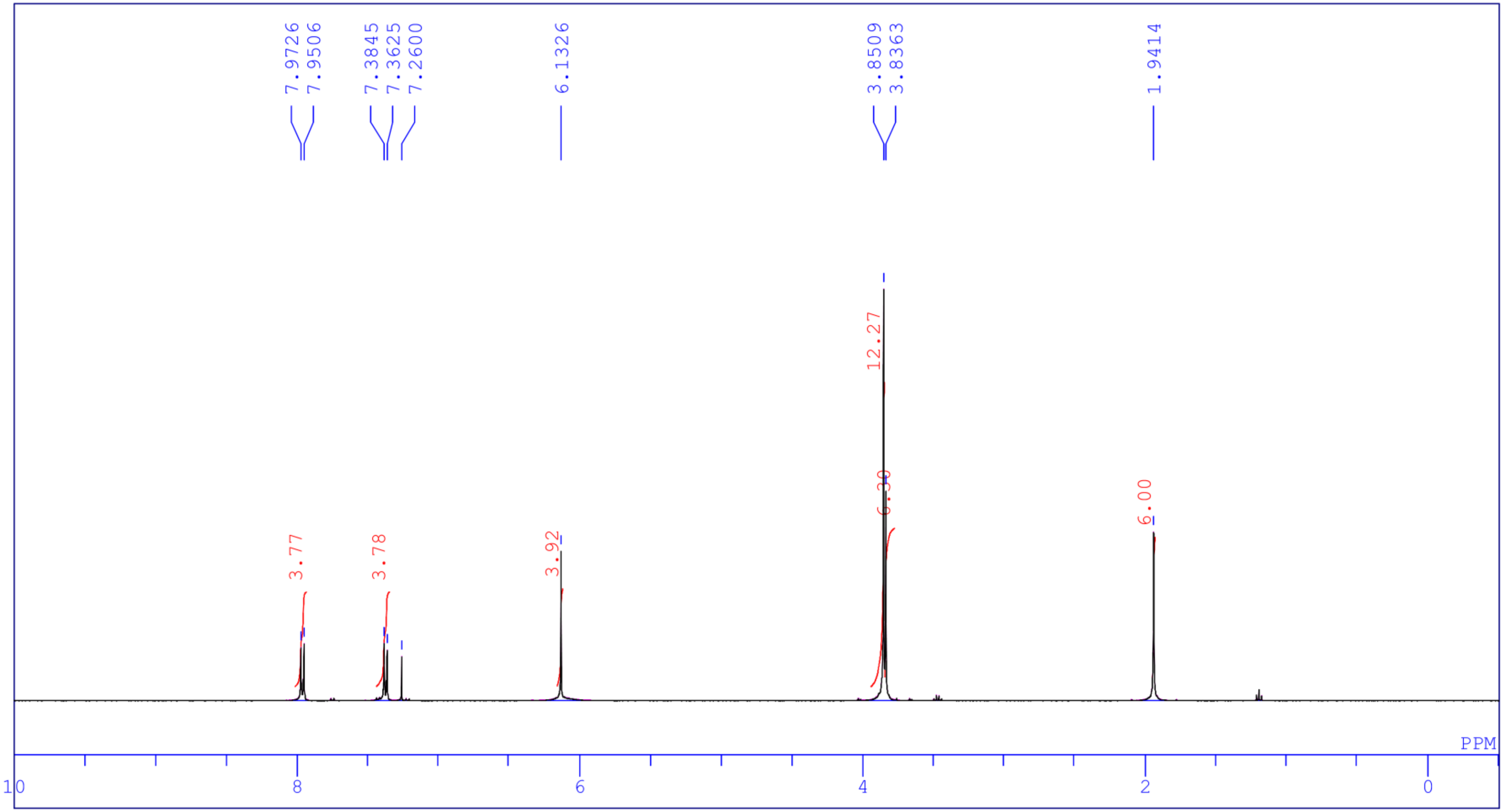

1,1'-Biphenyl-4,4'-diyl(2,4,6-trimethoxyphenyl)iodonium acetate (4l)

^{13}C NMR (100 MHz, CDCl3)

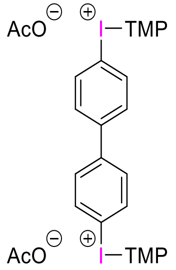

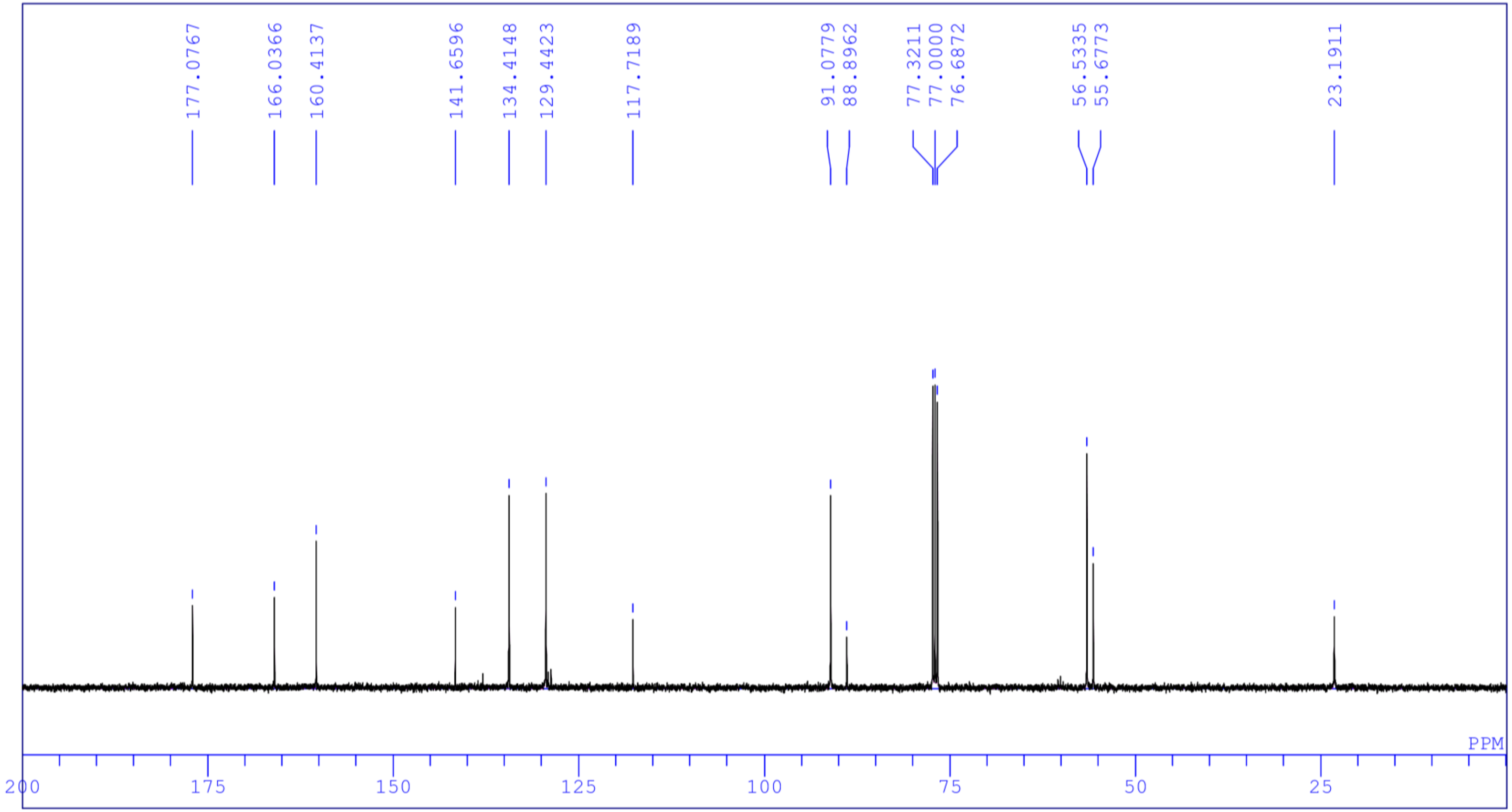

Phenyl(2,4,6-trimethoxyphenyl)iodonium trifluoroacetate (4m)

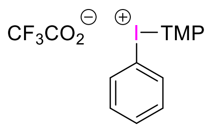

^{1}H NMR (400 MHz, DMSO-*d*6)

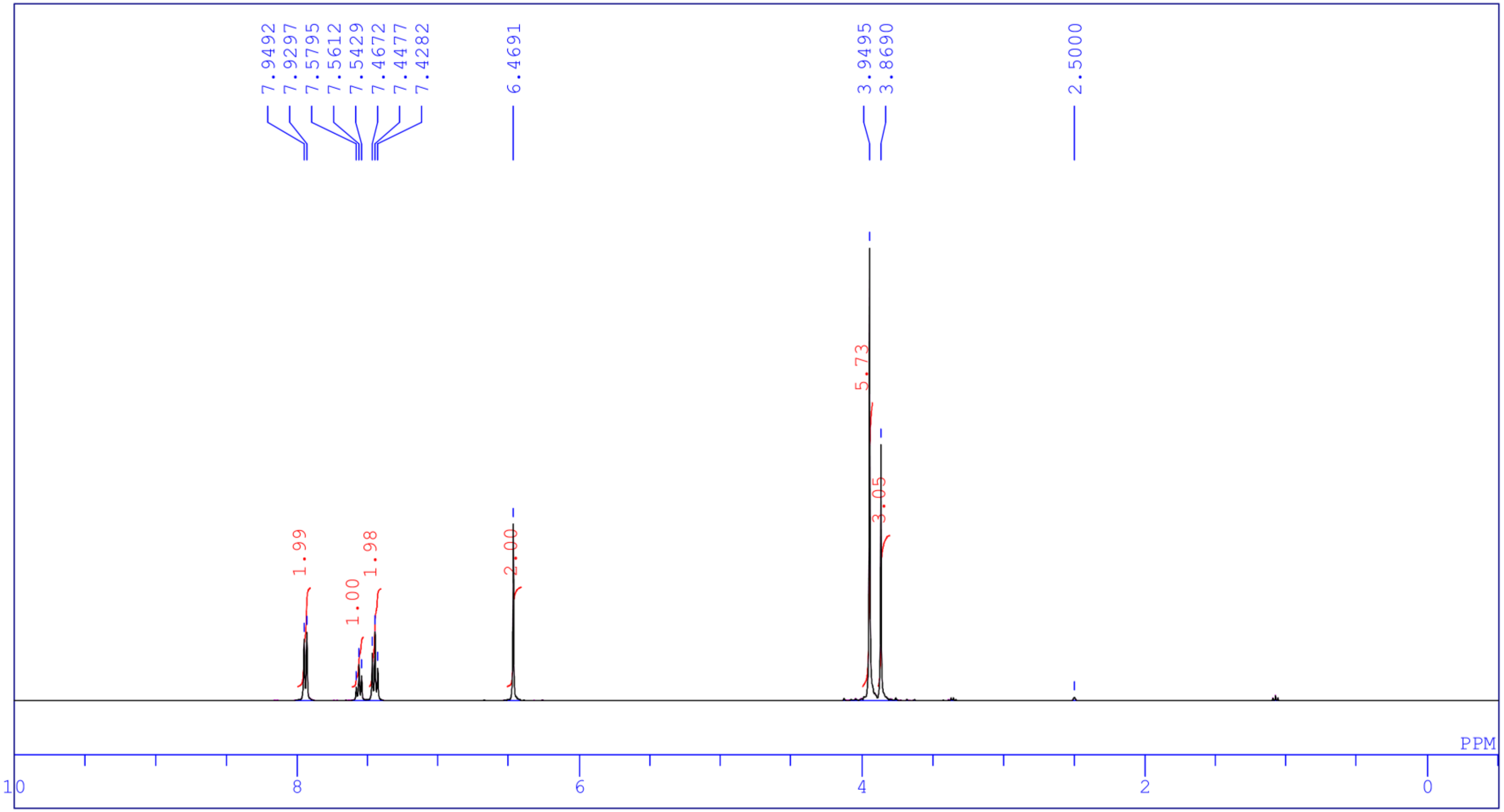

Phenyl(2,4,6-trimethoxyphenyl)iodonium trifluoroacetate (4m)

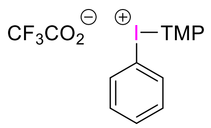

^{13}C NMR (100 MHz, DMSO-*d*6)

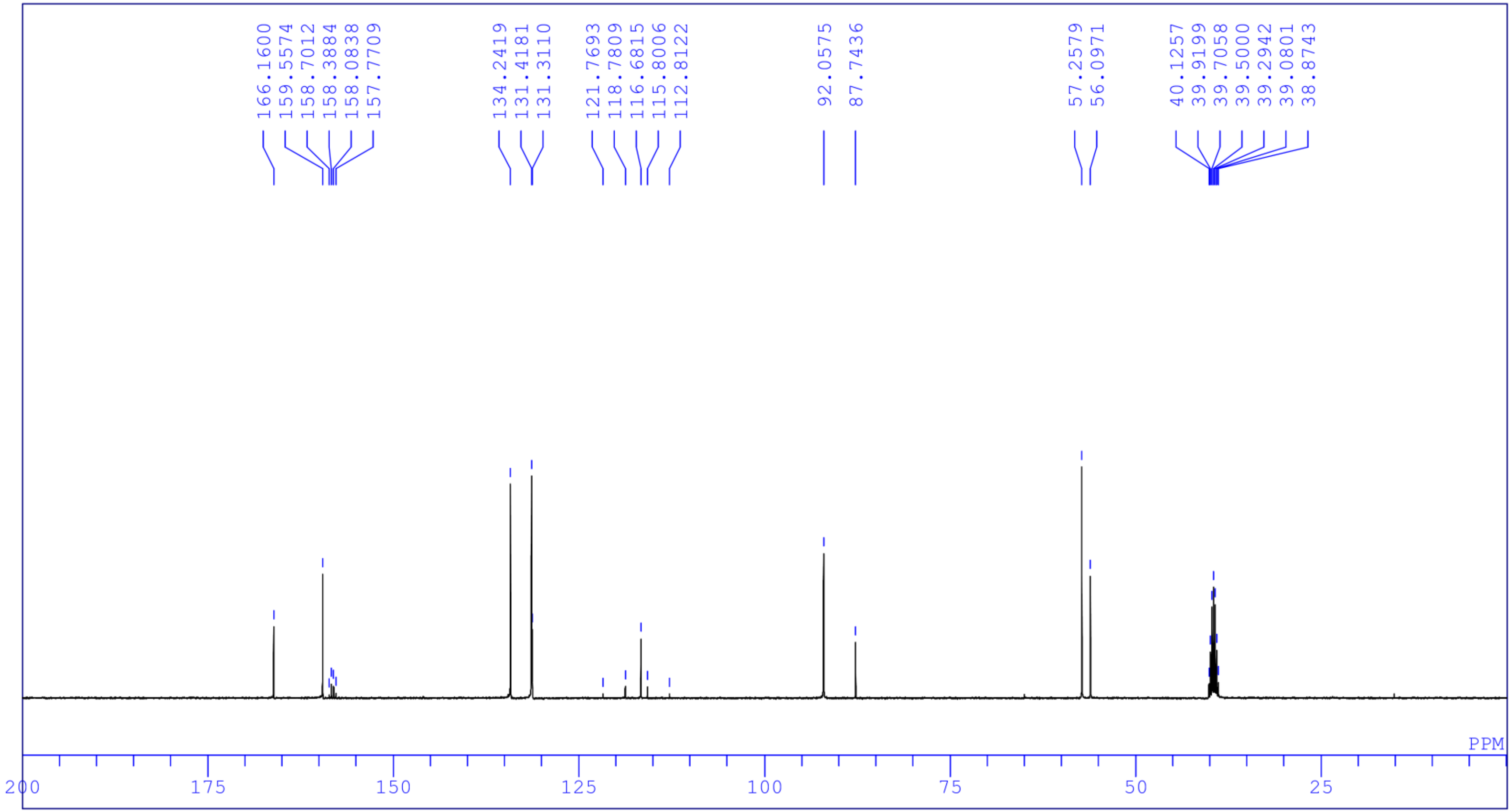

Phenyl(2,4,6-trimethoxyphenyl)iodonium trifluoroacetate (4m)

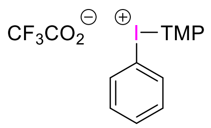

^{19}F NMR (376 MHz, DMSO-*d*6)

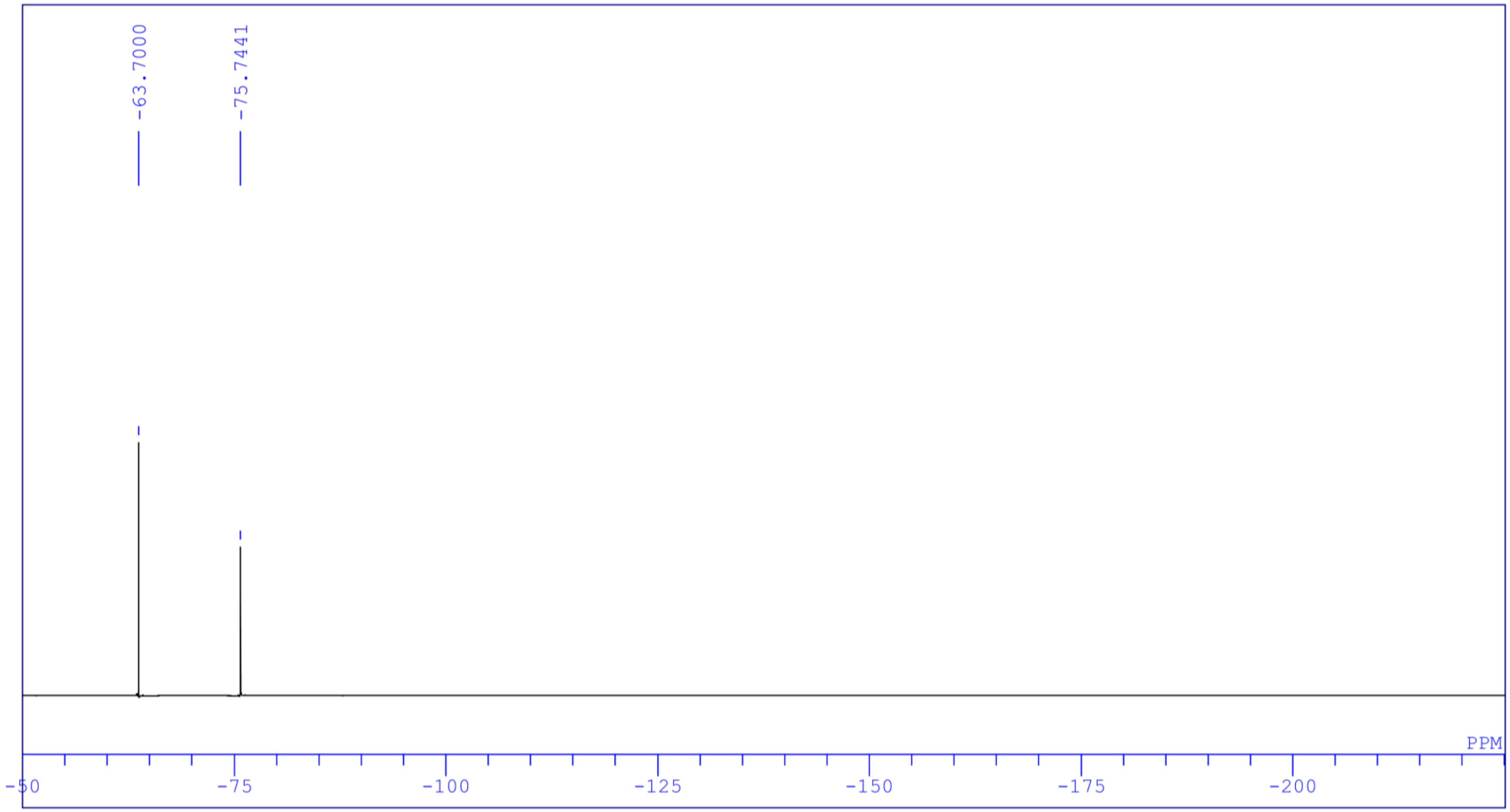

Phenyl(2,4,6-trimethoxyphenyl)iodonium benzoate (7aa)

^{1}H NMR (400 MHz, $CDCl_3$)

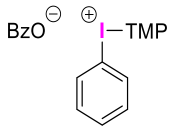

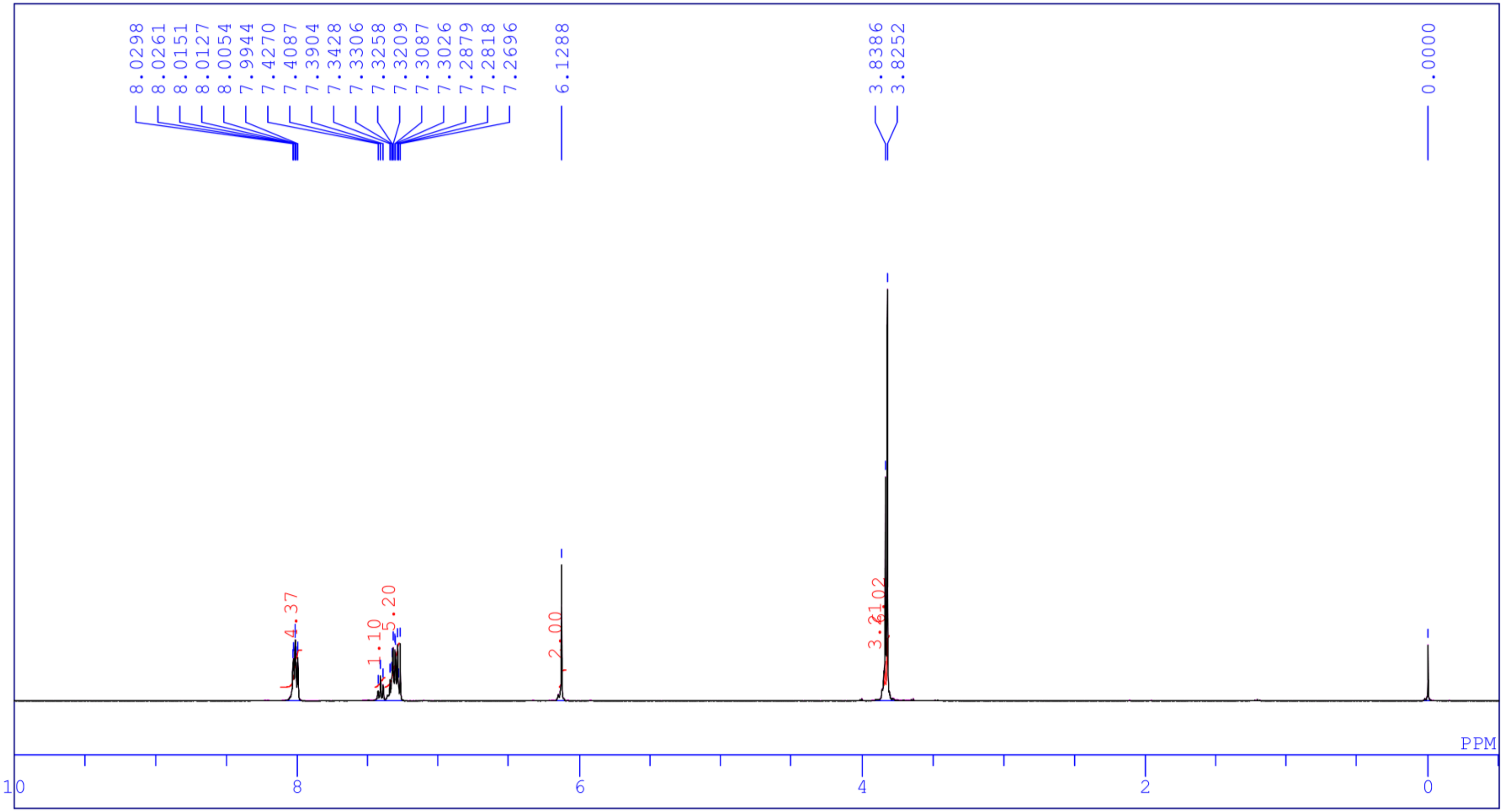

Phenyl(2,4,6-trimethoxyphenyl)iodonium benzoate (7aa)

^{13}C NMR (100 MHz, CDCl$_3$)

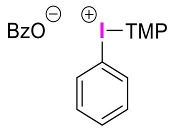

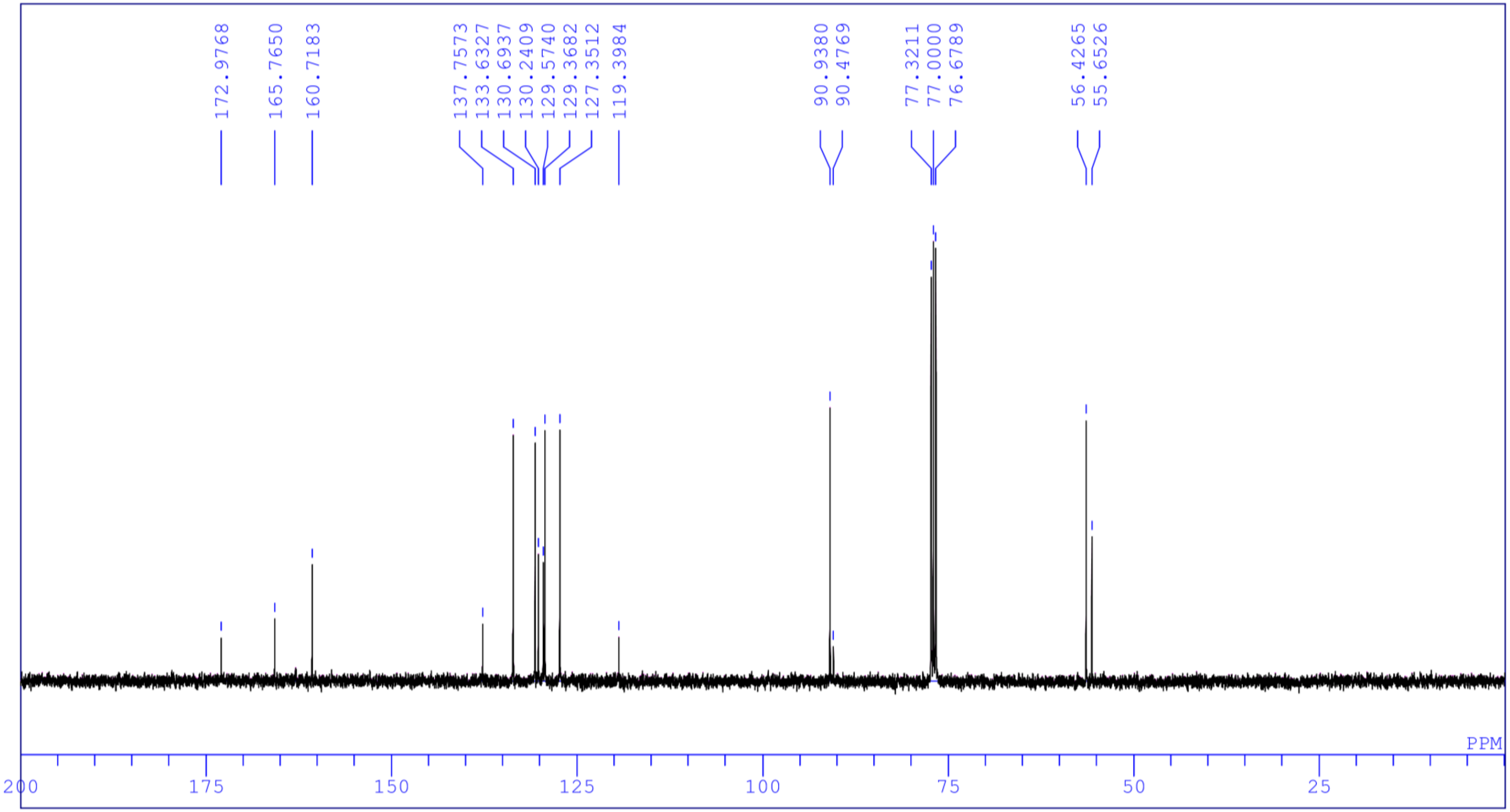

Phenyl(2,4,6-trimethoxyphenyl)iodonium 2-iodobenzoate (7ab)

^{1}H NMR (400 MHz, CDCl3)

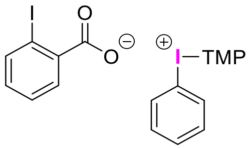

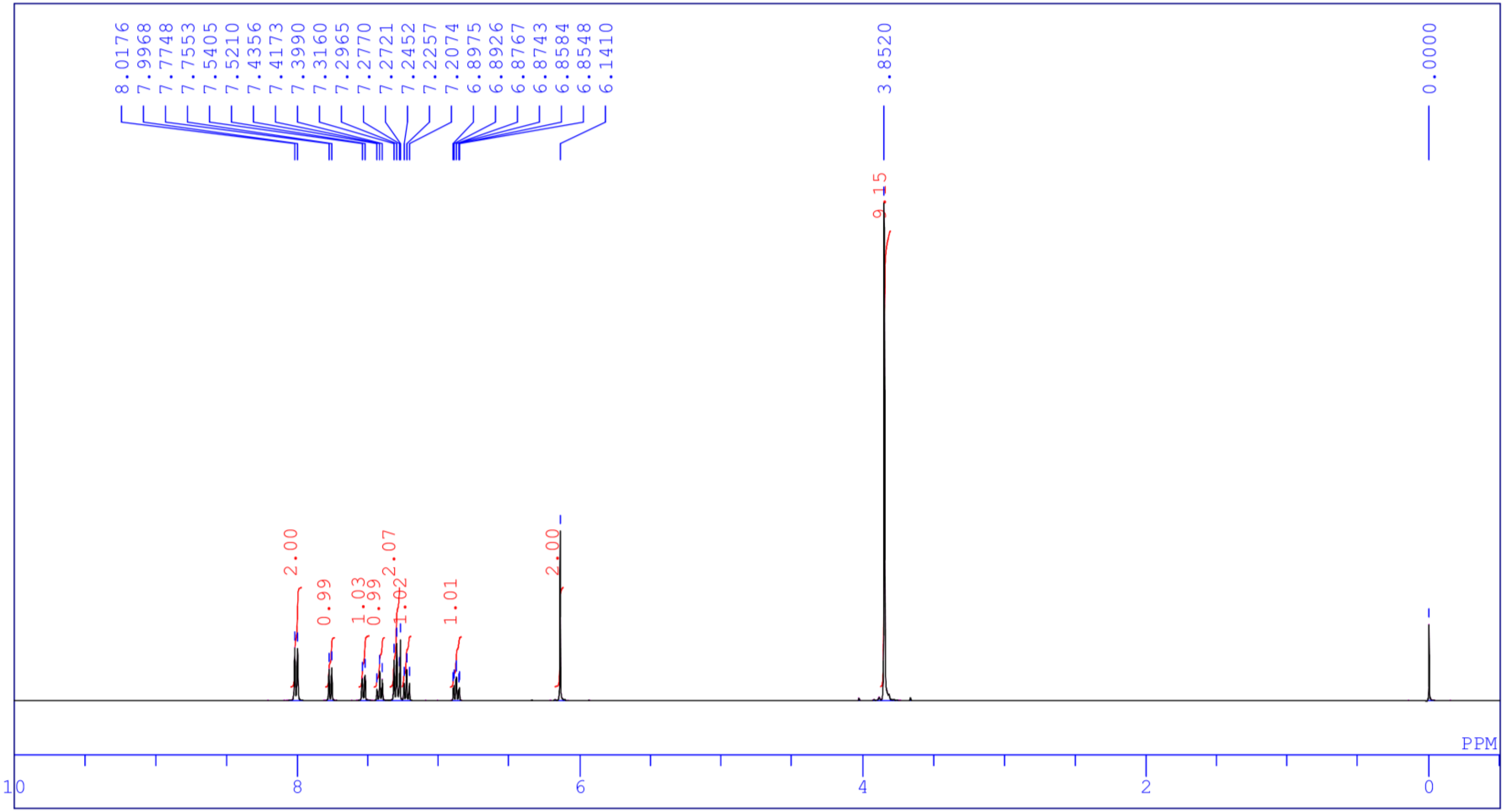

Phenyl(2,4,6-trimethoxyphenyl)iodonium 2-iodobenzoate (7ab)

^{13}C NMR (100 MHz, $CDCl_3$)

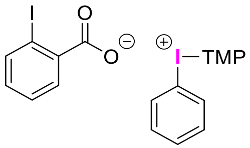

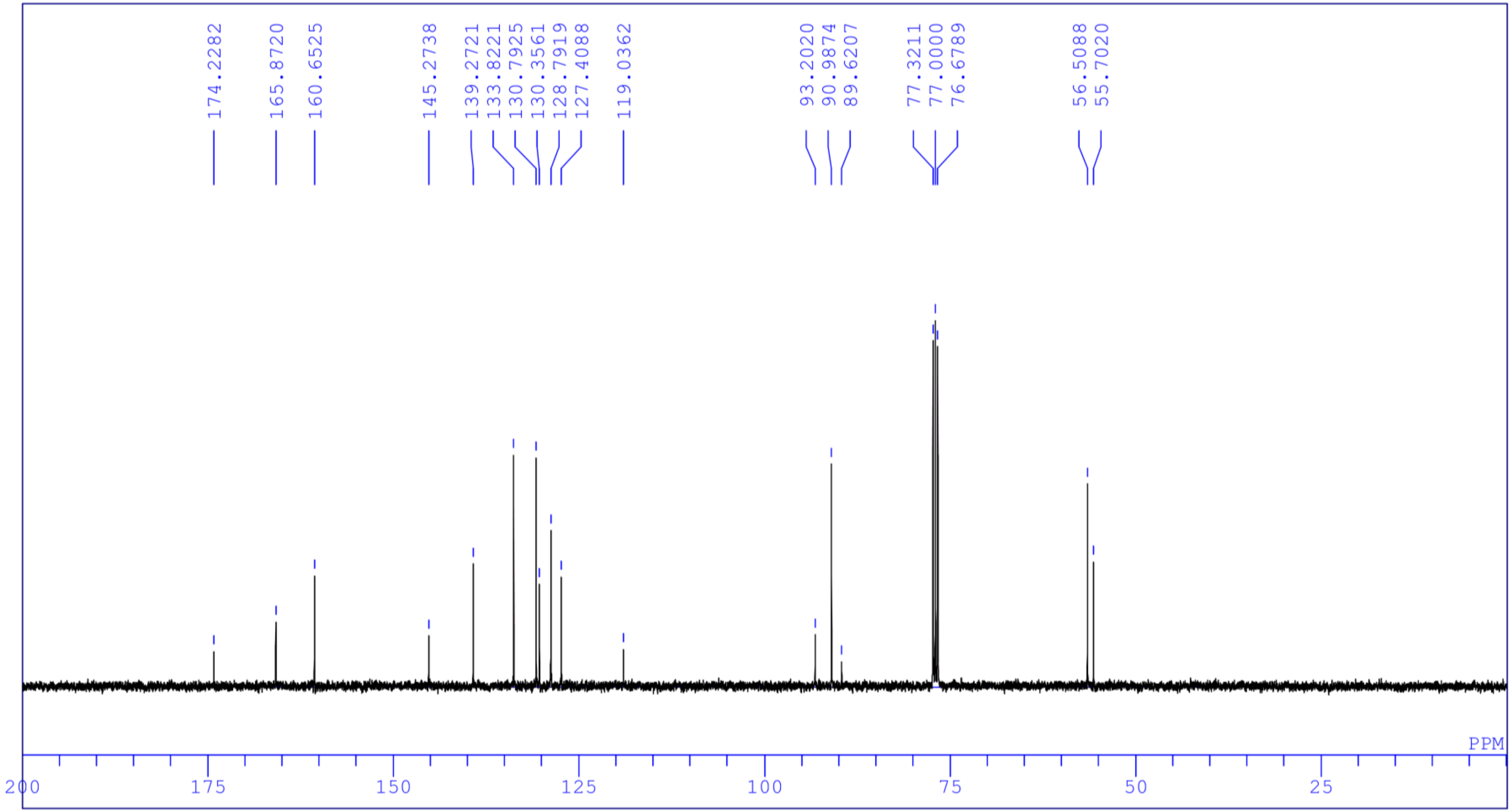

Phenyl(2,4,6-trimethoxyphenyl)iodonium pyridine-3-carboxylate (7ac)

^{1}H NMR (400 MHz, $CDCl_3$)

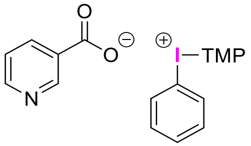

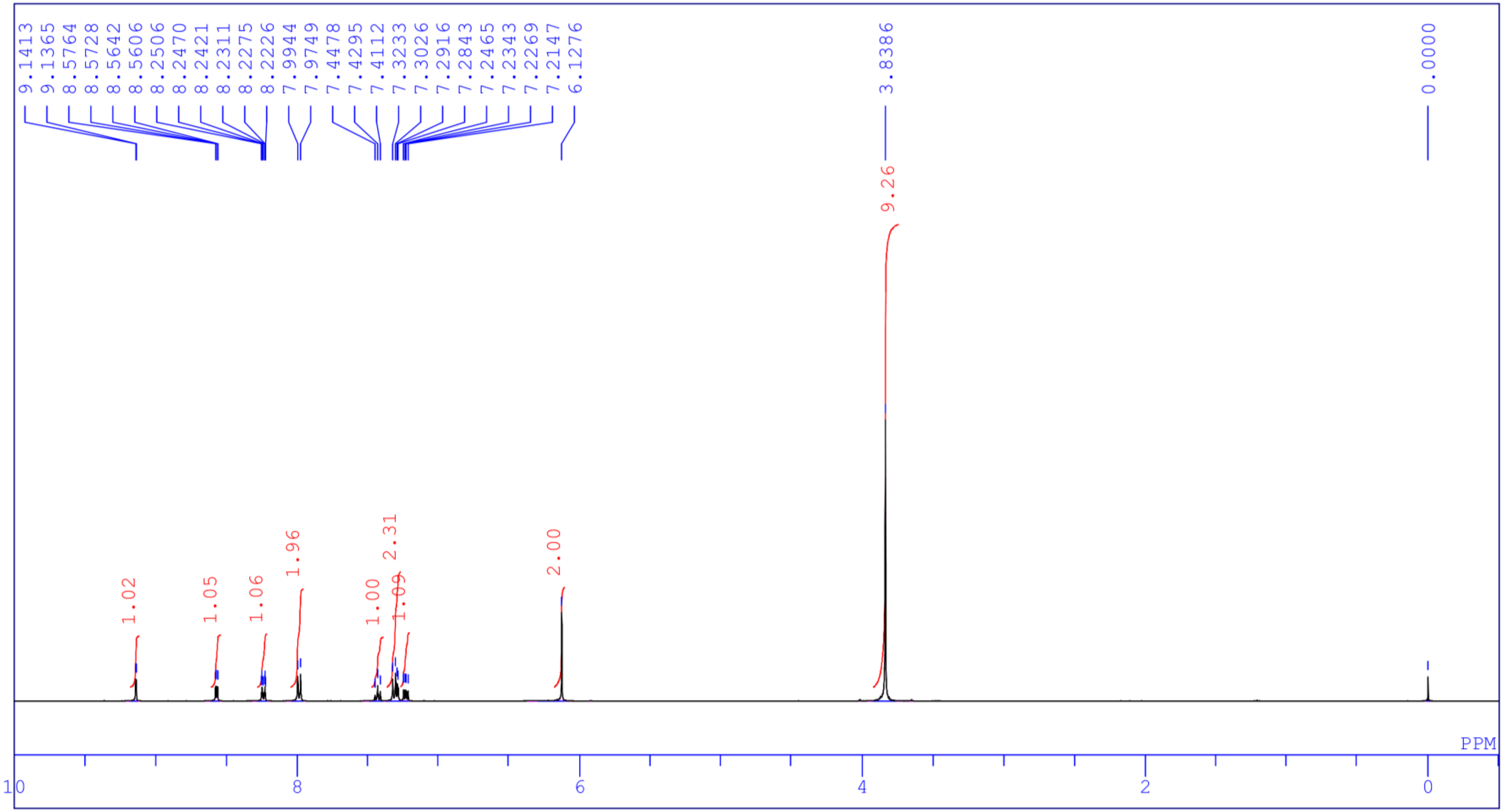

Phenyl(2,4,6-trimethoxyphenyl)iodonium pyridine-3-carboxylate (7ac)

^{13}C NMR (100 MHz, $CDCl_3$)

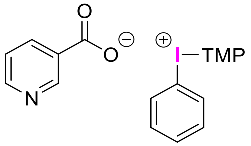

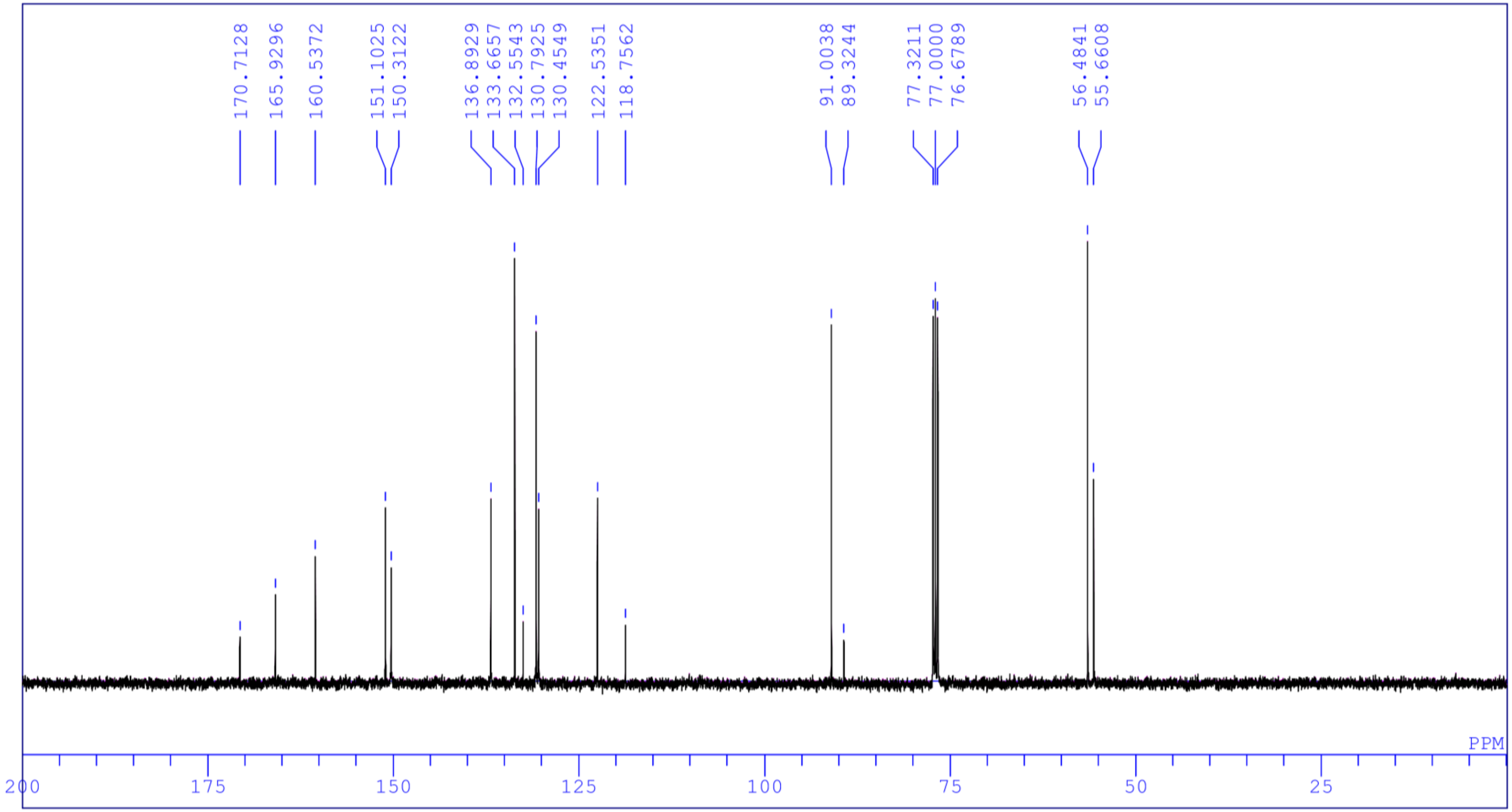

Phenyl(2,4,6-trimethoxyphenyl)iodonium thiophene-3-carboxylate (7ad)

^{1}H NMR (400 MHz, $CDCl_3$)

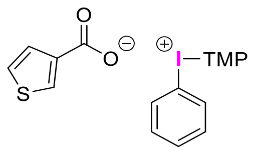

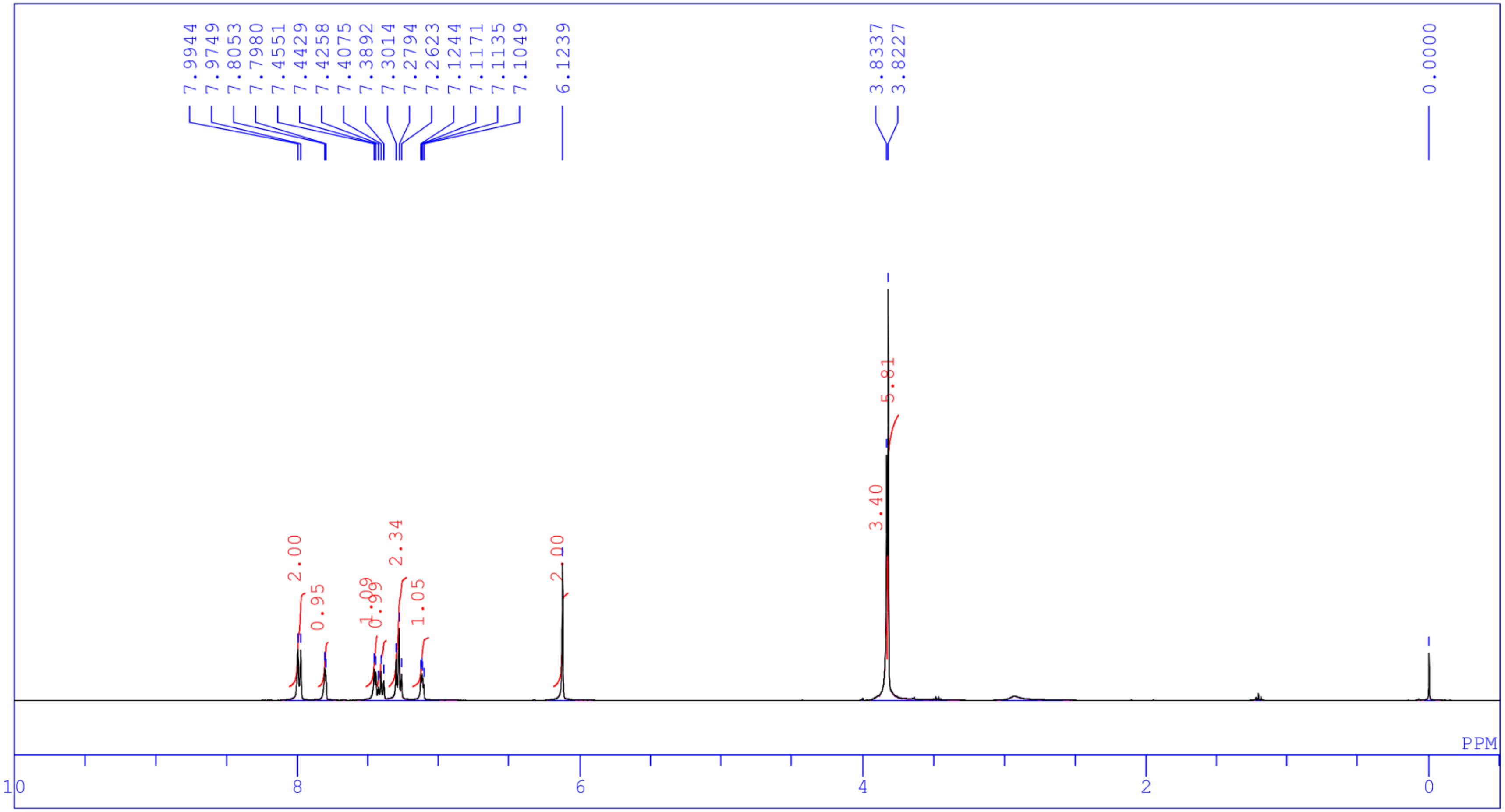

Phenyl(2,4,6-trimethoxyphenyl)iodonium thiophene-3-carboxylate (7ad)

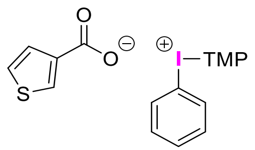

^{13}C NMR (100 MHz, CDCl3)

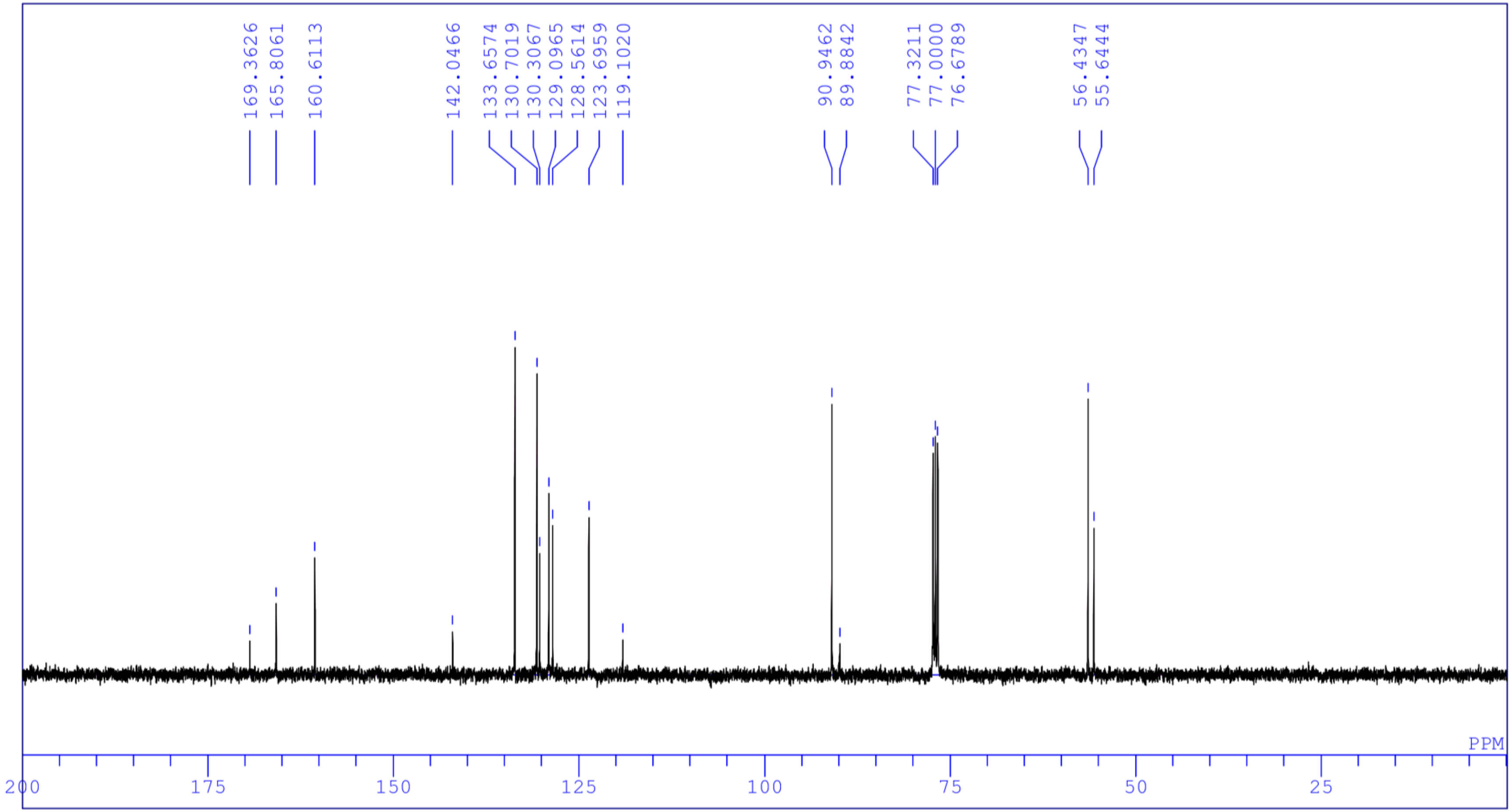

Phenyl(2,4,6-trimethoxyphenyl)iodonium 2,2-dimethylpropanoate (7af)

^{1}H NMR (400 MHz, $CDCl_3$)

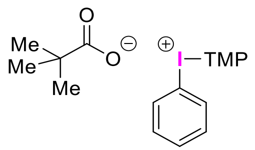

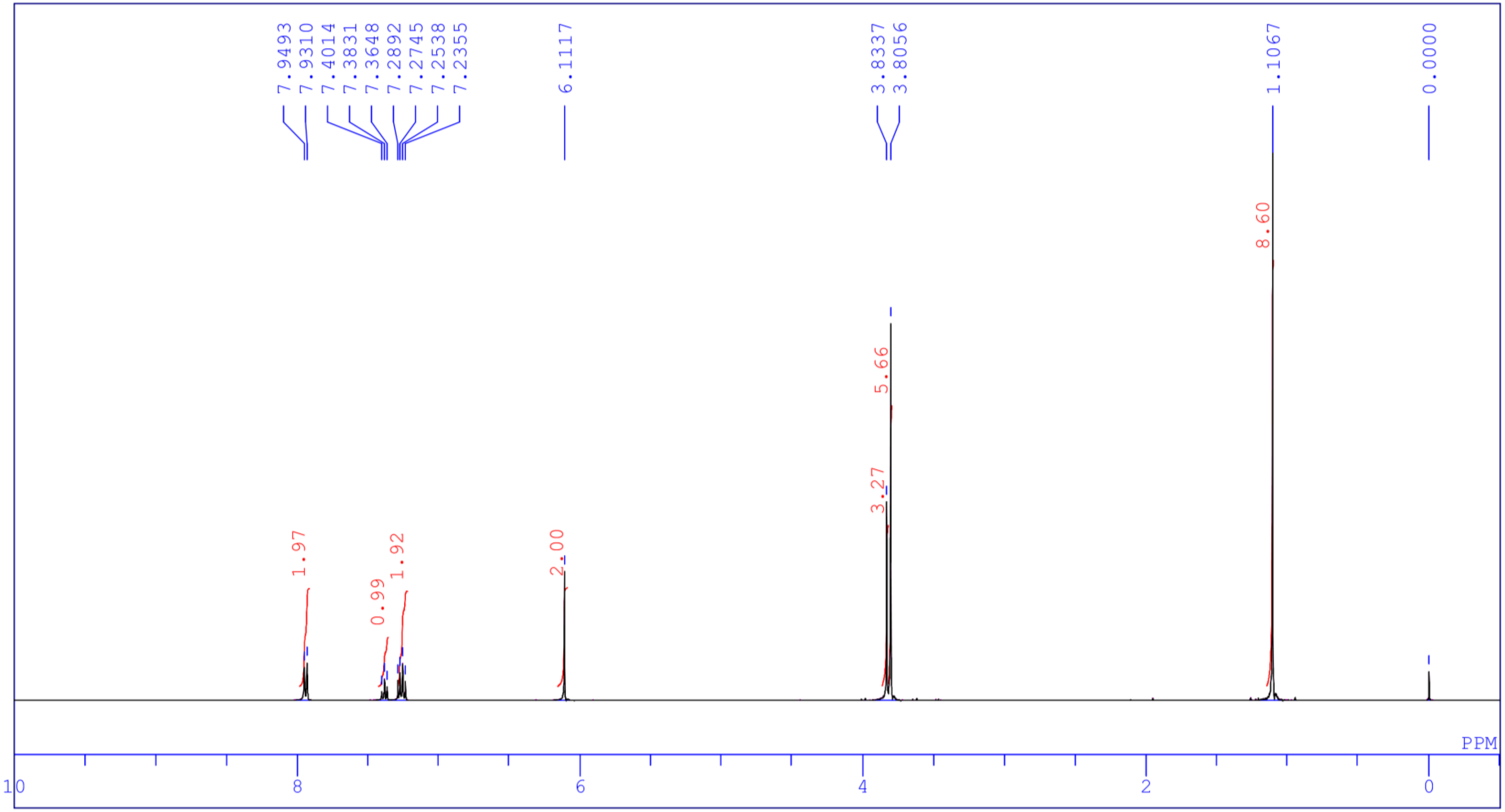

Phenyl(2,4,6-trimethoxyphenyl)iodonium 2,2-dimethylpropanoate (7af)

^{13}C NMR (100 MHz, $CDCl_3$)

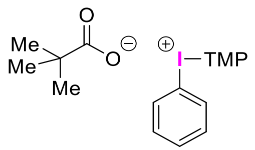

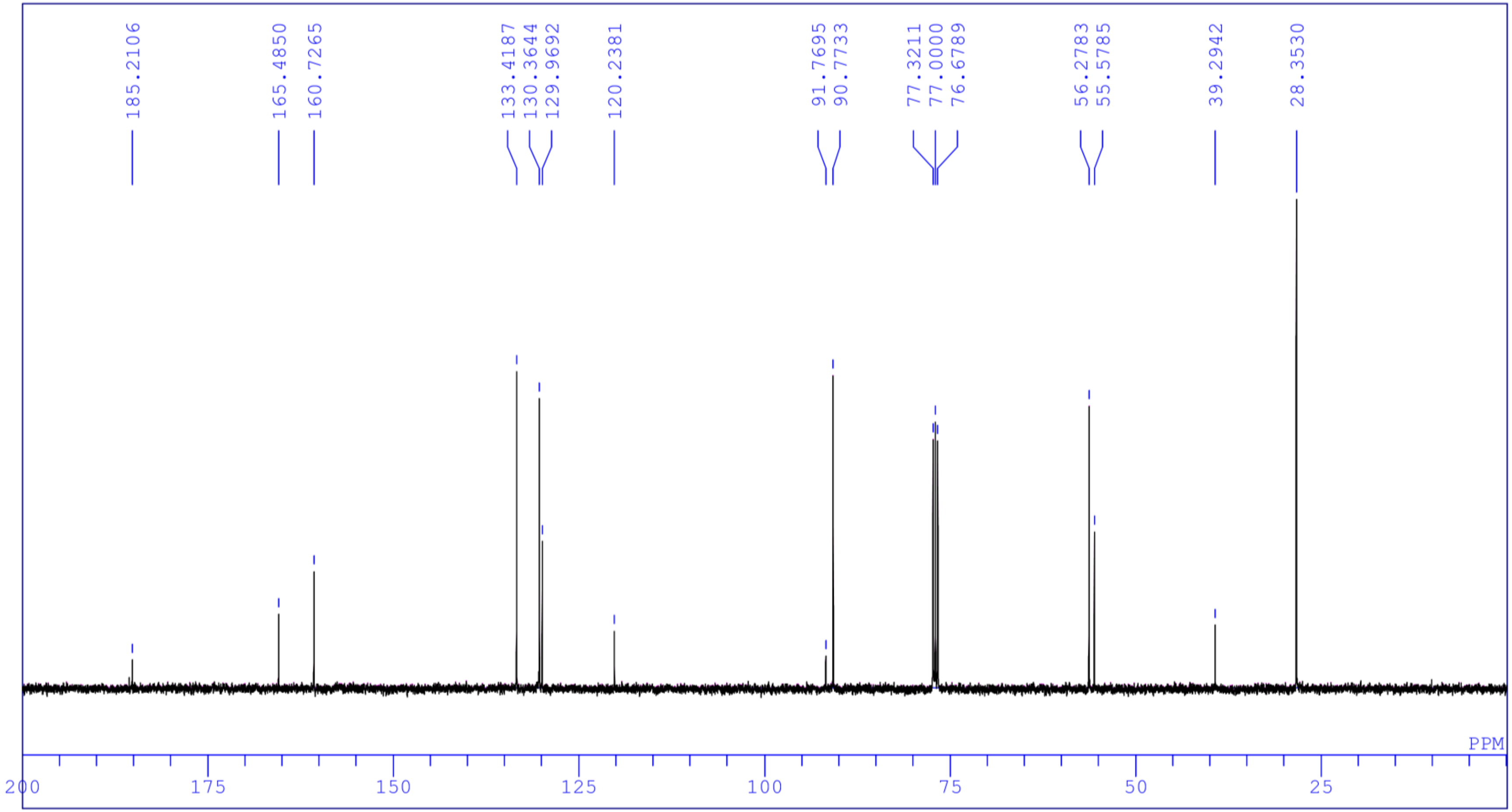

Phenyl(2,4,6-trimethoxyphenyl)iodonium cyclohexanecarboxylate (7ag)

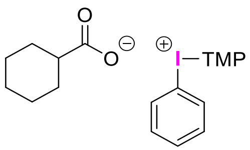

^{1}H NMR (400 MHz, $CDCl_3$)

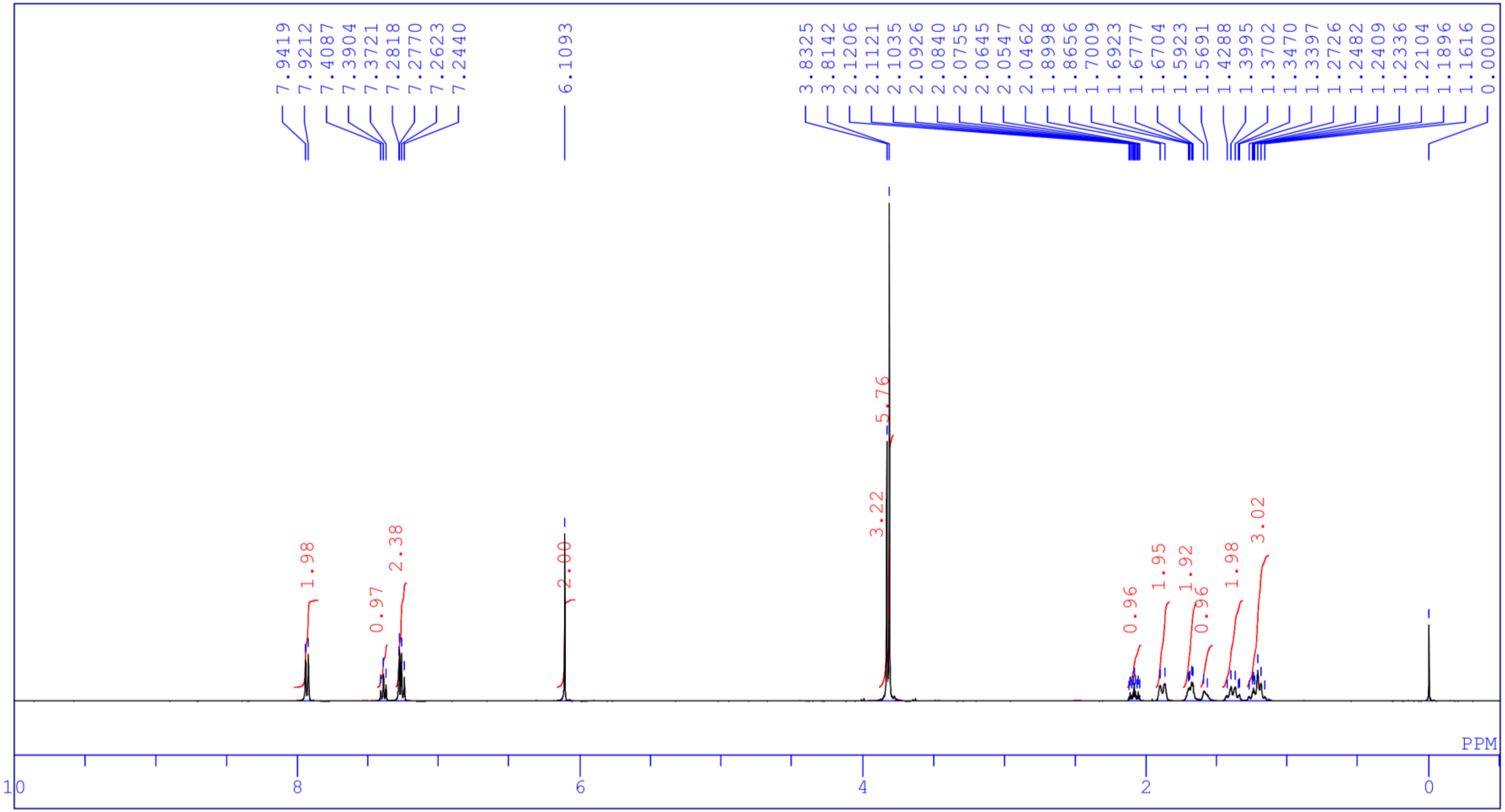

Phenyl(2,4,6-trimethoxyphenyl)iodonium cyclohexanecarboxylate (7ag)

^{13}C NMR (100 MHz, $CDCl_3$)

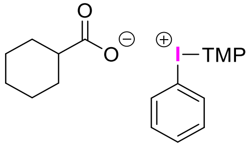

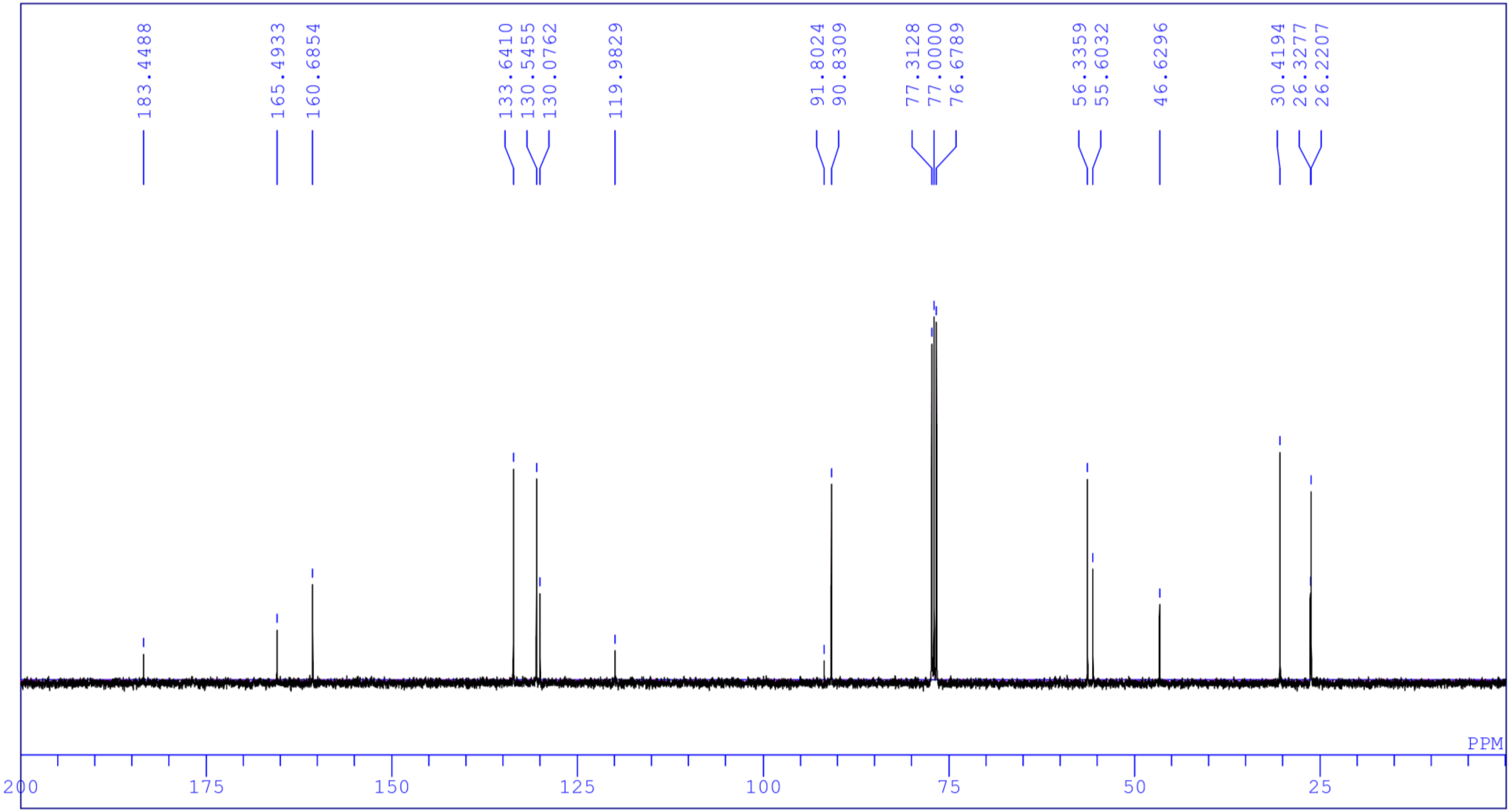

Phenyl(2,4,6-trimethoxyphenyl)iodonium 2-cyanoacetate (7ah)

^{1}H NMR (400 MHz, $CDCl_3$)

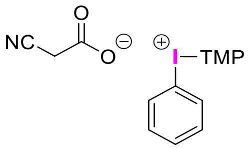

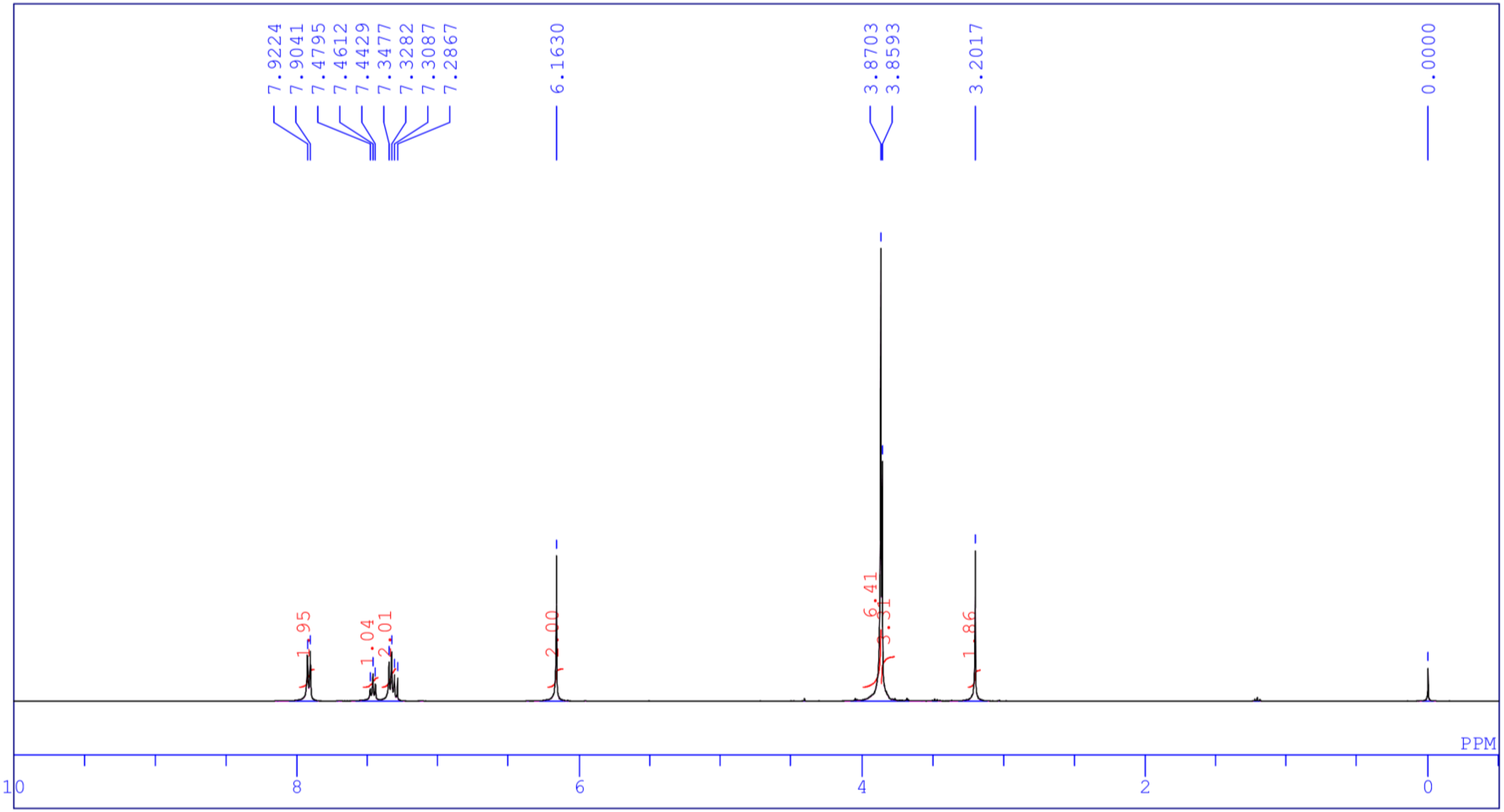

Phenyl(2,4,6-trimethoxyphenyl)iodonium 2-cyanoacetate (7ah)

^{13}C NMR (100 MHz, $CDCl_3$)

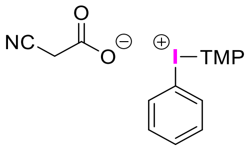

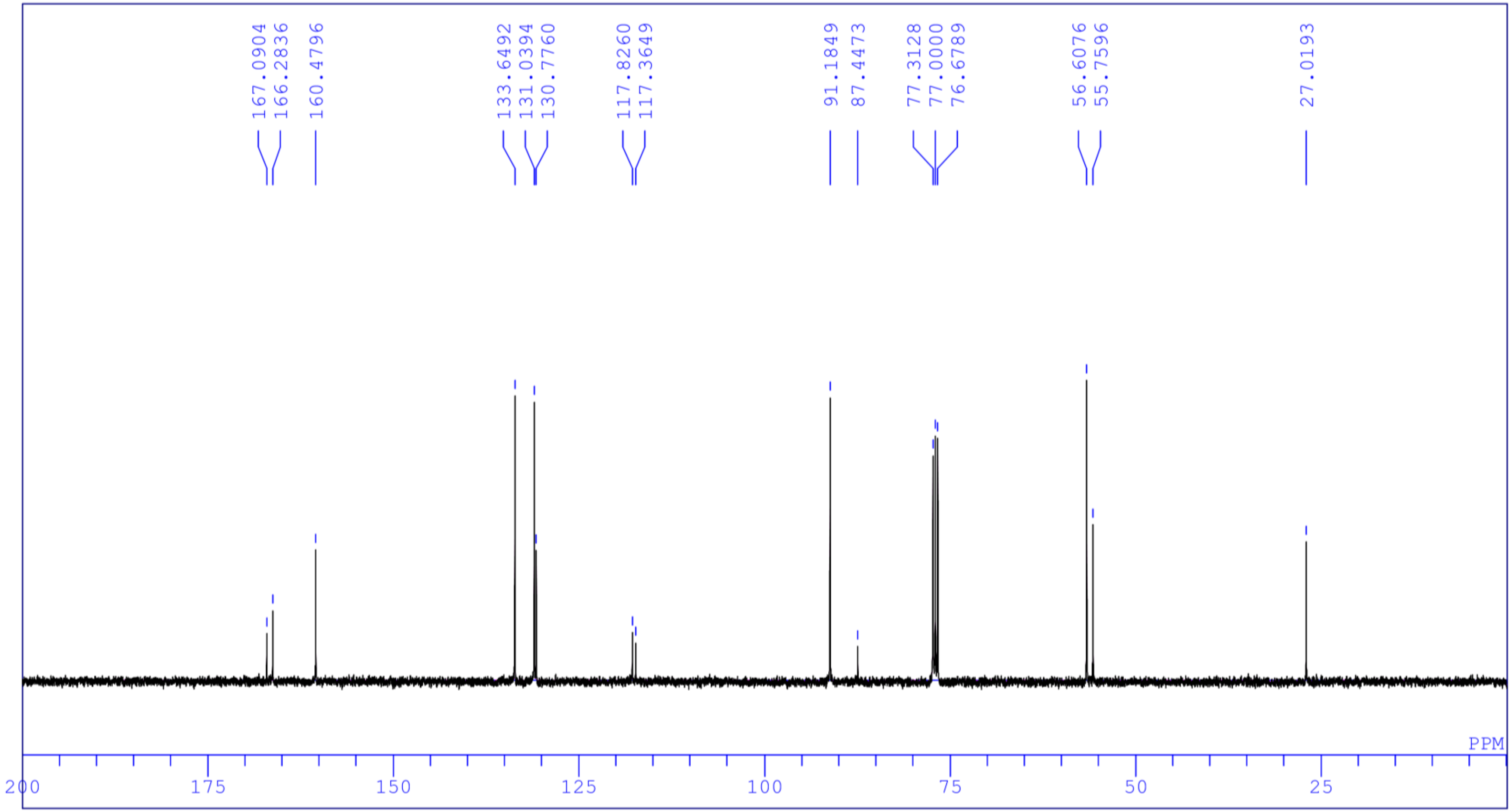

Phenyl(2,4,6-trimethoxyphenyl)iodonium 2-(1,3-dimethyl-2,6-dioxo-1,2,3,6-tetrahydro-*7H*-purin-7-yl)acetate (7ai)

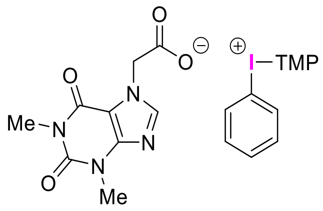

^{1}H NMR (400 MHz, CDCl$_3$)

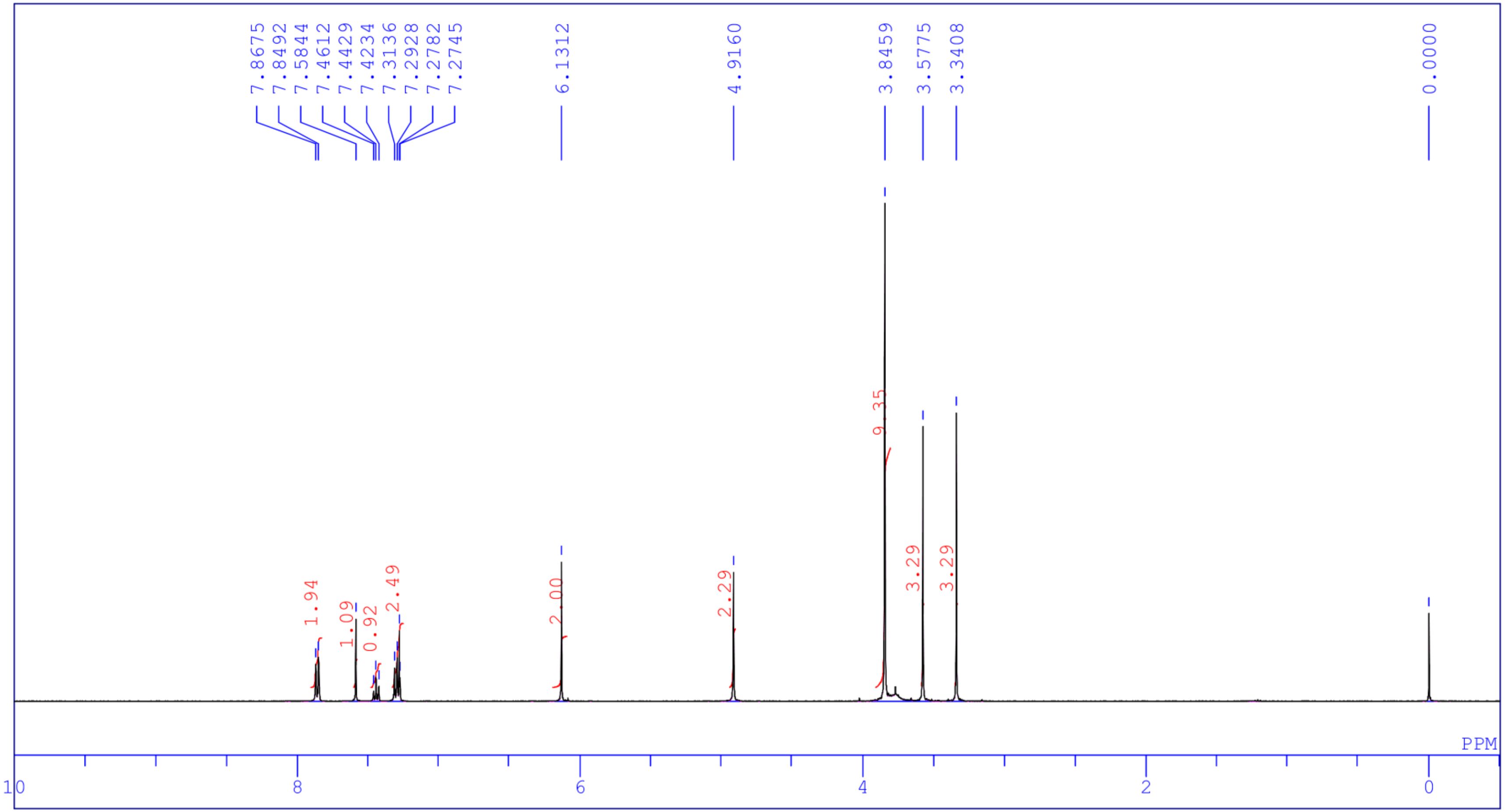

Phenyl(2,4,6-trimethoxyphenyl)iodonium 2-(1,3-dimethyl-2,6-dioxo-1,2,3,6-tetrahydro-*7H*-purin-7-yl)acetate (7ai)

^{13}C NMR (100 MHz, CDCl$_3$)

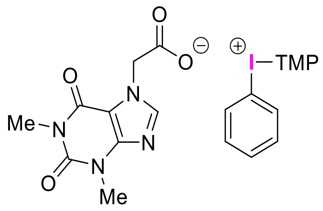

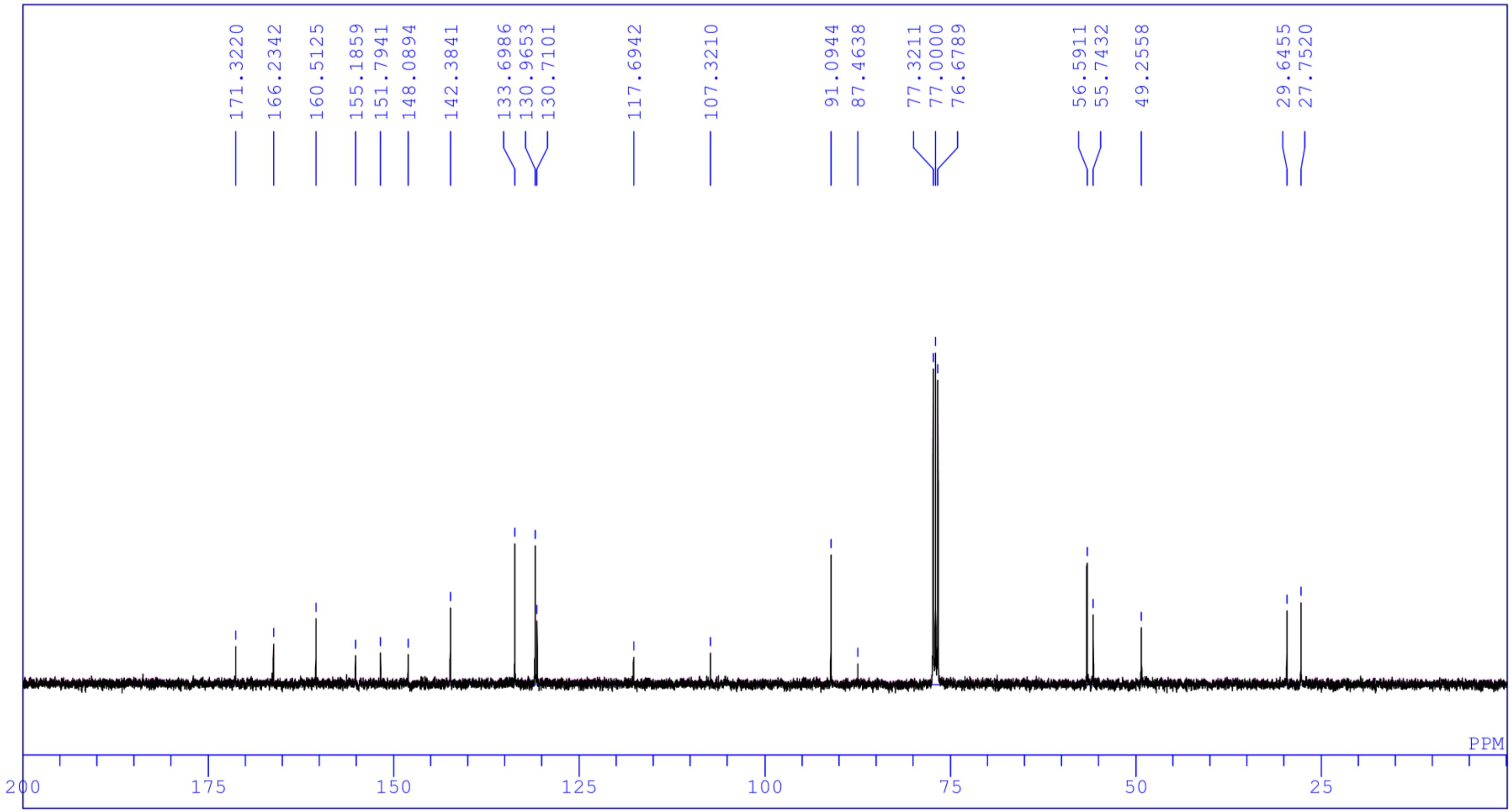

Phenyl(2,4,6-trimethoxyphenyl)iodonium 7-methoxycoumarin-3-carboxylate (7aj)

^{1}H NMR (400 MHz, CDCl₃)

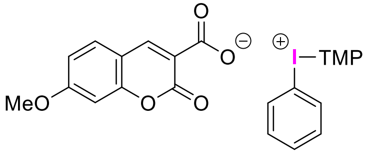

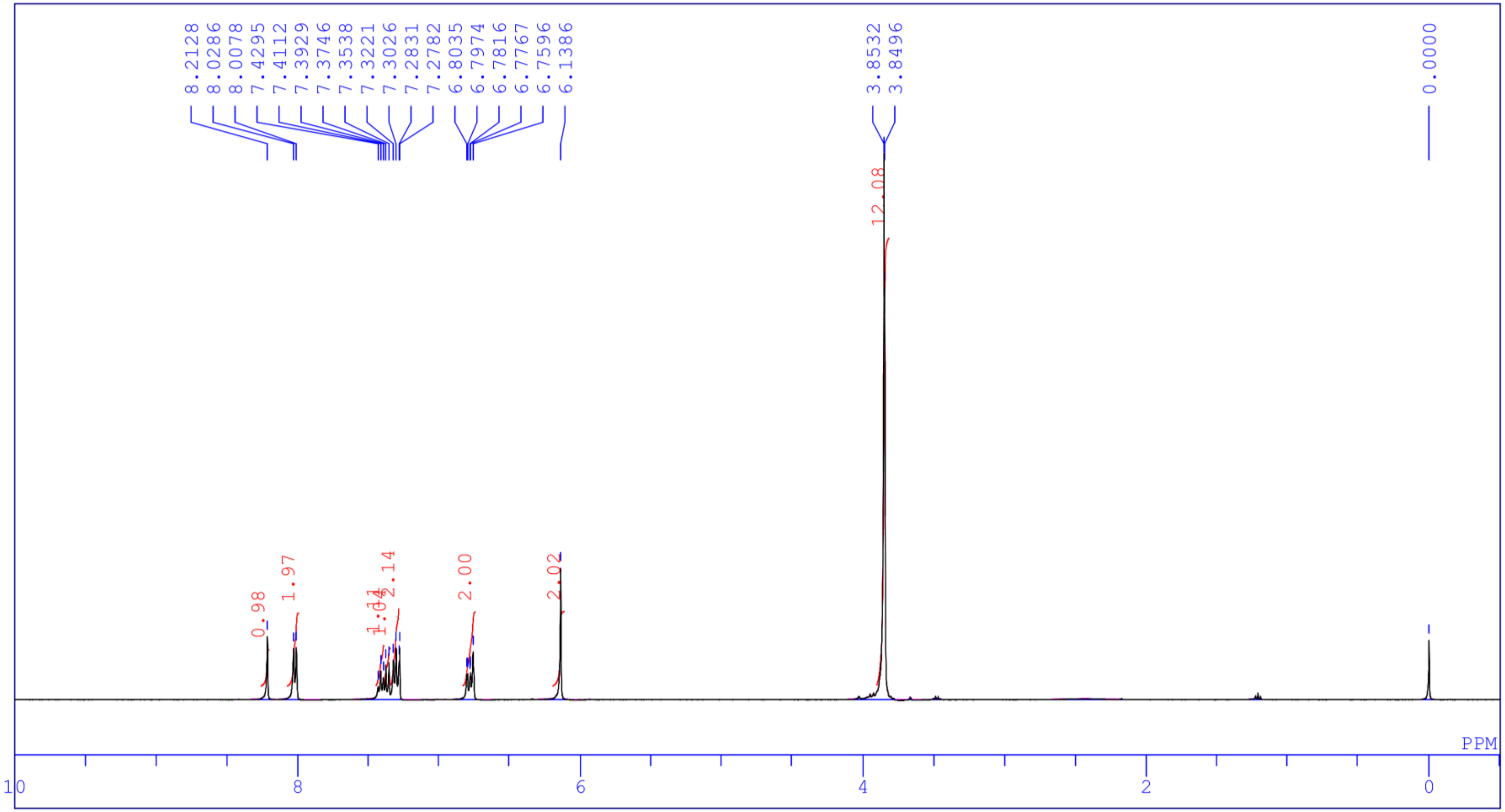

Phenyl(2,4,6-trimethoxyphenyl)iodonium 7-methoxycoumarin-3-carboxylate (7aj)

^{13}C NMR (100 MHz, CDCl₃)

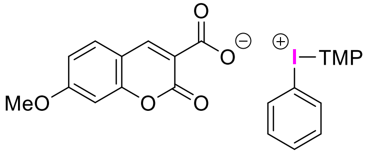

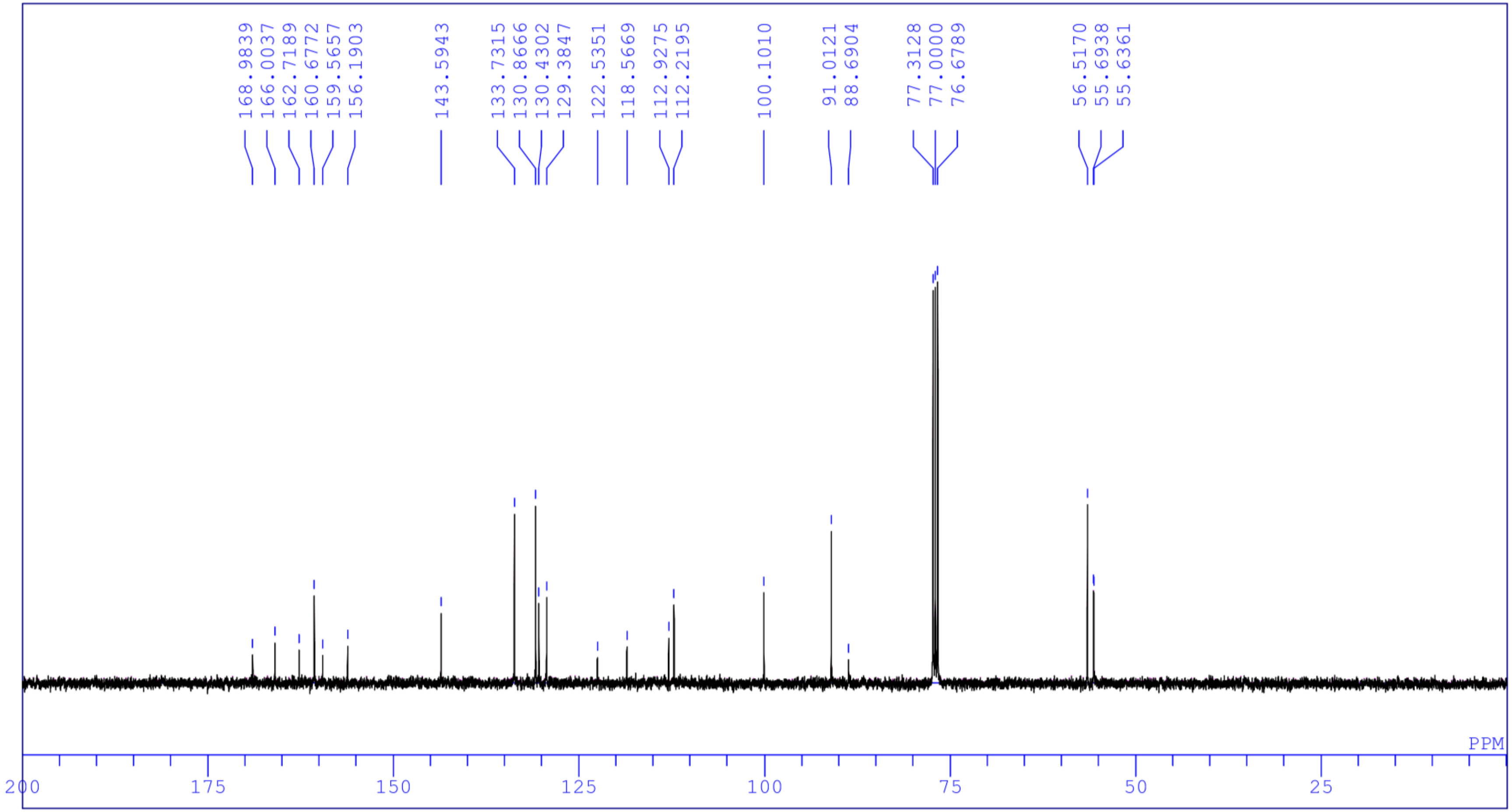

4-Methylphenyl(2,4,6-trimethoxyphenyl)iodonium benzoate (7ba)

^{1}H NMR (400 MHz, CDCl3)

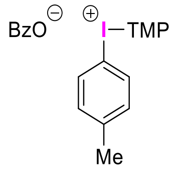

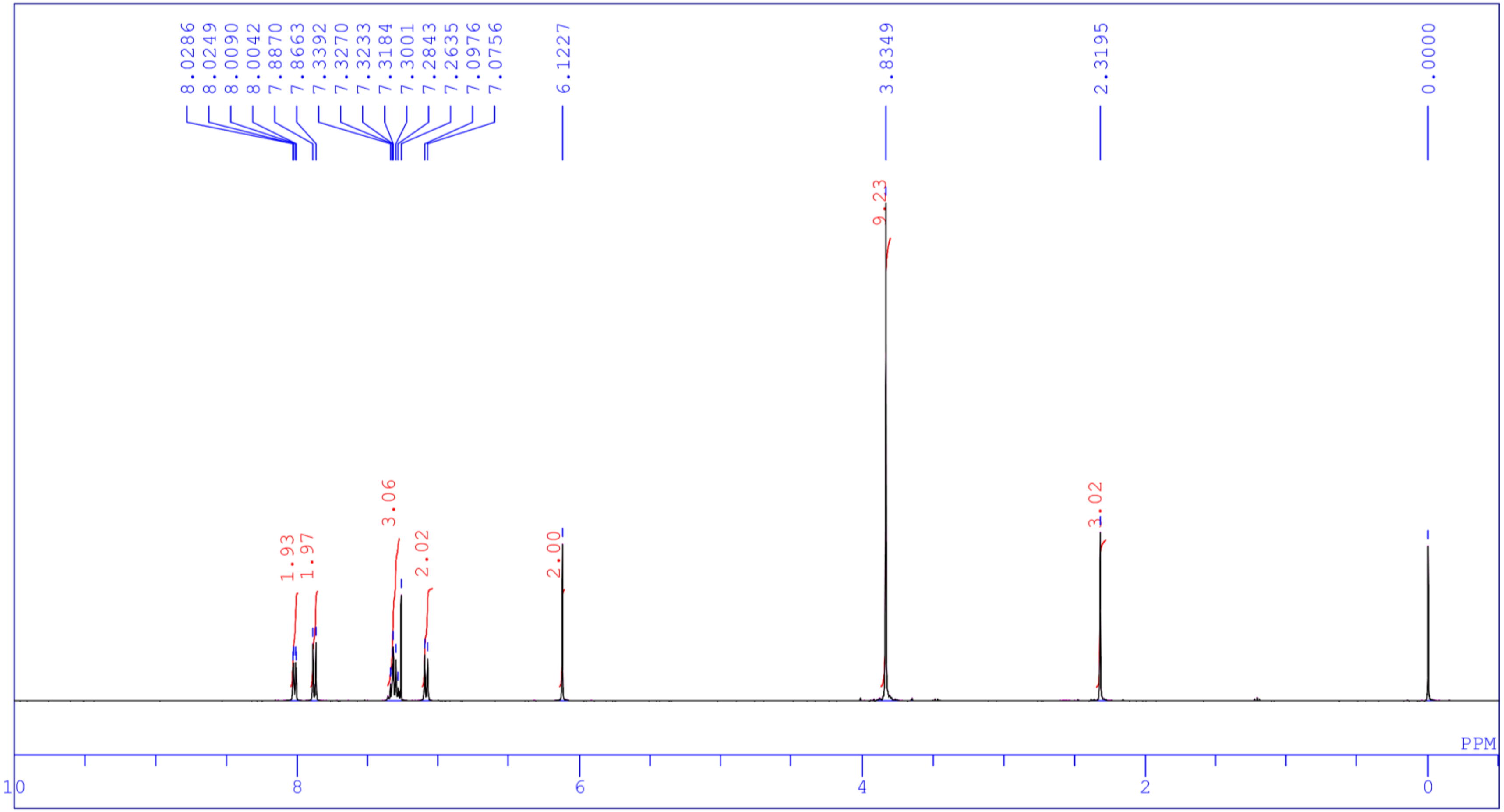

4-Methylphenyl(2,4,6-trimethoxyphenyl)iodonium benzoate (7ba)

^{13}C NMR (100 MHz, $CDCl_3$)

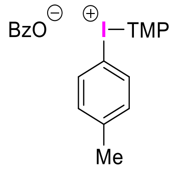

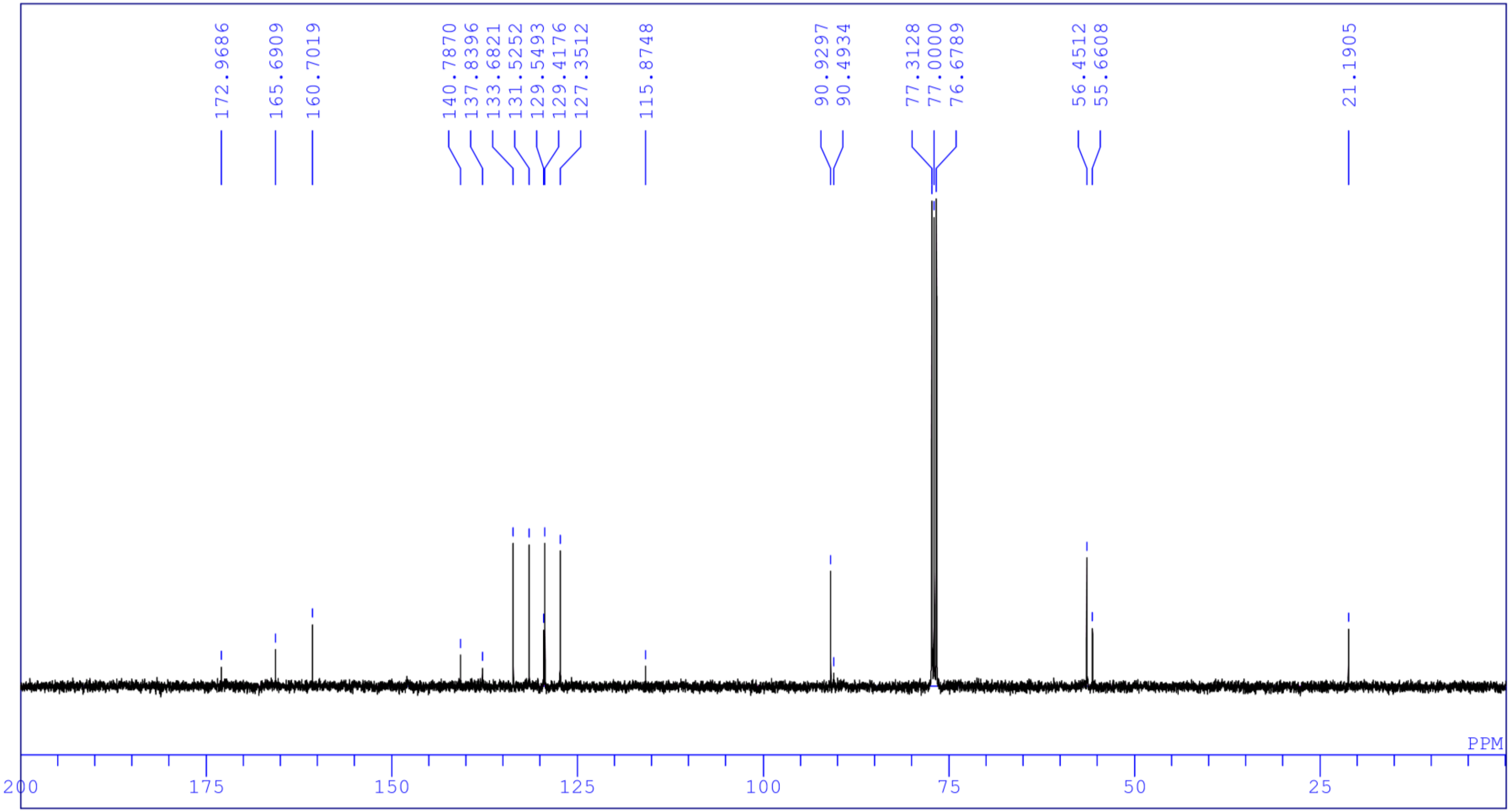

4-Methoxylphenyl(2,4,6-trimethoxyphenyl)iodonium benzoate (7ca)

^{1}H NMR (400 MHz, CDCl$_3$)

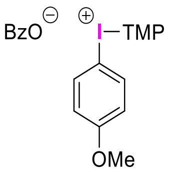

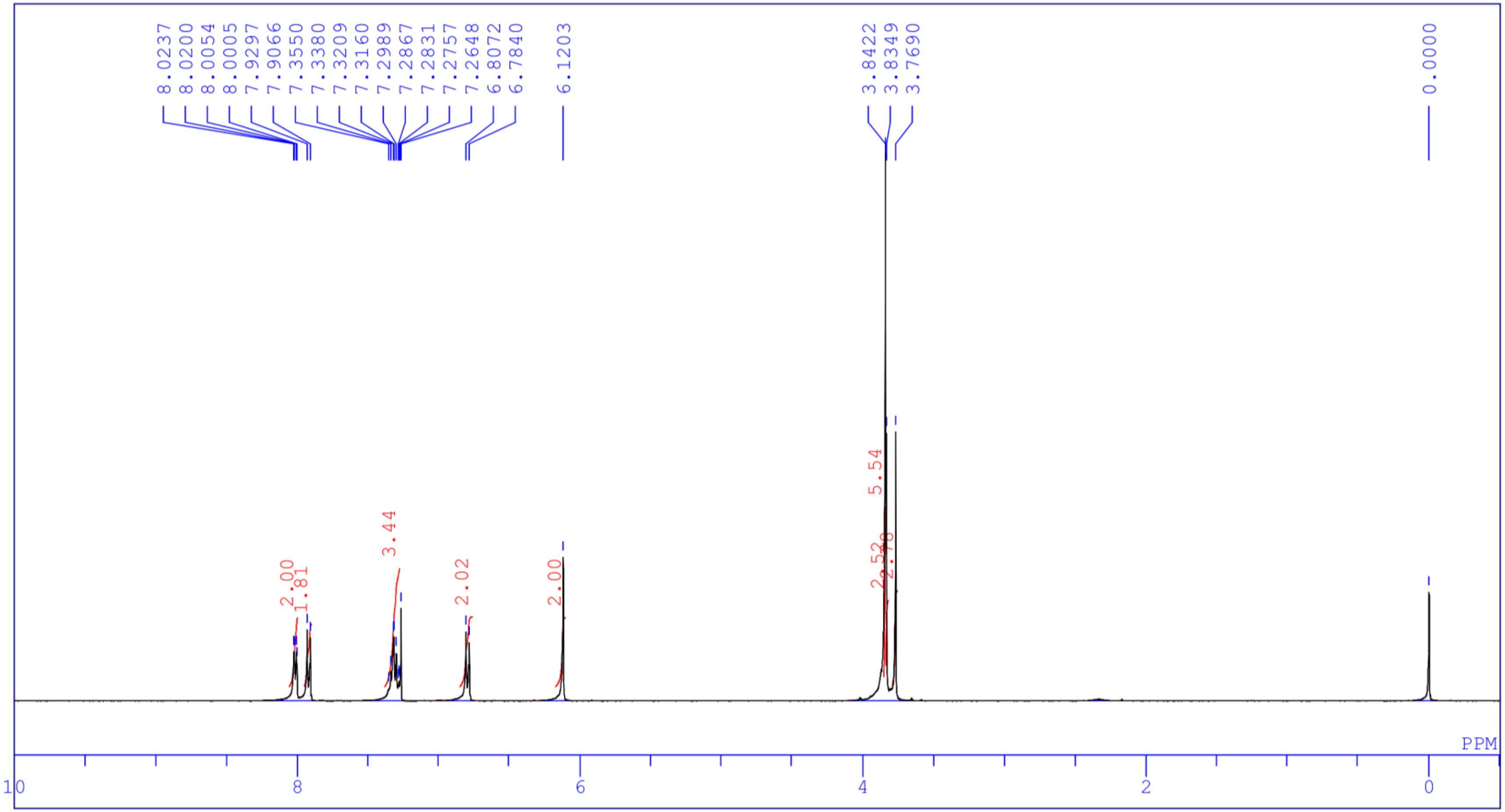

4-Methoxylphenyl(2,4,6-trimethoxyphenyl)iodonium benzoate (7ca)

^{13}C NMR (100 MHz, $CDCl_3$)

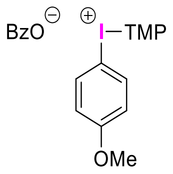

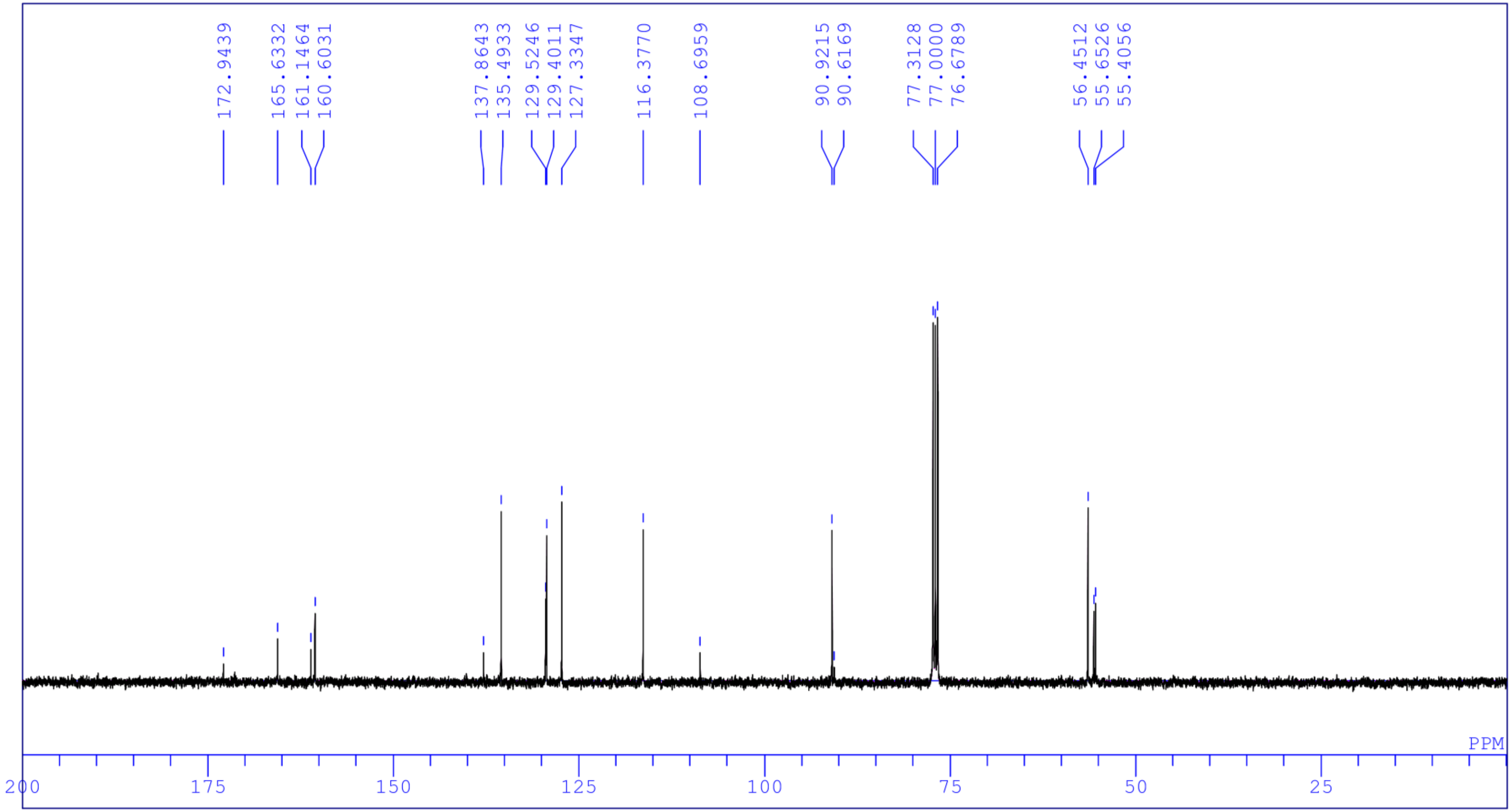

4-Fluorophenyl(2,4,6-trimethoxyphenyl)iodonium benzoate (7da)

^{1}H NMR (400 MHz, $CDCl_3$)

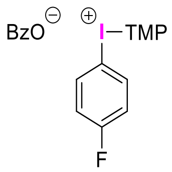

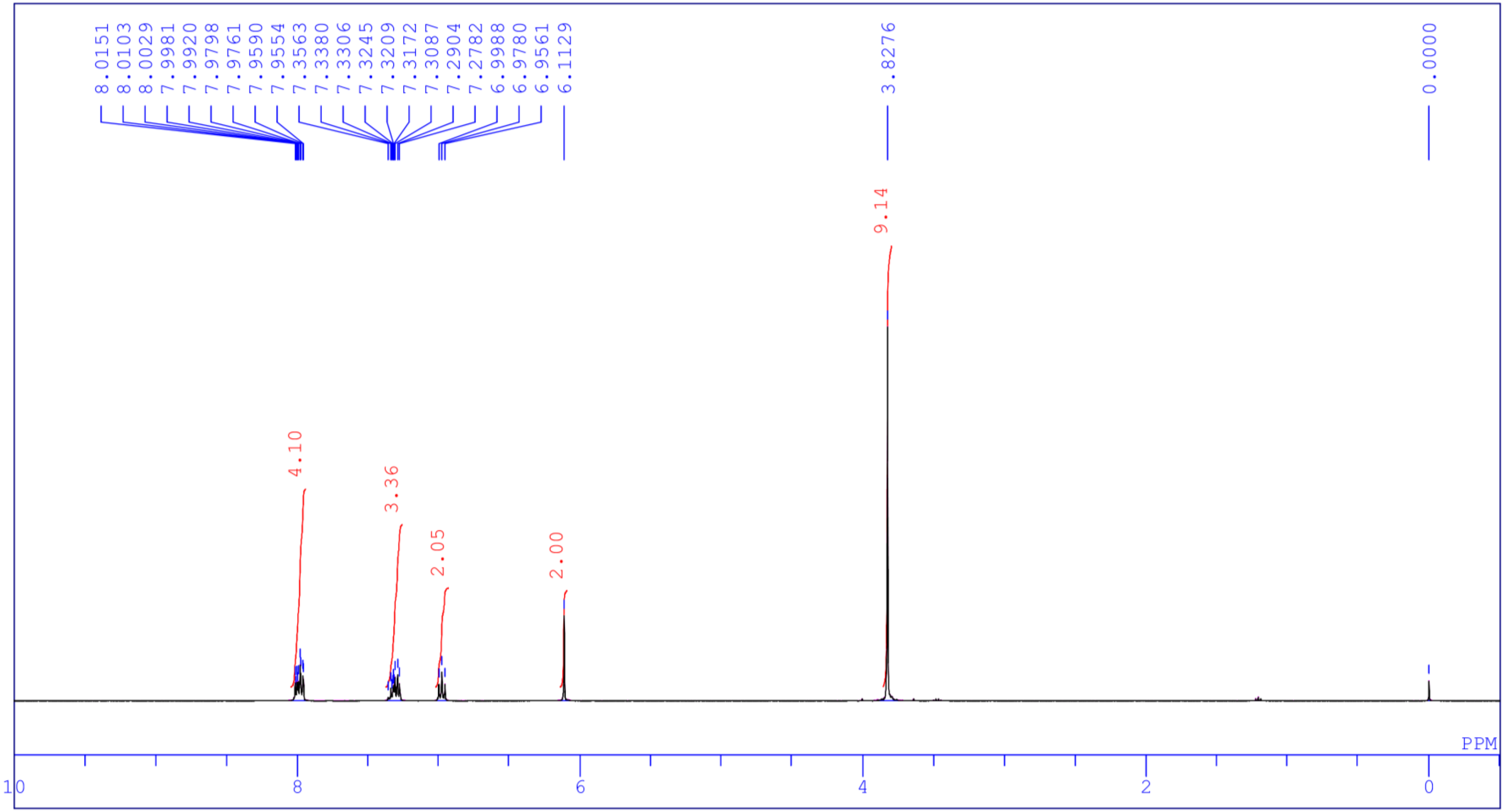

4-Fluorophenyl(2,4,6-trimethoxyphenyl)iodonium benzoate (7da)

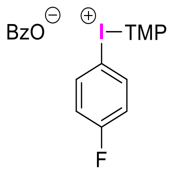

^{13}C NMR (100 MHz, CDCl$_3$)

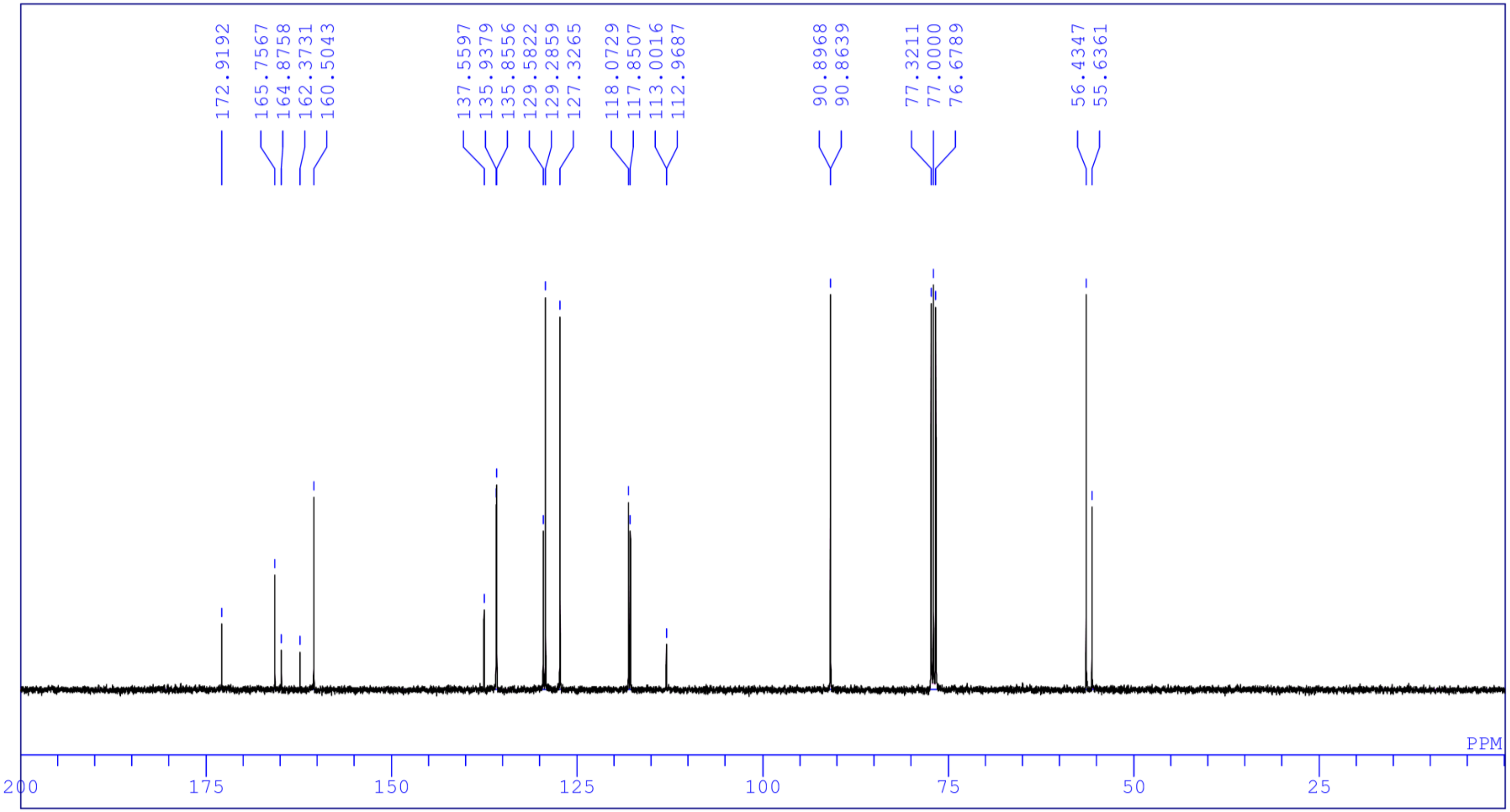

4-Fluorophenyl(2,4,6-trimethoxyphenyl)iodonium benzoate (7da)

^{19}F NMR (376 MHz, CDCl$_3$)

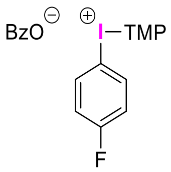

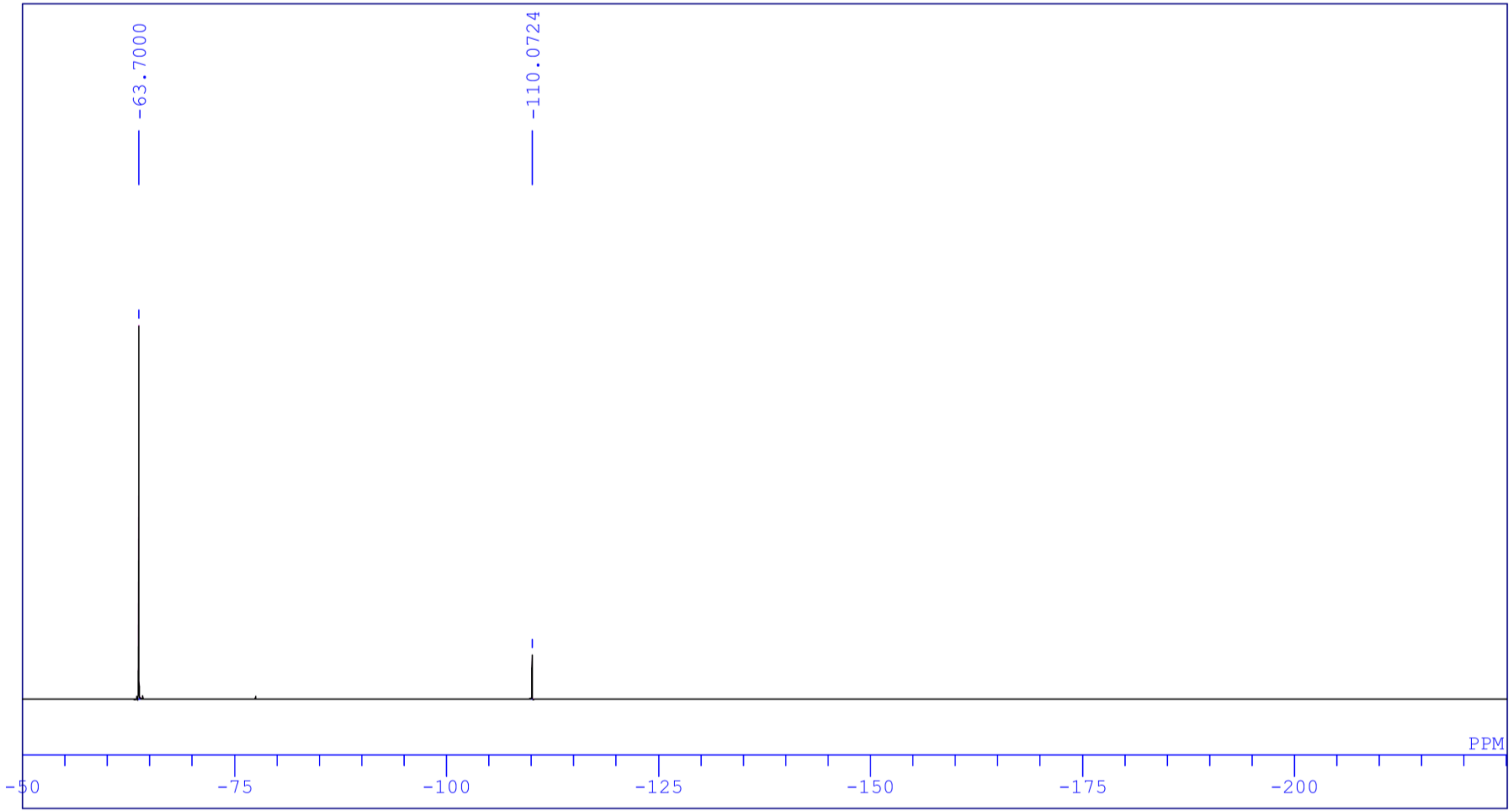

4-Bromophenyl(2,4,6-trimethoxyphenyl)iodonium benzoate (7ea)

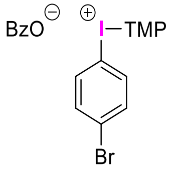

^{1}H NMR (400 MHz, $CDCl_3$)

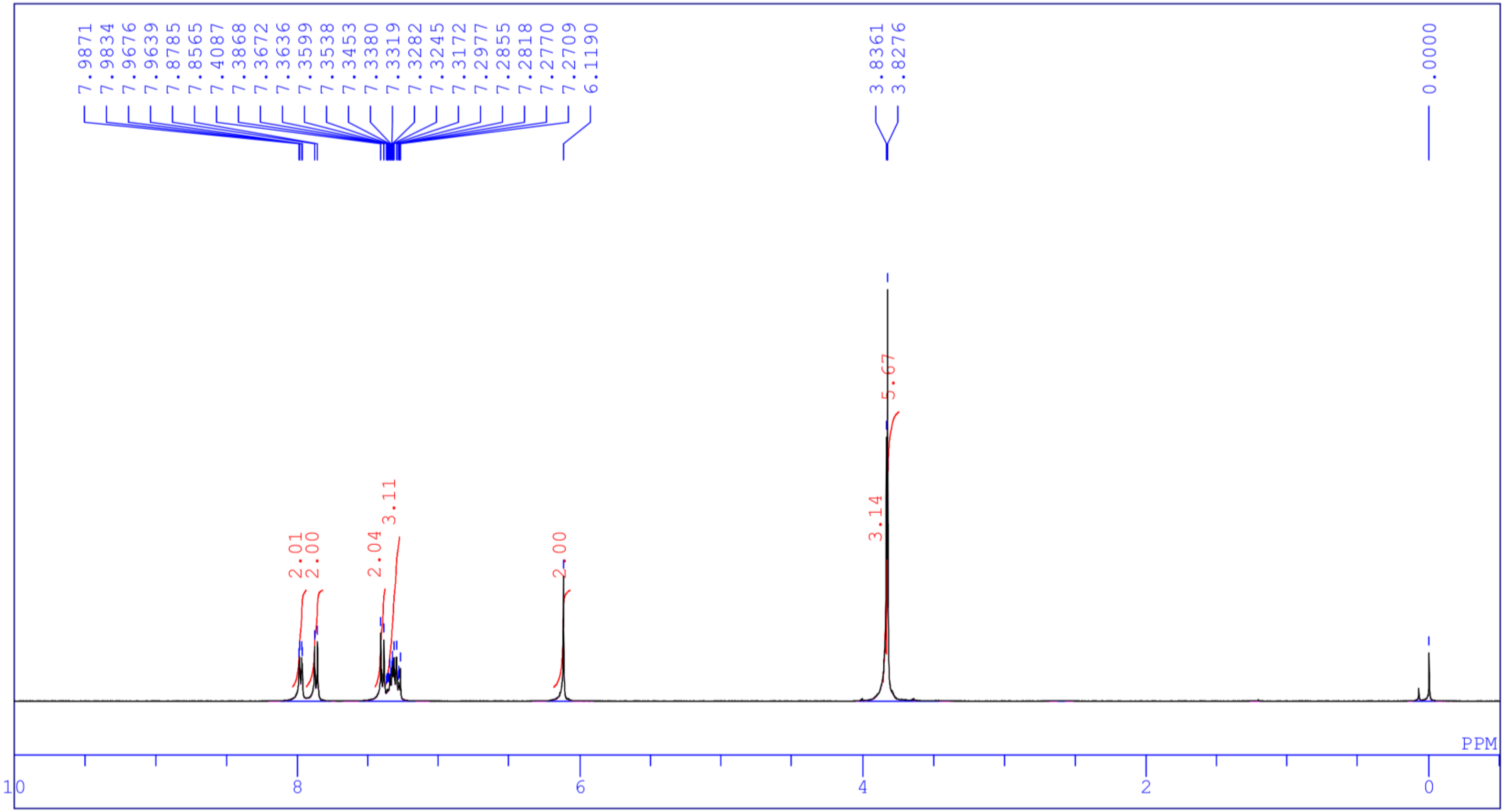

4-Bromophenyl(2,4,6-trimethoxyphenyl)iodonium benzoate (7ea)

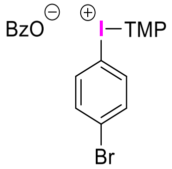

^{13}C NMR (100 MHz, $CDCl_3$)

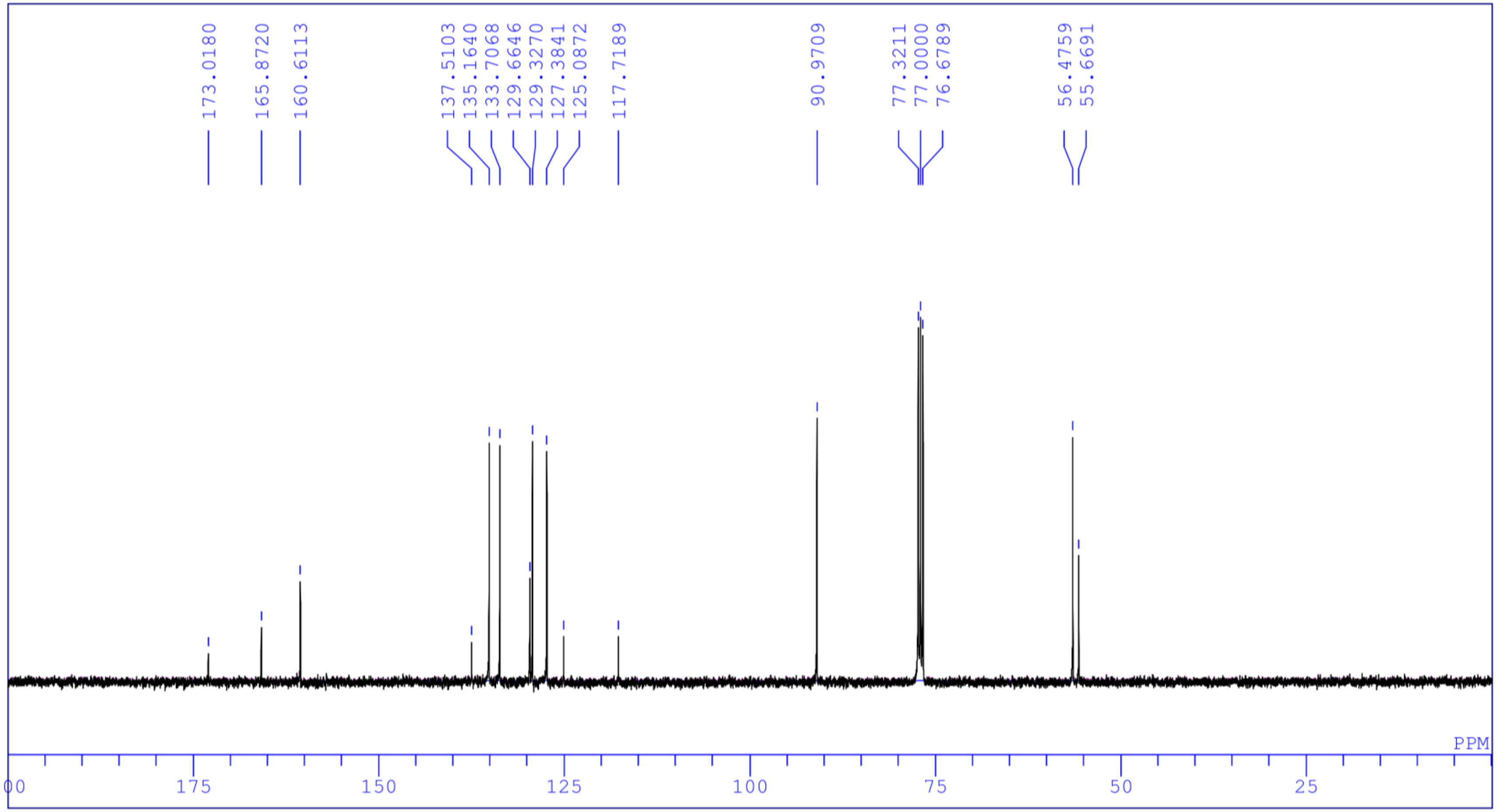

2,4,6-Trimethylphenyl(2,4,6-trimethoxyphenyl)iodonium benzoate (7fa)

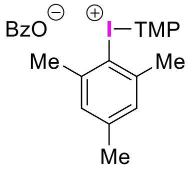

^{1}H NMR (400 MHz, $CDCl_3$)

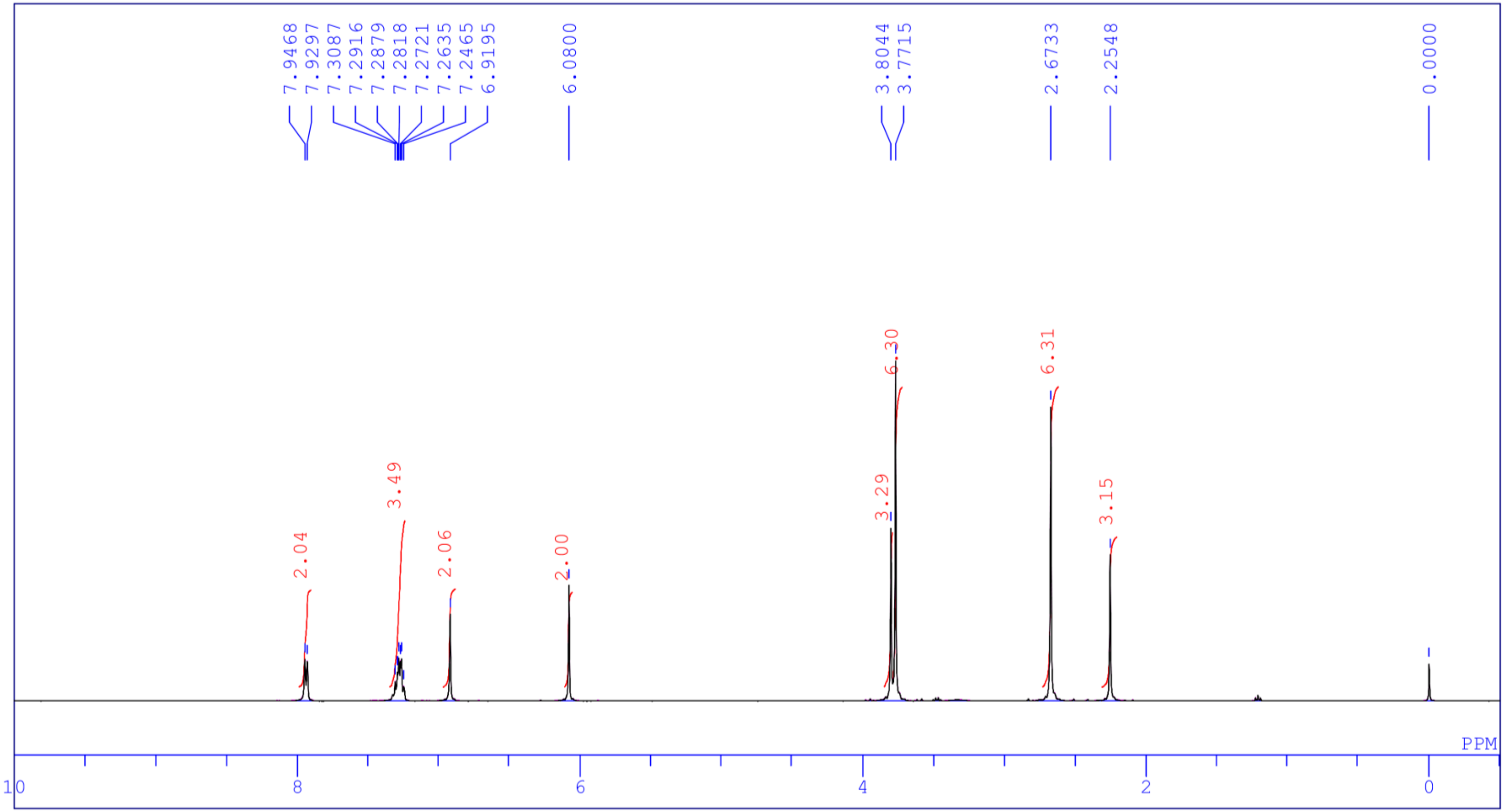

2,4,6-Trimethylphenyl(2,4,6-trimethoxyphenyl)iodonium benzoate (7fa)

^{13}C NMR (100 MHz, $CDCl_3$)

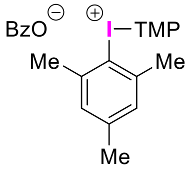

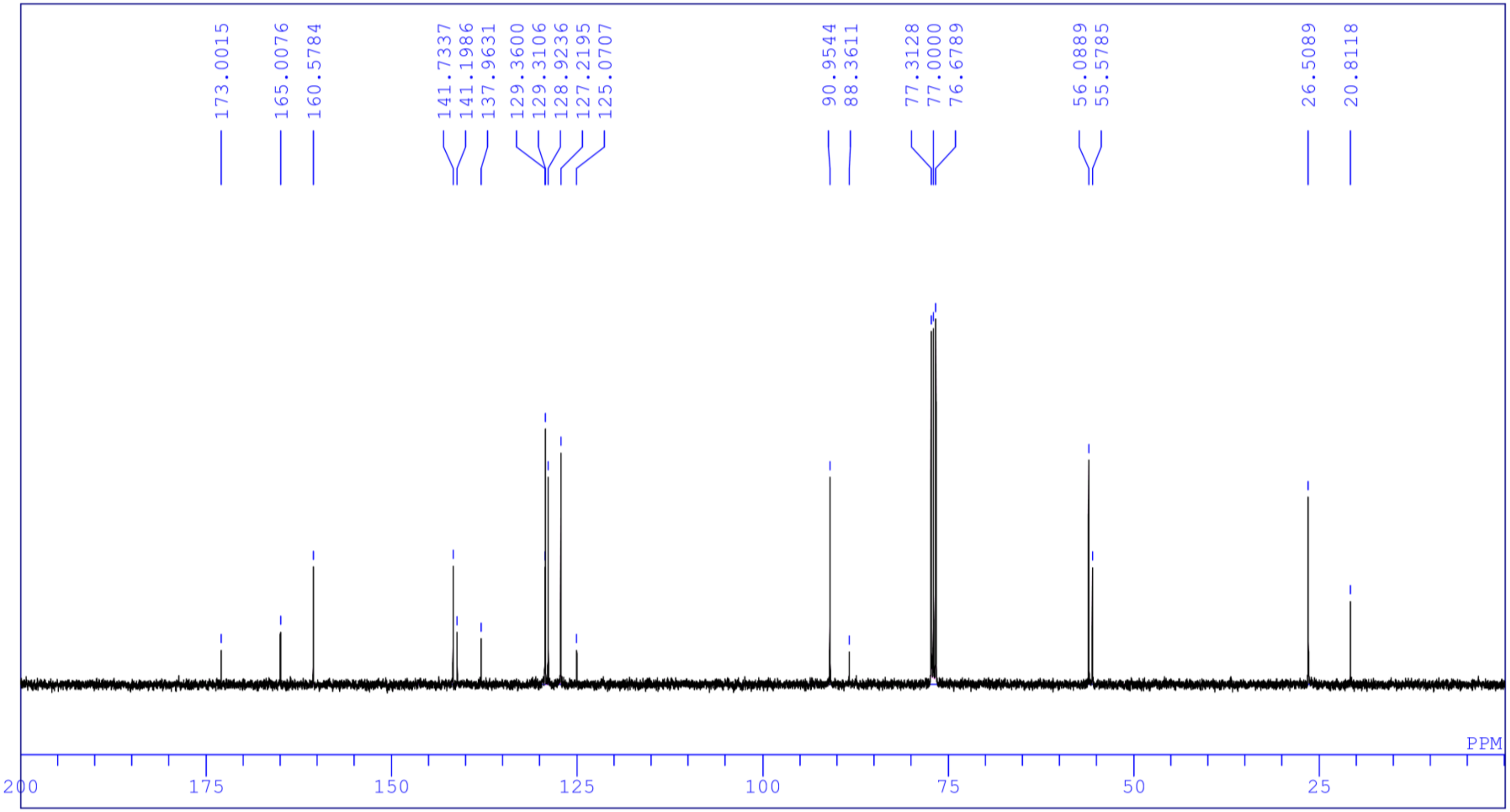

Phenyl benzoate (8aa)

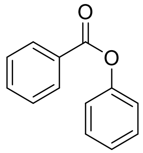

^{1}H NMR (400 MHz, $CDCl_3$)

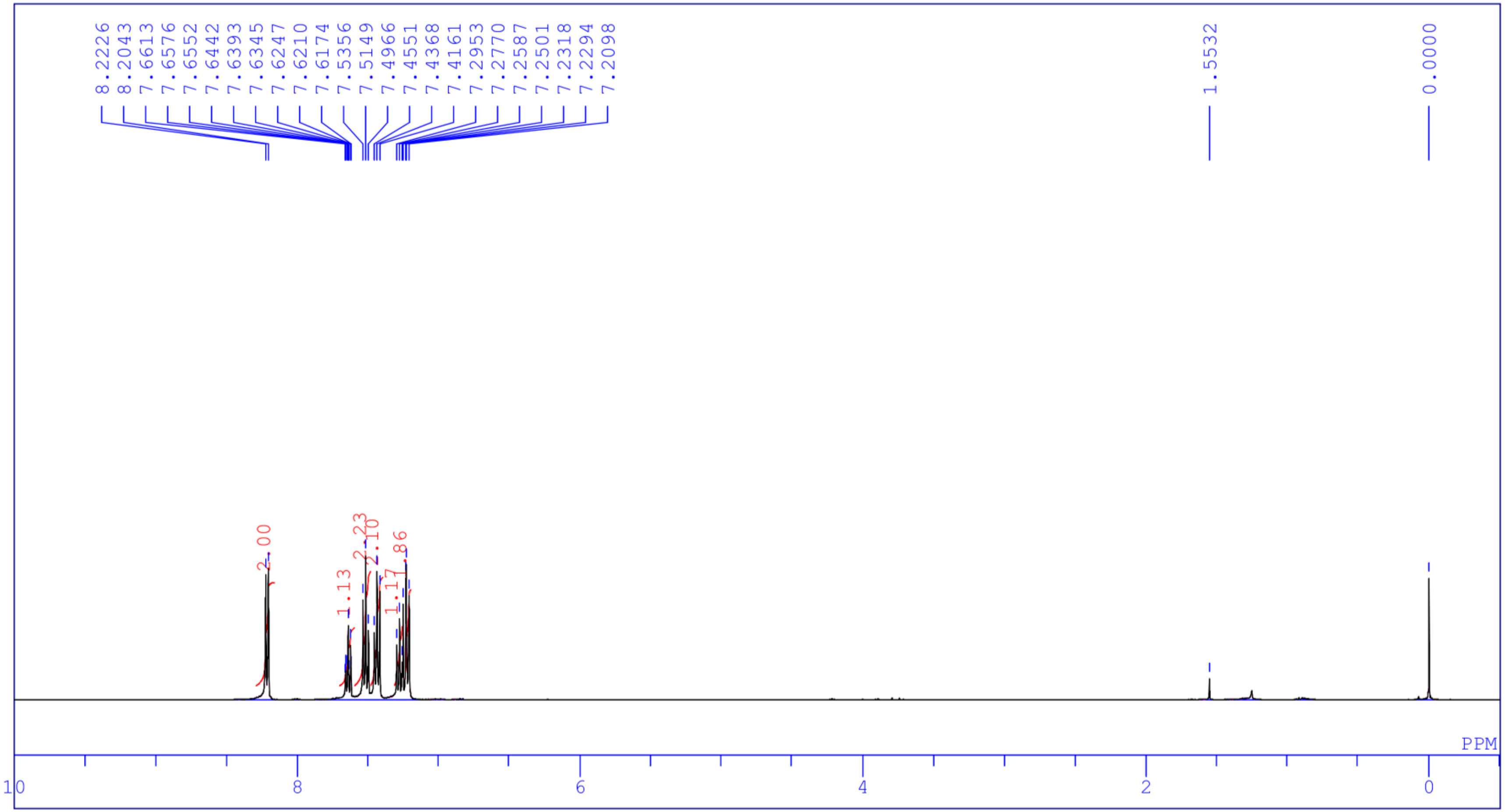

Phenyl benzoate (8aa)

^{13}C NMR (100 MHz, $CDCl_3$)

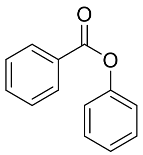

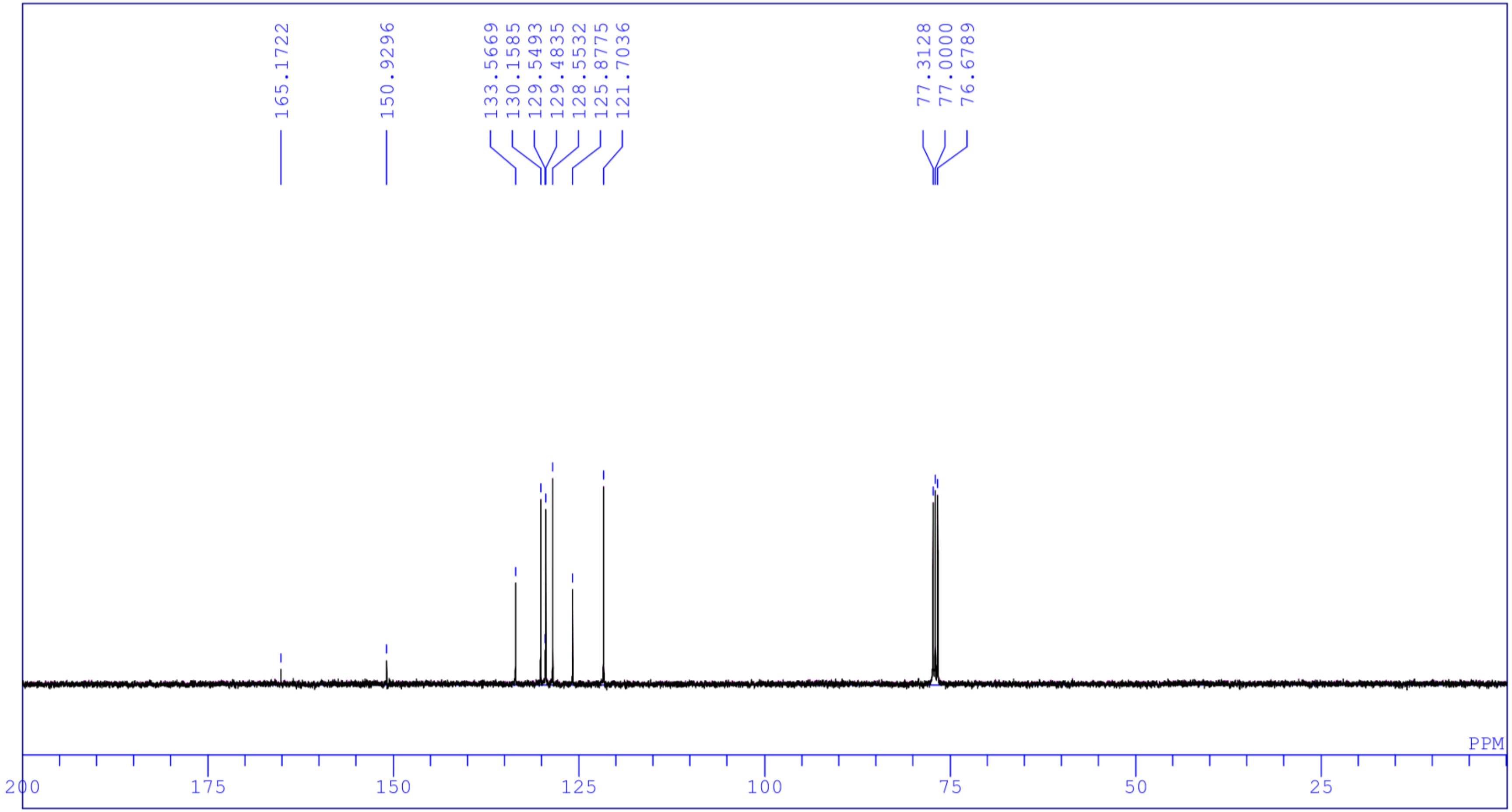

1-*tert*-Butyl-4-phenoxybenzene (10a)

^{1}H NMR (400 MHz, $CDCl_3$)

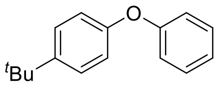

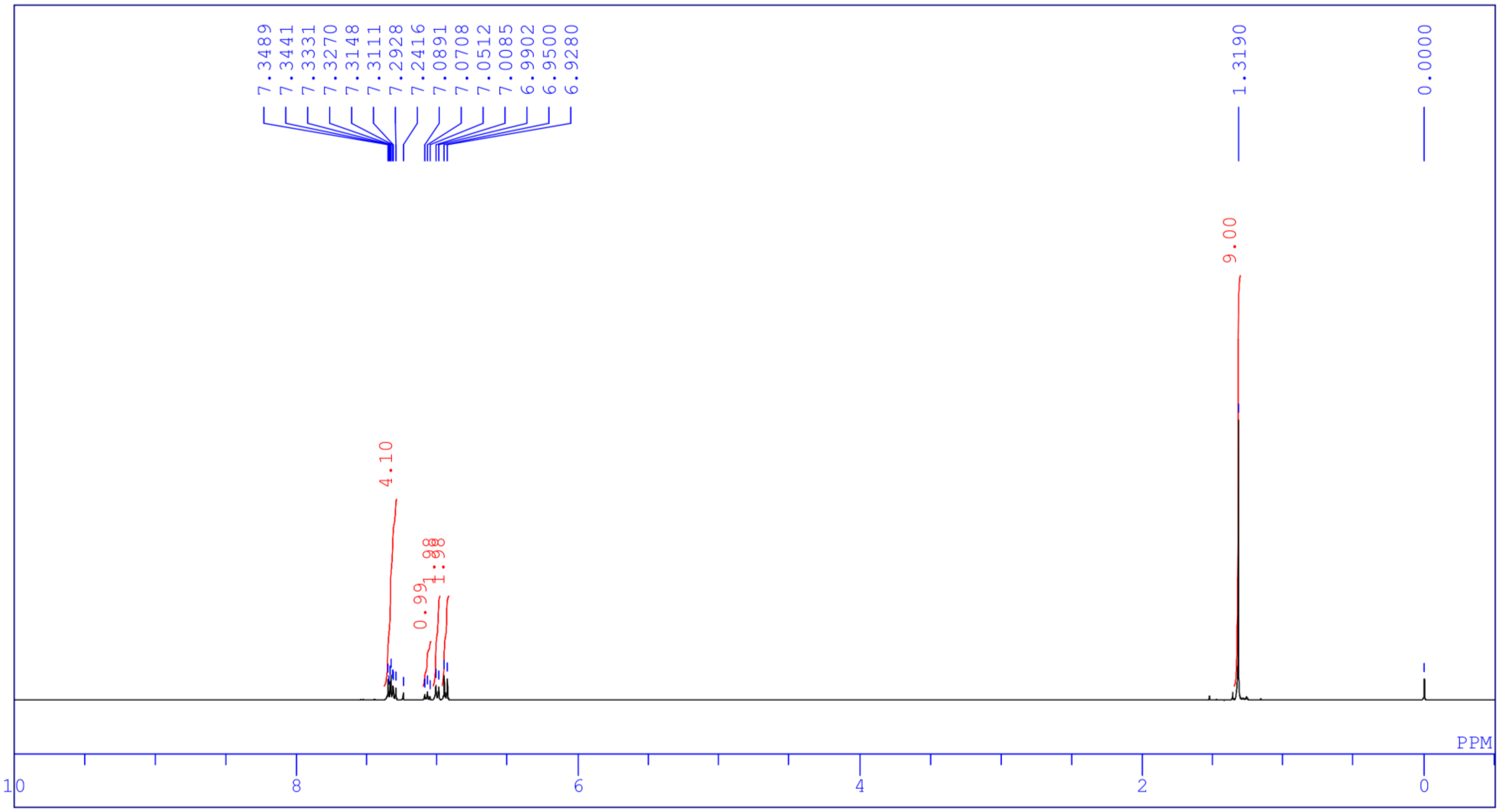

1-*tert*-Butyl-4-phenoxybenzene (10a)

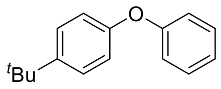

^{13}C NMR (100 MHz, $CDCl_3$)

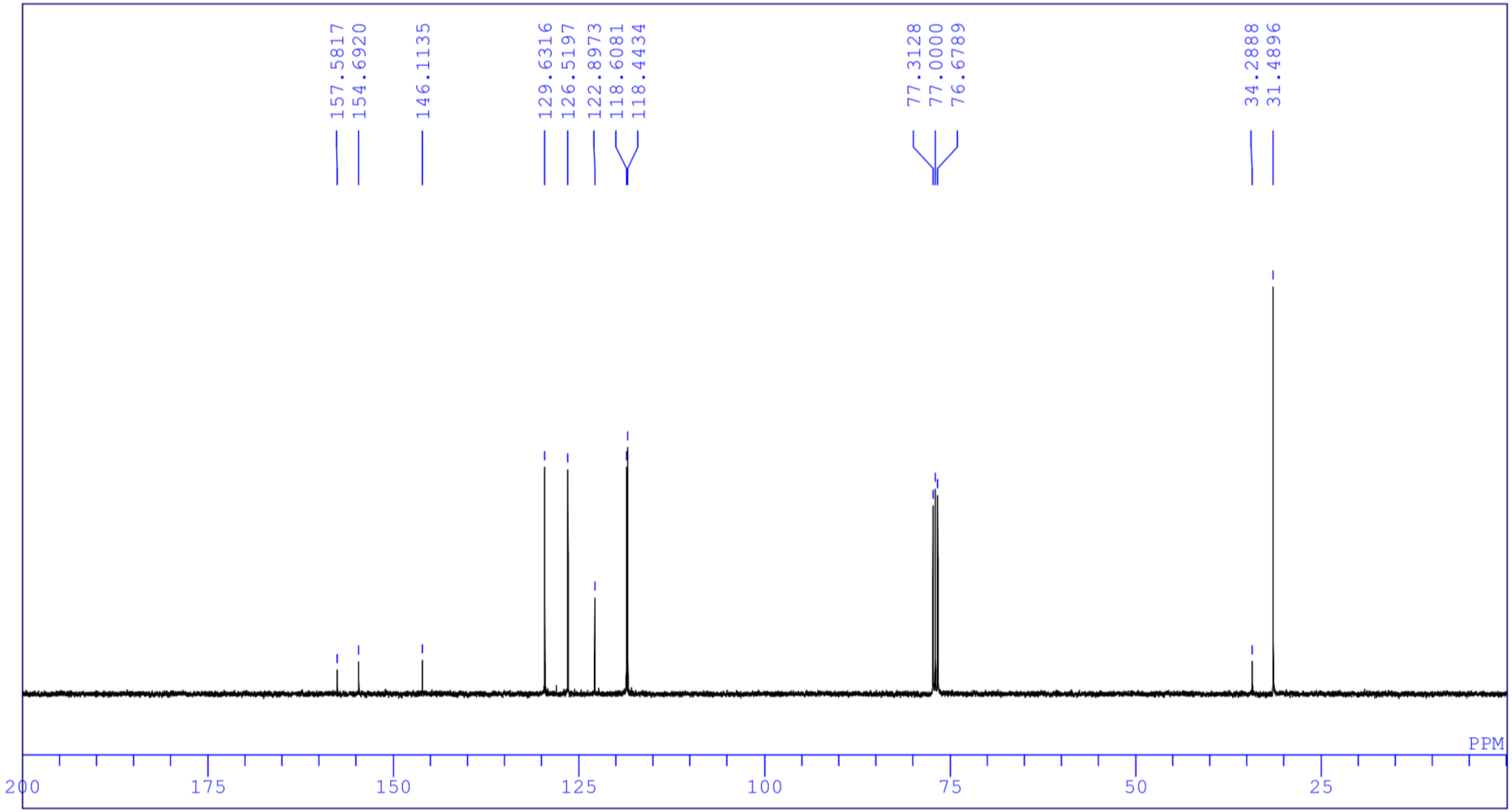

***N*-Methoxy-4-methyl-*N*-phenylbenzenesulfoneamide (12a)**

^{1}H NMR (400 MHz, CDCl3)

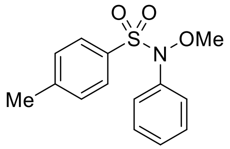

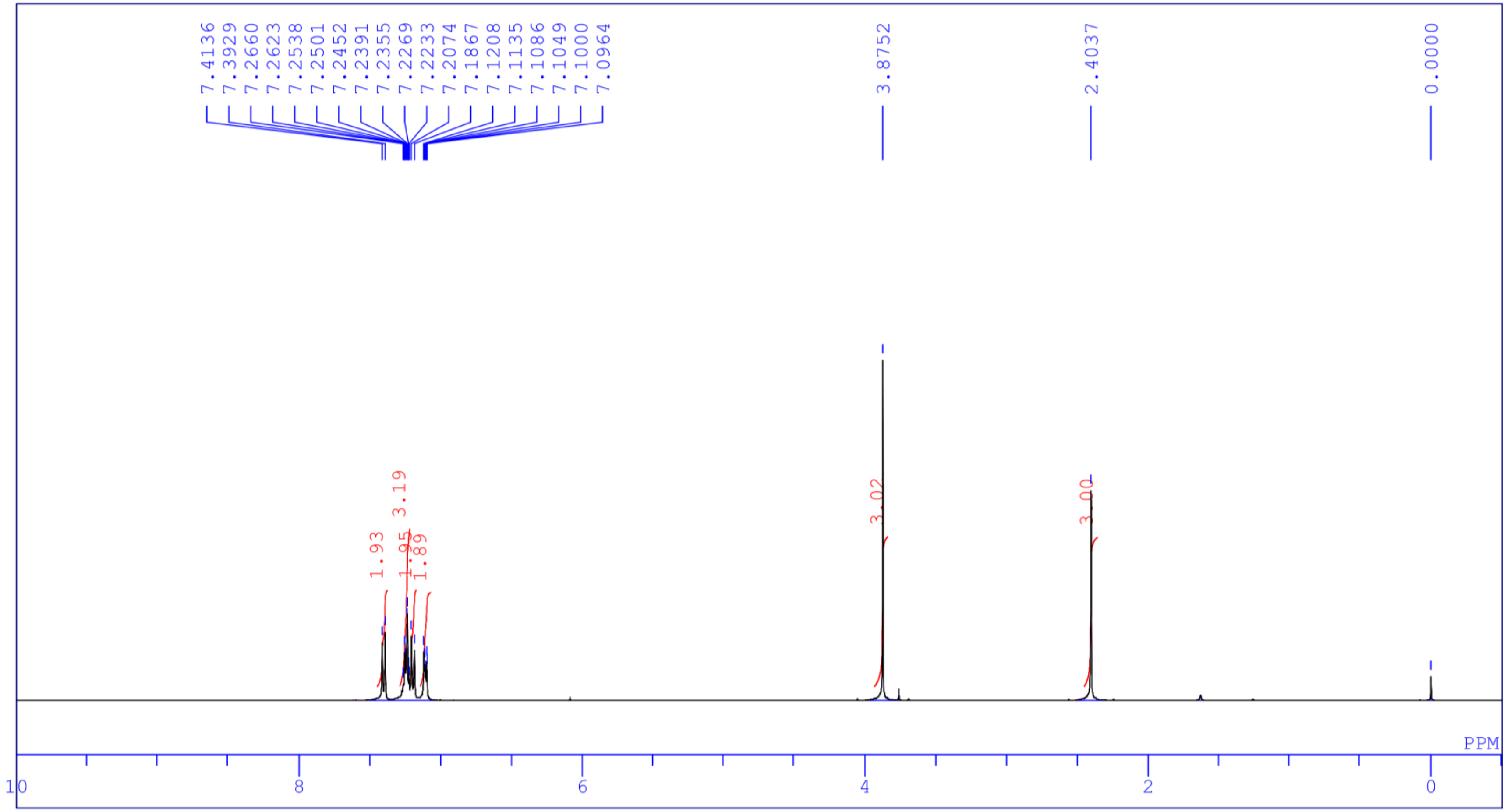

***N*-Methoxy-4-methyl-*N*-phenylbenzenesulfoneamide (12a)**

^{13}C NMR (100 MHz, $CDCl_3$)

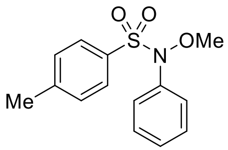

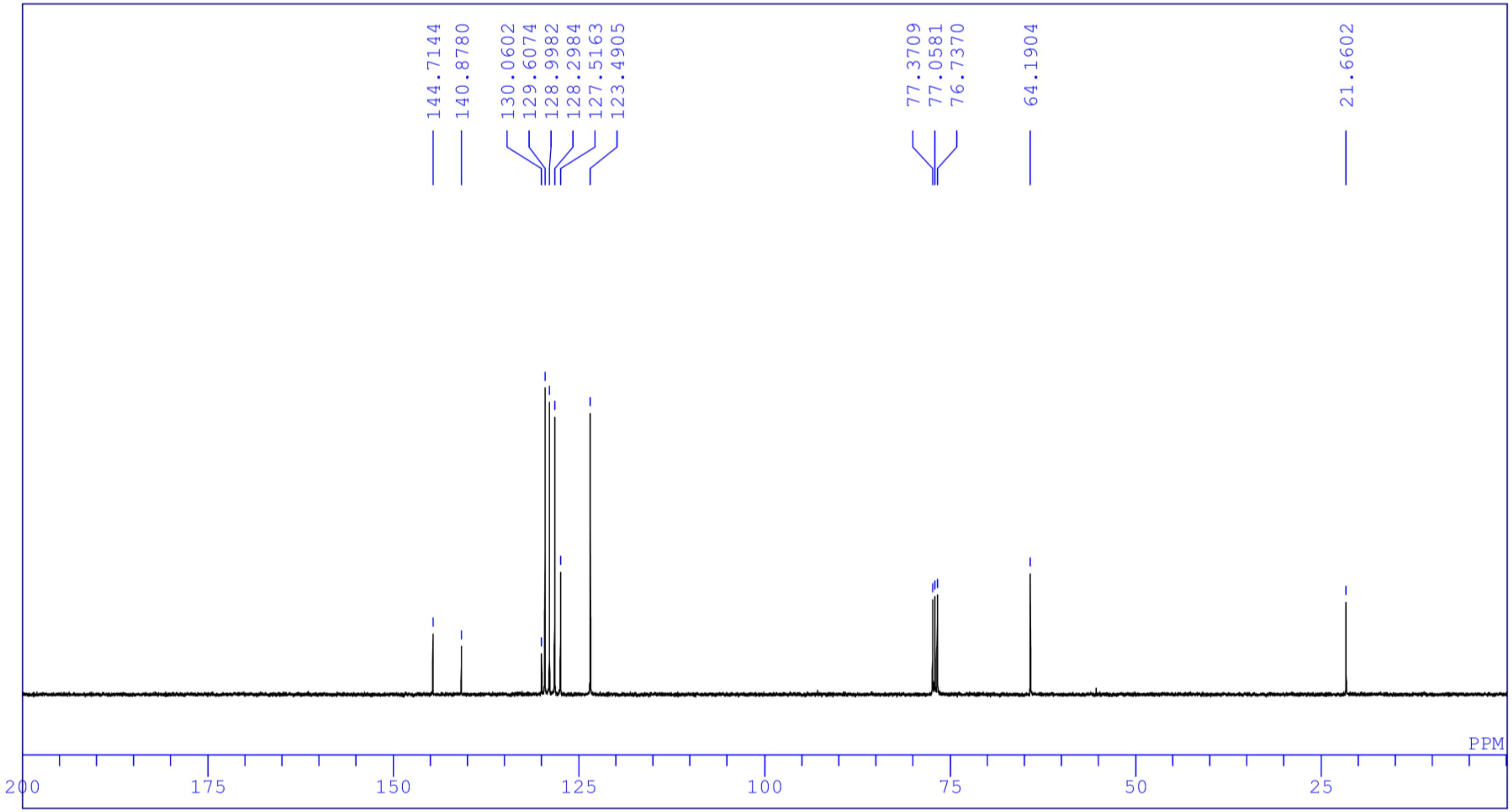

Supplement: File 1 — Further experimental details and copies of 1H, 13C, and 19F NMR spectra. [file Beilstein_J_Org_Chem-20-1020-s001.pdf]
